# Supplementary material for: Predictors of perinatal mortality in the seven major hospitals of Lusaka Zambia: A case control study
Source: PLOS Glob Public Health. 2025 Aug 20;5(8):e0003326. doi: 10.1371/journal.pgph.0003326 (PMC12367193; doi:10.1371/journal.pgph.0003326)
Supplement: S1 File — (S1_File.DOCX) [file pgph.0003326.s001.docx]

**PARTICIPANT INFORMATION SHEET**

**Title of the Research Study**

Burden and predictors of perinatal mortality in Lusaka district, Lusaka, Zambia.

**Principal Investigator**

Musonda Makasa, PhD in Epidemiology Candidate, School of Public Health, Department of Epidemiology and Biostatistics, University of Zambia.

Contact: +260 978888435

Email: mcmakasa@gmail.com

**Purpose and Background**

The United Nations adopted an agenda for sustainable development goals and targeted reduction of Stillbirth mortality to 12 per 1000 live births by 2030. A Meta – analysis by Blencowe *et al*. (2016) reported that there has been global a decline from the year 2000 to 2015 from 24.7 to 18.4 per 1000 live births. Despite this progress, almost half (45%) of the data was from the higher income countries, which accounts for less than 2% of the world’s total stillbirths (Blencowe *et al.*, 2016). A meta-analysis of demographic and health surveys in SSA revealed that the region has one of the highest levels of PNM globally with Zambia reported to be 33 per 1000 live births (Central Statistics Office (Zambia), 2018; Akombi and Renzaho, 2019).

Therefore, important that the trend of perinatal mortality is investigated through systematic review and meta-analysis in Zambia. The findings of this will help to understand magnitude and burden of perinatal mortality including trends, causes, and whether there has been positive change or worsening. Secondly, it is timely to the predictors of perinatal mortalities in the selected facilities in Lusaka, Zambia. The incorporation of post-mortem and placenta histopathology will provide more information regarding pathological causes and infections.

**Procedures:**

For the systematic review ethical clearance will be sought from UNZABREC and the NHRA. Whereas for study 2 and 3 also after ethical and NHRA clearance, if consent to participate is obtained, the following things will happen:

1. We will review the relevant information from your file and hospital records. In an event of inadequate information from the patient file and records. You will undergo which will be carried out at the respective study sites. The interview will involve inquiring details about your basic biological, demographic, medical and socioeconomic situation.
2. You will not be exposed to anything outside standard medical management and treatment of your condition.
3. The information obtained is not intended and will not be used for monetary gain.
4. Your name and other detail will strictly be kept confidential, anonymised and solely research purposes. You will be assigned a study number which will be assigned to the questionnaires.

**Benefits**

1. The systematic review and meta-analysis will help us evaluate the trends since 2000 and scope out the problems.
2. Study 2 will help us evaluate the burden, magnitude and scope out the proportion and predictors of perinatal mortality.
3. Study 3 on stillbirths will not only help us evaluate the predictors but understand further the probable cause from the histopathology of placental tissues.

**Risks**

There is not major risk that is anticipated by conducting systematic review and meta-analysis since this wholly be review of secondary data. For study 2 and 3, the risk of emotional distress is possible but to mitigate this the process will be preceded by psychosocial and bereavement counselling according to standard practice following bereavement. Secondly, the search from files and records will be as extensive as possible in order to shorten the time of interaction and effectively reduce on questions for the participant...

**Reimbursements**

A K5 will be set aside as compensation for taking time to participate in the activity.

**Confidentiality**

The results of all the study will be discussed with you, and kept confidential unless you wish otherwise). Except for this disclosure, all information obtained in this study will be considered confidential and used only for research purposes. You identity will be kept confidential as far as the law allows.

**Injury clause**

In an event of suffering emotional distress as a result of being in this study, treatment will be made available at the study site. The costs of such treatment will be paid by the researcher. For further information about this, you can contact the chairperson of Research Biomedical Ethical committee of the University of Zambia, School of Medicine on telephone number 256067 or P.O BOX 50110, Ridgeway campus, Lusaka

**Questions**

**.............................................,** the researcher has discussed this information with you and offered to answer your questions. If you have further questions, you can contact him on 0977349386 or the Chairperson of Research Biomedical Ethics Committee at University of Zambia, School of Medicine on telephone 0211 256067.

**INFORMED CONSENT**

I confirm that I have understood the information I have been given about the study. I agree to participate in this study. I confirm that I am joining the study of my free will and I can withdraw at any time without affecting the care available to me. I understand what will be required of me.

Participant’s Signature......................................... Signed or thumb Print.................................

Date.................................................

Witness (Name and Signature) ...............................................

Date**.......................................**

I confirm that I have explained the information fully and answered any questions.

Signed for the study team
