# Supplementary material for: Predictors of perinatal mortality in the seven major hospitals of Lusaka Zambia: A case control study
Source: PLOS Glob Public Health. 2025 Aug 20;5(8):e0003326. doi: 10.1371/journal.pgph.0003326 (PMC12367193; doi:10.1371/journal.pgph.0003326)
Supplement: Attachment — Submitted filename: Response to Reviewers.pdf [file pgph.0003326.s003.pdf]

Dr Musonda Makasa

P.O Box 310129

Lusaka

21st October 2024

PLOS Global Public Health Staff
globalpubhealth@plos.org

Dear Editor,

Rebuttal Letter: Response to Reviewer #3 and #4 Comments PLOS Global Public Health Decision: Revision required [PGPH-D-24-01110R1]

We are grateful for the valuable comments provided by both Reviewer #3 and Reviewer #4, which have contributed significantly to improving the clarity and overall quality of our manuscript titled "Predictors of Perinatal Mortality in the Seven Major Hospitals of Lusaka Zambia: A Case-Control Study." We have carefully considered each remark and have made the necessary revisions. Below illustrates a point-by-point response to all the comments, highlighting the changes made in the manuscript:

Response to Reviewer #3

1. Remark(s) 1: Need for Revision of Sampling Procedure Justification
Response:
Thank you for this observation. We have now revised the sampling methodology to clearly explain the reasoning behind the selection of cases and controls. The sampling method is based on the availability of cases and controls within the 24-hour window of a shift to minimize selection bias. We also elaborate on the 1:2 case-to-control ratio used in the study.

2. Remark(s) 2: Clarify Whether Data was Missing Completely at Random
Response:
We have now included a sentence in the manuscript specifying that missing data was random.

3. Remark(s) 3: Justification of Sample Size Assumptions
Response:
We have provided further clarification in the "Study Design" section regarding the assumptions made for the sample size calculation. We now detail the anticipated effect sizes and power estimates that were used to inform the sample size determination.

4. Remark(s) 4: Use of Appropriate Terminology for Logistic Regression
Response:
Thank you for your recommendation. We have revised the language in the "Data Analysis"

section to reflect that odds ratios were computed using logistic regression analysis. We have corrected the terminology throughout the manuscript.

5. Remark(s) 5: Address Any Potential Collinearity Between Variables
Response:
We agree with your suggestion and have included a mention of the multicollinearity diagnostics that were conducted. This has been added to the "Data Analysis" section.

6. Remark(s) 6: Discussion of Key Results
Response:
The "Discussion" section has been thoroughly revised to present the study's key findings first, followed by comparisons with relevant literature. We have now clearly distinguished between our study findings and previous studies to improve the flow and focus on the study's contributions.

Response to Reviewer #4

1. Remark(s) 1: Title Reads Fine
Response:
We appreciate your comment and confirm that the title remains unchanged.

2. Remark(s) 2: Correction of Article in Abstract
Response:
We have corrected the word "this" to "This" at the beginning of the sentence in the "Methods" section of the abstract.

3. Remark(s) 3: Replace the Word "Proportion" in Keywords
Response:
We have replaced the keyword “proportions” with “risk factors” to better align with the study focus.

4. Remark(s) 4: Replacing the Word "Avoid" in the Introduction
Response:
The word “avoid” has been replaced with “prevent” in the introduction to improve clarity.

5. Remark(s) 5: Recasting Sentence in the Introduction
Response:
We have reworded the sentence to read: “For example, until recently, most stillbirths were not accounted for in global data tracking, while a lack of social recognition, investment, and programmatic action have contributed to this issue.”

6. Remark(s) 6: Provide Sources for Literature Showing Linkage Between Maternal Deaths and Perinatal Mortality
Response:

We have added the relevant citations immediately after the sentence “Literature shows a strong linkage between maternal deaths and perinatal mortality” to provide appropriate references.

7. Remark(s) 7: Rephrase Maternal and Perinatal Death Causes Sentence
Response:
The sentence has been rewritten to: “Almost two-thirds of the causes of maternal death also account for perinatal deaths.”

8. Remark(s) 8: Clarification of Early Neonatal Mortality Definition
Response:
We have rephrased the sentence to: “Early neonatal mortality, a subset of perinatal mortality, refers to the death of a newborn within the first 7 days of life.”

9. Remark(s) 9: Correcting Reference to WHO SDG in the Introduction
Response:
The phrase has been corrected to: “The World Health Organization’s Sustainable Development Goal (SDG) 3.2 targets to end…”

10. Remark(s) 10: Replace the Word “Demonstrated”
Response:
We have replaced the word “demonstrated” with “showed” in the appropriate sentence in the introduction.

11. Remark(s) 11: Replace the Word "Realistic" in the Introduction
Response:
The word “realistic” has been replaced with “possible” as suggested.

12. Remark(s) 12: Citation Correction for Akombi and Renzaho
Response:
We confirm correction for the Akombi and Renzaho authorship and references have been updated the citations accordingly.

13. Remark(s) 13: Replace the Word "Attaining"
Response:
We have replaced “attaining” with “meeting” in the relevant section.

14. Remark(s) 14: Recasting Unclear Sentence in Materials and Methods
Response:
We have rephrased the sentence in the "Materials and Methods" section for better clarity: “Participants were prospectively recruited as cases occurred, and those who agreed to participate were included in the study.”

15. Remark(s) 15: Correcting Tense in Materials and Methods
Response:
The word “share” has been corrected to “shared” in the appropriate section.

16. Remark(s) 16: Inserting the Article "The" in Materials and Methods
Response:
We have inserted the missing article “the” in the sentence in the "Materials and Methods" section.

17. Remark(s) 17: Rewriting Incomplete Sentence in Study Design
Response:
We have revised the sentence to: “After sharing the participant information sheet and obtaining informed consent, eligible participants were enrolled in the study.”

18. Remark(s) 18: Rewriting Figure 1.0 Reference
Response:
The sentence has been revised to: “Figure 1.0 illustrates the source of the sample, the eligibility screening process, and the final sample size determination.”

19. Remark(s) 19: Removing Repetition in Inclusion Criteria
Response:
We have removed the redundant phrase “of gestation” from the inclusion criteria.

20. Remark(s) 20: Redefining Exclusion Criteria
Response:
We have redefined the exclusion criteria to cover participants who qualified for the study but were unable to participate due to reasons such as absence during the study period or being too unwell to participate.

21. Remark(s) 21: Rewriting Sampling Technique Section
Response:
The entire section on "Sampling Technique" has been rewritten to ensure clarity. We consulted a language expert to ensure the content is free of grammatical errors and ambiguities.

22. Remark(s) 22: Revising Data Collection Tool Terminology
Response:
The term “interviewer administered” has been replaced with “researcher administered” to avoid confusion.

23. Remark(s) 23: Recasting Sentences in Data Analysis Section
Response:
We have rewritten the "Data Analysis" section to ensure the sentences are clear, complete, and accurate.

24. Remark(s) 24: Expanding Ethical Considerations
Response:
We have added details regarding how confidentiality and privacy were maintained, including anonymizing data and secure storage of sensitive information.

25. Remark(s) 25-39: Addressing Grammar and Structure Issues in Results and Discussion
Response:
We have undertaken a comprehensive review of the "Results" and "Discussion" sections and corrected grammatical errors, subject-verb disagreements, and improved sentence structure. Additionally, we have revised these sections to focus more on the findings of the current study while making appropriate comparisons to previous literature.

40. Remark(s) 40: Improving the Strengths and Limitations Section
Response:
We have revised this section to ensure clarity and readability. The sentence structure has been improved, and the content now better reflects the limitations of the study.

41. Remark(s) 41: Enhancing the Recommendations Section
Response:
We have restructured the "Recommendations" section, clearly addressing specific stakeholders such as healthcare policymakers and practitioners, and providing actionable suggestions for reducing perinatal mortality in Lusaka, Zambia.

We hope that the revisions sufficiently address the concerns raised by both reviewers and improve the manuscript's quality. We appreciate your time and consideration.

Sincerely,

Musonda Makasa

***Corresponding author**
