# Supplementary material for: Predictors of perinatal mortality in the seven major hospitals of Lusaka Zambia: A case control study
Source: PLOS Glob Public Health. 2025 Aug 20;5(8):e0003326. doi: 10.1371/journal.pgph.0003326 (PMC12367193; doi:10.1371/journal.pgph.0003326)
Supplement: Attachment — Submitted filename: Response to reviewers .pdf [file pgph.0003326.s005.pdf]

Dr Musonda Makasa

P.O Box 310129

Lusaka

November 5 2024

PLOS Global Public Health Staff
globalpubhealth@plos.org

Dear Editor,

Rebuttal Letter: Response to Reviewer #6 Comments for PGPH-D-24-01110R2
Predictors of perinatal mortality in the seven major hospitals of Lusaka Zambia: A Case Control Study
PLOS Global Public Health

We are grateful for the valuable comments provided by Reviewer #6, which have contributed significantly to improving the clarity and overall quality of our manuscript titled "Predictors of Perinatal Mortality in the Seven Major Hospitals of Lusaka Zambia: A Case-Control Study." We have carefully considered each remark and have made the necessary revisions. Below illustrates a point-by-point response to all the comments, highlighting the changes made in the manuscript:

Methodology

Study Design

- *Reviewer Comment*: "Precise if the study design was prospective case control."

  Response: We have clarified that our study design was a prospective case-control study.

- *Reviewer Comment*: "Take this sentence in sampling technique: 'due to rarity of the outcome purposeful sampling was used for the case that met the inclusive criteria.'"

  Response: We revised the sampling technique description to clarify that purposive sampling was used due to the rarity of perinatal mortality cases, and moved as advised.

Sampling Technique

- *Reviewer Comment*: "Put more information on how you used PPS: Probability Proportional to Size sampling. Was this technique used to select participants for each facility or just to select major hospitals? Also, consider whether proportionate stratified random sampling could better address representativity."

Response: To clarify, Probability Proportional to Size (PPS) sampling was applied specifically to allocate the sample across the seven major hospitals based on delivery volumes. Within each hospital, participants were selected according to the defined case and control criteria. This approach ensured proportional representation from each hospital based on patient volume while addressing logistical constraints. We have now clarified this process and rationale in the manuscript, explaining why PPS sampling was suitable for facility selection while purposive sampling was used within facilities.

Proportionate stratified random sampling can significantly enhance the representativeness of a sample, particularly when applied in contexts where there is a need to capture the diversity of different subgroups within a population.

- *Reviewer Comment*: "The final selected participants must correlate with the final sample size and findings total. Explain any discrepancies or apply a sample correction factor if necessary."

  Response: To ensure adequate power and account for potential non-responses or data quality issues, we initially oversampled, selecting 630 participants. This was a precautionary measure to help meet the targeted sample size of 558 participants, as it's common for some participants to be lost to follow-up, provide incomplete responses, or have data quality issues that might render some responses unusable.

  After data collection, we found that some records had to be excluded due to incomplete information, which could have resulted in a final analyzed sample of less than 558 participants. Using this approach helped ensure that our findings were both statistically valid and reflective of the intended sample size, despite initial oversampling.

Data Analysis

- *Reviewer Comment*: "Indicate that the chi-square test was used in data analysis and specify that all factors associated with perinatal death in bivariate analysis were transferred to logistic regression for confounder adjustment."

  Response: We have revised the data analysis section to specify the use of the chi-square test in bivariate analysis. Additionally, we clarified that variables with significant associations in bivariate analysis were included in logistic regression to adjust for potential confounders.

- *Reviewer Comment*: "Avoid confusion between univariate and bivariate analysis; univariate analysis is for descriptive analysis."

  Response: Thank you for this important clarification. We have corrected any misuse of terminology, distinguishing between univariate (for descriptive statistics) and bivariate analyses, and have ensured consistent usage throughout the manuscript.

Findings

- *Reviewer Comment*: "In logistic regression, use only crude odds ratios for bivariate analysis and adjusted odds ratios for multivariate analysis."

  Response: In response to this comment, we have revised the findings section to clearly present crude odds ratios for bivariate analysis and adjusted odds ratios for multivariate logistic regression. We have removed any ambiguity in terminology related to odds ratios.

We are grateful to the reviewers for their constructive feedback, which has greatly strengthened the rigor and clarity of our study. We believe the revised manuscript now provides a clearer, more accurate presentation of our methods and findings.

Thank you for considering our responses and revisions. We look forward to your further feedback.

Sincerely,

Musonda Makasa

***Corresponding author**
