# Supplementary material for: Predictors of perinatal mortality in the seven major hospitals of Lusaka Zambia: A case control study
Source: PLOS Glob Public Health. 2025 Aug 20;5(8):e0003326. doi: 10.1371/journal.pgph.0003326 (PMC12367193; doi:10.1371/journal.pgph.0003326)
Supplement: Attachment — Submitted filename: Response to Reviewers (2).pdf [file pgph.0003326.s012.pdf]

Dr Musonda Makasa

P.O Box 310129

Lusaka

April 20, 2025

PLOS Global Public Health Staff
globalpubhealth@plos.org

Dear Editor,

We would like to express our sincere appreciation to you and the reviewers for the thoughtful and constructive feedback on our manuscript titled: "**Predictors of Perinatal Mortality in Lusaka, Zambia: A Multi-Center Case-Control Study**" Manuscript ID: PGPH-D-24-01110R5

We are grateful for the opportunity to revise and improve our manuscript. We have carefully addressed all comments provided by the reviewers, and we believe the revised version of the manuscript has been significantly strengthened as a result.

Below, we provide a point-by-point response to each reviewer's comments, indicating how we have incorporated their suggestions into the revised manuscript. All changes have been tracked in the revised document.

Reviewer #6, #9

Comment: All comments were addressed.

Response: We thank Reviewer #6 for confirming that all comments have been satisfactorily addressed. No further action required.

Reviewer #7

General Comment:
We are grateful for the supportive and encouraging remarks. The reviewer recognized the study as well-designed, with compelling and impactful results. Below are responses to the specific recommendations:

Methods

1. **Birth estimates in Lusaka/hospitals:**
   Response: This study ethical clearance and approval boundaries were strictly on the seven major hospitals of Lusaka. These facilities are anchor the rest of the lower level facilities. Obtaining information on the total estimates would require another ethical clearance and approval tier. Obtaining this information from the Ministry of Health was therefore futile

as they cited that the information requested was beyond the scope and mandate of this investigation.

2. **Clarify reason for 5 excluded participants:**
   Response: We have updated the flow diagram and Methods section to include that 5 participants were withdrawn due to emotional distress.
3. **Clarify 7-day follow-up of controls:**
   Response: We have clarified in the Methods section that controls were followed up before discharge, and in rare cases during postnatal clinic visits within 7 days.

Stylistic Suggestions for Introduction and Methods

- **Reorganization and flow of introduction:**
  Response: The introduction has been restructured per the reviewer's suggestions. The background now opens with key statistics on perinatal mortality, followed by definitions, disparities across regions, and Zambia-specific context. The literature gap and study aim have also been made more prominent.
- **Sentence clarity and paragraph organization:**
  Response: We have revised, condensed, and reorganized several sentences and paragraphs for improved clarity and logical flow, as advised.
- **Revised MSB definition and deletion from discussion:**
  Response: We have clarified the definition of macerated stillbirth using "more than 8 hours prior to the onset of labor" and deleted the redundant sentence in the discussion.
- **Improved wording in Methods:**
  Response: The sentence in the Methods section has been revised to: *"Participants were prospectively recruited as cases occurred, with matched controls, and informed written consent was obtained from all participants."*

Copy-editing and Minor Corrections

All grammatical and stylistic corrections suggested—such as punctuation in the abstract, paragraph breaks in the introduction, and sentence refinement throughout—have been addressed. We have also standardized the use of "PMR" after its first full mention.

Reviewer #10

1. **Citations for statistics in the Introduction:**
   Response: We have added references to support all cited statistics, including the 2.6 million perinatal deaths figure.
2. **Updated references for maternal mortality and stillbirths:**
   Response: We have replaced older references with more recent data from WHO (2023) and the UN IGME 2023 Child Mortality Report. We will consider updating to the 2025 estimates if publication timing permits.
3. **Clarify maternal/perinatal death linkage:**
   Response: We have deleted the sentence citing references 10–12 and strengthened the paragraph using reference 13.

4. **Exclusion criteria – non-consent:**
   Response: We have explicitly stated in the Methods section that participants who did not provide consent were excluded.
5. **Clarify inclusion of known risk factors not discussed:**
   Response: We clarified that some known predictors (e.g., congenital anomalies, syphilis) were not analyzed due to data limitations. This has also been noted in the limitations section.

Reviewer #11

1. **Missing data and bias mitigation:**
   Response: We have elaborated in the Methods section on how missing data were handled and what strategies were used to minimize information bias (e.g., use of trained data collectors, real-time data checks).
2. **Variable selection in regression model:**
   Response: We have expanded the Data Analysis section to explain the rationale for selecting specific predictor variables based on prior literature and clinical relevance.
3. **Policy implications:**
   Response: A summary of policy implications and suggested interventions has been added to the Recommendations section to guide future programming.

Reviewer #12

Thank you for your supportive and constructive feedback. The following minor revisions have been made as suggested:

- **Abstract:** Revised the sentence for clarity: *"...compared to early booking."*
- **Introduction:** Revised to "...have exacerbated the issue."
- **Methods:** Revised for clarity: *"...controls were followed up before discharge."*
- **Results:** Revised sentence: *"...two times more likely..."*
- **Recommendations:** Spelled out "CEmONC" to ensure clarity for all readers.

We trust that the revised manuscript now fully addresses the concerns and suggestions raised by the reviewers. We appreciate your time and consideration and hope the revised version meets the standards for publication.

Sincerely,

Musonda Makasa

***Corresponding author**

On behalf of all co-authors
