# Supplementary material for: Predictors of perinatal mortality in the seven major hospitals of Lusaka Zambia: A case control study
Source: PLOS Glob Public Health. 2025 Aug 20;5(8):e0003326. doi: 10.1371/journal.pgph.0003326 (PMC12367193; doi:10.1371/journal.pgph.0003326)
Supplement: Attachment — Submitted filename: PGPH-D-24-01110_R6_review.pdf [file pgph.0003326.s013.pdf]

PLOS Global Public Health

Predictors of perinatal mortality in the seven major hospitals of Lusaka Zambia: A Case Control Study

--Manuscript Draft--

Manuscript Number:	PGPH-D-24-01110R6
Article Type:	Research Article
Full Title:	Predictors of perinatal mortality in the seven major hospitals of Lusaka Zambia: A Case Control Study
Short Title:	Predictors of perinatal mortality in Lusaka, Zambia
Corresponding Author:	Musonda Makasa, MBBS, MMed Obgyn, MBA, MPH, FCOG (ECSA)<br>UTH: University of Zambia University Teaching Hospital<br>Lusaka, Lusaka ZAMBIA
Order of Authors:	Musonda Makasa, MBBS, MMed Obgyn, MBA, MPH, FCOG (ECSA)
	Patrick Kaonga
	Choolwe Jacobs
	Mpundu Makasa
	Bellington Vwalika
Keywords:	perinatal; proportions; predictors; mortality
Abstract:	Background<br><br>Over 2.6 million babies are lost later in pregnancy, during labour, and or in the first week of life. Global perinatal mortality reduced from 5.7 million since 2000 to 4.1 million in 2015. High-income countries account for 45% of this data. The rest are in low-income countries, 77% of which are in sub-Saharan Africa. Perinatal mortality rates for sub-Saharan Africa and Zambia are 42.95 and 33/1000 live births, respectively. The aim of this study was to determine the predictors of perinatal mortality at the seven major hospitals of Lusaka, Zambia.<br><br>Methods<br><br>this was a multi-centre unmatched case control study from September 2023 to January 2024. Cases included perinatal death (>24 weeks gestation or >500g stillborn, and death of neonate within seven days of life) and controls were live births. Stepwise multivariate logistic regression analysis determined predictors using adjusted odd ratios and p-values.<br><br>Results<br><br>The study had 630 participants, 210 cases and 420 controls were analysed: ratio 1:2. Antenatal care booking after 12 weeks gestation had almost three times odds of experiencing perinatal (AOR 2.909, 95% CI: 1.97-4.296), p <0.001 compared to those who booked early. Walking as means of reaching healthcare facility had over three odds perinatal mortality (AOR3.482, 95% CI: 1.87-6.49) than personal transport users. Anaemia during pregnancy had over three times risk of perinatal death (AOR 3.581, 95% CI: 1.72-7.44) than those without it. History of loss of baby before birth had five fold odds to experience perinatal mortality than to those who had not (AOR 5.047, 95% CI: 2.99-8.51).<br><br>Conclusion<br><br>This study revealed that late antenatal care booking, walking, as means of transport to access health facility, anaemia in pregnancy, and previous history of loss of baby before birth perinatal death were the main predictors with statistical significance of perinatal death experience.

Powered by Editorial Manager® and ProduXion Manager® from Aries Systems Corporation

Additional Information:	
**Question**	**Response**
**Financial Disclosure**<br><br>Enter a financial disclosure statement that describes the sources of funding for the work included in this submission and the role the funder(s) played. This includes grants and any commercial funding of the work or authors.<br><br>This statement will be typeset if the manuscript is accepted for publication.<br><br>*Please review the submission guidelines and the instructions link below for detailed requirements and guidance.*	We hereby declare that this project has not received any form of commercial funding, financial support, or grant from any external funding agencies, private sector entities, or donor organizations. The work was conducted independently without any financial influence or sponsorship that could have affected the study design, data collection, analysis, interpretation, or reporting of the findings.
**Competing Interests**<br><br>On behalf of all authors, disclose any competing interests that could be perceived to bias this work.<br><br>This statement will be typeset if the manuscript is accepted for publication.<br><br>*Please review the instructions link below and PLOS Global Public Health's competing interests policy to determine what information must be disclosed at submission.*	On behalf of all the authors, I wish to formally declare that there are no actual or perceived conflicts of interest that could have influenced or biased any aspect of this work. All authors have contributed to the study with integrity and transparency, and no personal, professional, or financial relationships exist that could be viewed as compromising the objectivity or validity of the research presented.
**Data Availability**<br><br>Before publication, Authors are required to make fully available and without restriction all data underlying their findings. Please see our PLOS Data Policy page for detailed information on this policy.<br><br>A **Data Availability Statement**, detailing	"There are ethical restrictions which prevent the public sharing of minimal data for this study, because the data contains potentially identifiable patient information. Data are available upon request from the University of Zambia School of Public Health representative, Joseph Mumba Zulu, Professor of Community Health, via email:josephmumbazulu@gmail.com for researchers who meet the criteria for access to confidential data."

Powered by Editorial Manager® and ProduXion Manager® from Aries Systems Corporation

where the data can be accessed, is required at first submission. Insert your Data Availability Statement in the box below.<br><br>Please see the data reporting section of our submission guidelines for instructions on what you need to include in your Data Availability Statement.<br><br>This statement will be typeset if the manuscript is accepted for publication.<br><br>*PLOS allows rare exemptions to address legal and ethical concerns. If you have legal or ethical restrictions, please use the box below to detail these in full sentences for the Journal team to consider.*	

Powered by Editorial Manager® and ProduXion Manager® from Aries Systems Corporation

Manuscript Click here to access/download;Manuscript;Manuscript.pdf

Predictors of perinatal mortality in the seven major hospitals of Lusaka Zambia: A Case Control Study

Makasa Musonda*[1, 2, 4], Kaonga Patrick[1], Jacobs Choolwe[1], Makasa Mpundu[3], Vwalika Bellingtone[4]

[1]University of Zambia School of Public Health, Department of Epidemiology and Biostatistics, Lusaka, Zambia

[2]University Teaching Hospital, Women and Newborn Hospital, Lusaka, Zambia

[3]University of Zambia School of Public Health Department of Community and Family Medicine, Lusaka, Zambia

[4]University of Zambia School of Medicine Department of Obstetrics and Gynaecology, Lusaka, Zambia

Authors

Musonda Makasa – *Corresponding author

Email: mcmakasa@gmail.com

Patrick Kaonga

Email: patrickkaonga@gmail.com

Choolwe Jacobs

Email: choolwe2003@yahoo.com

Mpundu Makasa

Email: cmakasa@yahoo.com

Bellington Vwalika

Email: vwalikab@gmail.com

Abstract

Background: Over 2.6 million babies globally are lost before birth or in the first week of life due to largely preventable causes. Global perinatal mortality reduced from 5.7 million in 2000 to 4.1 million by end of 2015, of which 45% were recorded in high-income countries and the rest are in low-income countries. Perinatal mortality rates for sub-Saharan Africa and Zambia are at 37.4 and 33/1000 live births, respectively. The aim of this study was to determine the predictors of perinatal mortality at the seven major hospitals of Lusaka, Zambia. **Methods:** This was a multi-centre unmatched case control study from September 2023 to January 2024. Cases included perinatal death (>24 weeks gestation or >500g stillborn and death of neonate within seven days of life) and controls were live births. Stepwise multivariate logistic regression analysis with Stata version 14 determined predictors using adjusted odd ratios and p-values. **Results:** This study included 630 participants, with 210 cases and 420 controls analysed in a 1:2 ratio. Antenatal care booking after 12 weeks gestation had almost three times odds of experiencing perinatal mortality (AOR 2.91, 95% CI: 1.97-4.29), p<0.001) compared to early booking (<12 weeks). Walking as a means of reaching the healthcare facility had over three times the odds of perinatal mortality (AOR3.48, 95% CI: 1.87-6.49, p<0.012) compared to using personal transport. Anaemia in pregnancy carried a three-and-a-half-fold increased ~~the~~ risk of perinatal death (AOR 3.58, 95% CI: 1.72-7.44, p<0.001) compared to mothers without anaemia. History of previous pregnancy loss was associated with a five-fold increased risk of perinatal death (AOR 5.05, 95% CI: 2.99-8.51, p<0.001) compared to those without such a history. **Conclusion:** This study revealed that late antenatal care booking, walking as means of transport to access health facility, anaemia in pregnancy, and previous history of loss of baby before birth ~~perinatal death~~ were the main predictors with statistical significance of perinatal death experience

Keywords: Perinatal Mortality, Predictors, Antenatal Care, Anaemia, Case-Control Study

Introduction

Perinatal mortality remains a significant global health challenge particularly in low-income settings (1). Globally, in 2015, an estimated 2.6 million babies (uncertainty range: 2.4 - 3.0 million) were lost during pregnancy, with an estimated 1.3 million of these deaths occurring intrapartum (death during labour) (2). Efforts to reduce perinatal deaths in line with the World Health Organization (WHO) Sustainable Development Goals (SDG) target has yielded limited successes, particularly in sub-Saharan Africa (SSA) (3). Perinatal mortality refers to the loss of a foetus after 22 weeks of gestation, or with a birthweight ≥500g; as well as the death of a newborn within the first 7 days of life. Perinatal mortality comprises of stillbirths and early neonatal deaths. Early neonatal mortality specifically refers to the death of a newborn baby within the first 7 days of life (4). Stillbirth on the other hand, refers to intrauterine death occurring either before the onset of labour or during labour (5). Stillbirths are classified into two types: fresh stillbirths and macerated stillbirths. A fresh stillbirth (FSB) shows no signs of maceration (skin changes) at the time of delivery and is presumed to have occurred recently, typically intrapartum. In contrast, a macerated stillbirth (MSB) exhibits signs of maceration - such as skin discolouration, peeling, and soft tissue breakdown – an indication death occurred more than 6 - 8 hours prior to delivery (2, 5, 6).

Efforts to reduce perinatal deaths in line with the World Health Organization (WHO) Sustainable Development Goals (SDG) target has yielded limited successes, particularly in sub-Saharan Africa (SSA). SDG number 3.2 aims to end preventable newborn deaths by reducing neonatal mortality rate to at least 12 per 1000 live births by 2030 (1). The global perinatal mortality reduced from 5.7 million since the launch of the Millennium Development Goals (MDGs) in 2000 to 4.1 million by the time of the launch of the SDGs (7). Similarly, the global stillbirth rate also declined from 24.7 in 2000 to 18.4 per 1000 live births in 2015 (3). WHO Neonatal and Perinatal Mortality Report, 2000 indicated 47 per

1000 live births global rate of perinatal mortality and 62 per 1000 for Africa (4).

One contributing factor to the significant disparity in PMR between High-income countries (HIC) and low- and middle-income countries (LMIC) is that, until recently, most stillbirths were not included in the MDGs and were also excluded from global data tracking by either the United Nations (UN) or the Global Burden of Disease (GBD) (8). This exclusion, combined with limited social recognition, investment, and programmatic action, further exacerbated the issue (3). Additionally, in 2017, the Maternal Mortality Estimation Interagency Group (MMEIG) reported 295,000 maternal deaths worldwide, with approximately 196,000 (66.44%) occurring in SSA (9). In low-income settings, over a third of these maternal deaths and half of the stillbirths occur before or during childbirth (2, 10). It has also been reported that nearly two thirds of the causes of maternal death also contribute to perinatal deaths (11). These advancements have primarily been realized in HIC, which account for only approximately 45% of global perinatal mortality records (12). For example, in Hong Kong, the rate of perinatal mortality is 3.4 per 1000 live births and 5.54 in the United States of America (17, 18) (13, 14). In stark contrast, SSA has remained the highest Perinatal Mortality Rate (PMR) (37.31 per 1000 live births) the highest reported in Cote D'Ivoire 68.1 per 1000 live births according to a recent system review and meta-analysis (15). In southern Africa, the PMR is approximately 30.3 per 1000 live births (16, 17). Zambia is not immune to this struggle and continues to face challenges with high perinatal mortality, recording a PMR of 33 per 1000 live births, ~~according to the latest demographic health survey at the time this study was conducted~~ (18). This rate surpasses the regional average and falls significantly short of ~~short of~~ the target set by the SDG and Zambia's Vision 2030 (19).

This study aimed to assess the predictors of perinatal death across all the major hospitals of Lusaka Urban District – Zambia's most populous district and the one with the highest birth rate. In addition,

the research sought to generate evidence to inform targeted programmatic interventions and health systems strategies aimed at reducing the PMR within this high-burden setting.

General Objective

The main objective of this study was to investigate the predictors of perinatal mortality at the seven main hospitals of Lusaka, Zambia

Specific Objectives

1. To determine the social and demographic predictors of perinatal mortality at the seven main hospitals of Lusaka, Zambia
2. To determine the maternal and foetal predictors of perinatal mortality at the seven main hospitals of Lusaka, Zambia

Materials and Methods

This was a multicentre study conducted at the seven major hospitals in Lusaka urban district, Zambia. Data collection commenced on the 9th of September in 2023 and concluded on the 31st of January 2024. Participants were prospectively recruited as cases occurred with controls. Eligible participants who were agreeable to participate after being provided with detailed information via a participant information sheet were then recruited after obtaining informed written consent. The participants included mothers who experienced stillbirths or early neonatal deaths (cases) and those with live births (controls), all of whom were followed up before discharge. Data was collected using an interviewer-structured questionnaire. The hospitals involved all the major hospitals of Lusaka which include: two tertiary facilities - Women and Newborn and Levy Mwanawasa University Teaching Hospitals - and five first level hospitals: Chawama, Chilenje, Chipata, Kanyama, and Matero first level hospitals. According to the Census Report, 2022 the Lusaka is the most populous city in Zambia, with a female

population of 1,590,922 (20).

Study design

This was an unmatched prospective case-control study with a 1:2 ratio, aimed at determining the predictors of perinatal mortality at the selected study site. Cases were defined as stillbirth after 24 weeks of gestation; ≥500g where gestational age was not clear; and early neonatal deaths within seven days of life from the study sites. For each case, two controls were selected through systematic random sampling, involving women who had live births immediately before or after occurrence of a case. Controls were recruited from the same facility where the cases occurred, and for early neonatal deaths, controls were mothers who delivered within 24hrs of case's birth.

A midwife from each labour and postnatal ward at each facility was trained and enrolled as data a collector. Data collectors provided participants with an information sheet and obtained informed consent. In rare cases where translation was necessary, data collectors translated the information into the appropriate language that participants understood and were more conversant with. Data collection for postnatal mothers occurred whenever a case was identified (within the early neonatal period) and controls were subsequently recruited. In rare cases, particularly following an early neonatal death, controls were followed up before discharge or during clinic visits. These controls were mothers with live babies who delivered within 24 hours of a recruited case, ensuring their recruitment occurred around the same time as the case. Data were collected by reviewing files followed by interviewer administered collection of data using a standardized structured questionnaire on a google sheet that was managed by the Principal Investigator (PI). The PI monitored the Google data collection sheet daily.

Study population

Study population comprised women aged 18 years and older who sought and received maternal and/or

childbirth services at the study sites and met the inclusion criteria. Refer to the following: figure 1.0 flowchart illustrates the source of the sample, eligibility screening process and how the final sample was arrived at.

Figure 1.0

Here

Inclusion criteria

Cases included stillbirths from mothers aged above 18 years, with a gestational age above 24 weeks. In the absence of gestational age, a birthweight of ≥500g was considered eligible for this study.

Exclusion criteria

Women were excluded if they withdrew from the study, were under the age of 18, had a pregnancy of less than 24 weeks of gestation, or in the absence of gestational age, had a birthweight of less than 500g. Women were excluded if they were unavailable during the study period, were unable to participate due to ill health, or were too emotionally distressed to provide reliable information, even after reviewing the participant information sheet. Additionally, women who did not consent to participate were excluded.

Sample size calculation

The sample size for the study was calculated based on the assumptions that two controls per care ($r = 2$), with 80% power of the study (type II error 20%, i.e. $\beta = 0.2$), confidence interval of 95% with 5% type I error (i.e. $\alpha = 0.05$). With an assumed odds ratio of 2.5 for differences based on findings in previous similar studies. They reported for example associated potential risk factors included extremes of birthweight, post term delivery, infections, haemorrhage and previous history or perinatal mortality (21, 22). And using Zambia's PMR of 33 per 1000 live births (23), the following formula by Charan and Biswas (24) was used:

$$n = \frac{r+1}{r}\left[\frac{p^*(1-p^*)(Z_\beta + Z\alpha_{/2})^2}{(P_1 - P_2)^2}\right]$$

Where n = is the sample size, p*average proportion exposed which was given by the sum of proportion exposed cases (P_1) and proportion of control P_2 divided by 2 (i.e. $= \frac{P_1+P_2}{2}$), Z_β is the standard normal value for power (for power of 80%, Z_β = 0.84), $Z\alpha_{/2}$ is the standard normal value for the level of significance which is usually given as α=0.05 and its value is 1.96, and r is the ratio of controls to cases, in our case the ratio is 2 to 1 which means our r = 2; To find P_2, we use the formula:

$P_2 = \frac{OR \times P_1}{P_1(OR-1)+1}$, where OR is the odds ratio and P_1 is the proportion with a specific risk factor in the control participants. Assuming OR = 3.2 based on pooled analysis with strong association of perinatal mortality due to lack of antenatal care (25): P_1=0.033 in the formula above we get the value of P_2 as 0.09845

$$P^* = \frac{0.033+0.09845}{2} = \frac{0.13145}{2} = 0.0657$$

Replacing the above information in the prescribed formula above the following was the targeted sample size:

$$n = \frac{2+1}{2}\left[\frac{0.0657(1-0.0657)(0.84+1.96)^2}{(0.09845-0.033)^2}\right]$$

$$n = 168.4874$$

$$n = 169$$

Therefore, the calculated sample size was 169 cases and 338 using the 1:2 ratio, which gave 507. After factoring in 10% additional in case of participant fall out, the total sample size calculated was 558.

Sampling technique

To determine the sample size for each facility, using Probability Proportional to Size (PPS) sampling, we accounted for the delivery volume at each facility to ensure that larger facilities, which manage more deliveries, had a proportionally higher chance of selection. Hence, the following was applied in our study:

1. **To establishing facility delivery size**: We first reviewed delivery records from each facility covering January to December 2023 to estimate the delivery size at each one. This information served as the basis for determining the relative size of each facility.

2. **To ensure proportional allocation**: Facilities with higher delivery numbers were assigned a greater probability of selection compared to those with fewer deliveries. This allocation ensured that our sample would reflect the distribution of delivery cases across facilities, avoiding under-representation from larger facilities.

3. **To sample participants within facilities**: Once the facilities were selected with PPS, we then identified participants within each facility for inclusion in the study. This selection process within facilities allowed us to capture representative samples proportionate to the overall patient volume, aligning with the facility's estimated annual deliveries.

By using PPS sampling, we ensured a sample distribution that mirrors the scale of service utilization across facilities, thereby enhancing the representativeness and reliability of our findings.

A total of 635 participants were initially enrolled across all study sites. However, only 630 participants were included in the final analysis, as the remainder did not meet the inclusion criteria because they exceeded the early neonatal period. Data collection was through an interviewer administered questionnaire using a password controlled google sheet. Due to rarity of the outcome, purposeful sampling was used for the cases that met the inclusion criteria. For each case enrolled, two controls were randomly selected within the same 24-hour shift in which the case occurred.

Study variables

The outcome variables include macerated (a fresh stillbirth with features of maceration), fresh stillbirths (without features of maceration), and early neonatal death (occurred within 7 days of life). Distal level variables included age, marital status, parity, gravida, weight, BMI, employment status, educational level, distance of place of residence to health facility, and mode of transport. Intermediate level variables were antenatal booking data, number of antenatal contacts, medical history, history of pregnancy loss beyond viability, history of abortion, malaria during pregnancy, Human Immunodeficiency Virus (HIV) status, tuberculosis during pregnancy, syphilis, alcohol intake during pregnancy, illicit drugs, smoking, folic and ferrous sulphate use during pregnancy, malaria treatment during pregnancy, and sickle cell anaemia. Rhesus factor, antepartum haemorrhage, preeclampsia, and eclampsia, preterm delivery, history of low birth weight, and inter-pregnancy interval were the rest. While proximal level variables included gestational age at the time of birth, outcome, Apgar score, time lag to Neonatal Intensive Care Unit (NICU), age of early neonatal at the time of demise, sex, gross congenital anomalies, congenital syphilis, prematurity, low birthweight, and cause of death. Data collection tool

A standard researcher administered questionnaire was set up using the google sheet platform with protected access to the selected data collectors only. The PI exclusively handled daily monitoring in real-time. After completion of data collection, the data set was downloaded to an excel format for screening and cleaning and then coded to construct a do file for execution in Stata version 14.

Data analysis

Descriptive statistical analyses were conducted to summarize the proportions and frequencies of cases and controls. A bivariate analysis using Chi-square test was performed to identify the factors associated with perinatal mortality. All factors found to be associated with perinatal mortality in the bivariate

analysis (p-value <0.05) at 95% confidence interval (95% CI) were then included in multivariable logistic regression model to adjust for potential confounders. Sequential elimination of predictors was employed to assess the strength and significance of the variables associated with perinatal deaths, followed by the discrimination and calibration. Continuous variables such as [illegible]e, weight, parity, and gravidity were categorized based on clinical reasoning before being introduced into the model.

The ability of the model to correct risk was assessed using the Hosmer and Lemeshow goodness of fit test. A non-significant p-value ($p>0.05$) from this test indicated a good fit. Model stability was further evaluated by the measurement of the discrimination between participants with and without perinatal deaths using the area under the receiver operating characteristic (ROC) curve, with an acceptable values et at 0.7 (26). The study followed the Strengthening the Reporting of Observational Studies in Epidemiology (STROBE) guidelines (27).

Ethical considerations

Ethical approval for this study was obtained from the University of Zambia Biomedical Research Ethics Committee (UNZABREC), reference number 3712-2023. Prior to this, clearance was secured from the National Health Research Authority (NHRA), under reference number NHRA 000012/16/03/2023. All procedures adhered to the ethical guidelines for research involving human participants, including informed consent. Participants were thoroughly briefed about the study's purposed, procedures, potential risks, and benefits, and written informed consent obtained before enrolment.

Confidentiality and privacy were strictly maintained throughout the study. All personal identifying information was anonymized, and the data collected was stored on password-protected devices accessible only to the principal investigator (PI) and designated research team members. To ensure data security, encrypted backups were created, and physical access to data storage location was restricted.

Participants were also informed of their right to withdraw from the study at any point without any consequences. The study adhered to national and international research ethics standards.

Results

Description of study participants

This study comprised a total of a total of 630 study respondents. The total perinatal deaths 210 (cases) and 420 with live births (controls) made the ratio of cases to controls 1:2. The following figure 2.0 hereafter shows the perinatal deaths distributi[illegible]

Figure 2.0

~~Here~~

Figure 2.0 is a summary illustrating the frequency distribution of the Fresh Stillbirths (FSB), Macerated Stillbirth (MSB), and Early Neonatal Death (ENND) for this study. The proportion of the perinatal outcomes was 118 (56%) for ENNDs, 56 (27 %) MSBs, and 36 (17%), FSBs respectively.

Participants' socio-demographic characteristics

From the social-demographic attributes (distal level variables) of the respondents sampled majority of the respondents were between the age of 20-34 years 401 (63.7%). 19 years old and under were 90 (14.3%) and 97 (15.4%) were 35 years of age and above. Mean age for the study population [illegible]as 27. Of the respondents 478 (75.9%) were Married and 143 (22.7%) were not married. A total of 487 (75.9%) had a parity between 1 to 4, while 81 (12.9%) were grandmultipara. Additionally, 445 (72.4%) were not employed and 170 (27.6%) employed. For education, most of the respondents had only attained secondary level of education 331 (52.5%), primary 187 (29.7%), and 95 (15.1%) for tertiary. Most of the participants used public transport 415 (65.9%) while only 98 (15.6%) had personal transport and the others 106 (16.8%) on foot.

Results of predictors of perinatal mortality from bivariate analysis

Bivariate analysis using Chi-Square Test revealed several variables associated with perinatal mortality. The outcomes outlined in the table below revealed that the level of education of the mother had a significant association with perinatal mortality ($p<0.017$). The form of transport used when going to the hospital (on foot) also had association with perinatal mortality ($p<0.001$). Antenatal Care (ANC) booking (first contact with healthcare provider for ANC) beyond 12 weeks of gestation was statistically significant with $p<0.001$. Women who had prior history of perinatal death also had a statistically significant p-value ($p<0.001$). Abortion and Anaemia during pregnancy were found with $p <0.018$ and $p<0.003$ respectively. The rest of the independent variables had no statistical significance.

Table 1.0 Summary of the maternal socio-demographic characteristics and pregnancy factors associated perinatal mortality

Predictors	Cases (n) 210 (%)	Controls (n) 420 (%)	Total (n) 630 (%)	χ^2 p-value
**Distal level variables**				
**Age**				
≤ 19	30 (14.3)	60 (14.3)	90 (14.3)	
20-34	138 (65.7)	263 (62.6)	401 (63.7)	0.466
> 3[illegible]	27 (12.9)	70 (16.7)	97 (15.4)	
Missing	15 (7.1)	27 (6.4)	42 (6.7)	
**Marital status**				
Not married	50 (23.8)	93 (22.1)	143 (22.7)	0.741
Married	160 (76.2)	318 (75.7)	478 (75.9)	
Missing	0 (0.0)	9 (2.1)	9 (1.4)	
**Parity**				
1 – 4	170 (81.0)	309 (73.6)	487 (77.3)	0.702
> 5	36 (17.1)	62 (14.8)	81 (12.9)	
Missing	4 (1.9)	49 (11.7)	62 (9.8)	
**Employment Status**				
Not employment	151 (71.9)	294 (70.0)	445 (72.4)	0.610
Employed	54 (25.7)	116 (27.6)	170 (27.6)	
Missing	5 (2.4)	10 (2.4)	15 (2.4)	
**Educational level**				
Primary	65 (31.0)	122 (29.0)	187 (29.7)	
Secondary	121 (57.6)	210 (50.0)	331 (52.5)	**0.017***
Tertiary	20 (9.6)	75 (17.9)	95 (15.1)	
Missing	4 (1.9)	13 (3.1)	17 (2.7)	

**Intermediate level variables**				
**Mode of transport**				
Foot	50 (23.8)	56 (13.3)	106 (16.8)	**0.001***
Public transport	139 (66.2)	276 (65.7)	415 (65.9)	
Personal	20 (9.5)	78 (18.6)	98 (15.6)	
Missing	1 (0.5)	10 (2.4)	11 (1.7)	
**ANC Booking**				
< 12 weeks	100 (47.6)	83 (19.8)	183 (29.0)	
> 12 weeks	106 (50.5)	323 (76.9)	430 (68.3)	**0.001***
Missing	4 (1.9)	14 (3.3)	17 (2.7)	
**History of Stillbirth**				
Yes	64 (30.5)	32 (7.6)	96 (15.2)	**0.001***
No	143 (68.1)	377 (89.8)	520 (82.5)	
Missing	3 (1.4)	11 (2.6)	14 (2.2)	
**History of Abortion**				
Yes	10 (4.8)	7 (1.7)	17 (2.7)	**0.018***
No	181 (86.2)	393 (93.6)	574 (91.1)	
Missing	19 (9.0)	20 (4.8)	39 (6.2)	
**Proximal level variables**				
**Malaria during pregnancy**				
Yes	21 (10.0)	26 (6.2)	47 (7.5)	0.080
No	181 (86.2)	384 (91.4)	565 (89.7)	
Missing	8 (3.8)	10 (2.4)	18 (2.9)	
**HIV Status**				
Positive	30 (14.3)	59 (14.0)	89 (14.1)	0.898
Negative	173 (82.4)	351 (83.6)	524 (83.2)	
Missing	7 (3.3)	10 (2.4)	17 (2.7)	
**Tuberculosis during pregnancy**				
Yes	2 (1.0)	1 (0.2)	3 (0.5)	0.227
No	201 (95.7)	397 (94.5)	598 (94.9)	
Missing	7 (3.3)	22 (5.2)	29 (4.6)	
**Alcohol**				
Yes	14 (6.7)	42 (10.0)	56 (8.9)	0.155
No	193 (91.9)	368 (87.6)	561 (89.0)	
Missing	3 (1.4)	10 2.4)	13 (2.1)	
**Smoking**				
Yes	4 (1.9)	2 (0.5)	6 (1.0)	0.087
No	204 (97.1)	405 (96.4)	609 (96.7)	
Missing	2 (1.0)	13 (3.1)	15 (2.4)	
**Folate and Ferrous sulphate supplementation**				
Yes	197 (93.8)	396 (94.3)	593 (94.1)	0.307

No	10 (4.8)	13 (3.1)	23 (3.7)	
Missing	3 (1.4)	11 (2.6)	14 (2.2)	
**Anaemia in pregnancy**				
Yes	13 (6.2)	60 (14.3)	73 (11.6)	**0.003***
No	190 (90.5)	349 (83.1)	539 (85.6)	
Missing	7 (3.3)	11 (2.6)	18 (2.9)	
**Sickle cell anaemia**				
Yes	1 (0.5)	8 (1.9)	9 (1.0)	0.151
No	205 (97.6)	400 (95.2)	605 (96.0)	
Missing	4 (1.9)	12 (2.9)	19 (3.0)	
**Antepartum Haemorrhage**				
Yes	11 (5.2)	43 (10.2)	54 (8.6)	**0.058**
No	176 (83.8)	358 (85.2)	534 (84.8)	
Missing	23 (11.0)	19 (4.5)	42 (6.7)	
**Preeclampsia**				
Yes	20 (9.5)	47 (11.2)	67 (10.6)	0.863
No	160 (76.2)	358 (85.2)	518 (82.2)	
Missing	30 (14.3)	15 (3.6)	45 (7.1)	
**Hypertension**				
Yes	14 (6.7)	16 (3.8)	30 (4.8)	0.101
No	187 (89.0)	393 (93.6)	580 (92.1)	
Missing	9 (4.3)	11 (2.6)	20 (3.2)	
**Diabetes Mellitus**				
Yes	1 (0.5)	2 (0.5)	3 (0.5)	0.994
No	204 (97.1)	404 (96.2)	608 (96.5)	
Missing	5 (2.4)	14 (3.3)	19 (3.0)	

Unadjusted logistic regression of variables that are predictors of perinatal mortality

According to the bivariate logistic regression for predictors of perinatal death (table 2), respondents with a primary education level were almost two times likely to experience perinatal mortality compared to those the tertiary level group (COR=2.00, 95% CI: 1.12-3.56). The analysis also showed that the odds of experiencing perinatal death were over three times higher (COR=3.48, 95% CI: 1.87-6.49) for women who walked to access healthcare services and nearly two times as high (COR=1.96, 95% CI: 1.15-3.34) for those using public transport, compared to women who had personal transport.

Additionally, starting ANC after 12 weeks of gestation had over three and half odds (COR=3.68, 95%

CI: 2.56-5.30) of perinatal mortality compared to those that started earlier. Women with previous history of stillbirth were almost five times more likely to experience perinatal mortality (COR=5.27, 95 CI: 3.31-8.402) compared to those without such a history. Women with history of abortion were also three times more likely (OR=3.10, 95% CI: 1.16-8.28) to experience perinatal mortality compared to those who had no history of abortion before. This study also found that women who experienced anaemia during pregnancy had more than two and half times higher odds of perinatal mortality (COR=2.51, 95 CI: 1.34-4.69) compared to those without anaemia.

To understand the combined impact of predictors of perinatal mortality and to control for confounding, it required multivariate logistic regression analysis. Table 2.0 is the illustration of the final multivariate model that best describes predictors of perinatal mortality. Following a thorough analysis and correction for the impact of extraneous variables, four variables stood out as predictors of perinatal mortality. While educational level after multivariate logistic regression analysis did not show significance as a predictor of perinatal mortality. Mode of transport to the healthcare facility, on the other hand, showed significance as a predictor of perinatal mortality. Women who walked to the hospital had three times the odds of perinatal mortality compared to those who used personal transport (AOR 3.48, 95% CI: 1.87–6.49, $p < 0.001$). Antenatal care (ANC) booking after 12 weeks of gestation was significantly associated with an increased risk of perinatal mortality. Women who booked ANC late had nearly three times higher odds of experiencing perinatal mortality compared to those who booked early (AOR 2.91, 95% CI: 1.97–4.29, $p < 0.001$). Maternal anemia during pregnancy was associated with a three-and-a-half-fold increased risk of perinatal mortality. Women with anemia had significantly higher odds of perinatal death compared to non-anemic women (AOR 3.58, 95% CI: 1.72–7.44, $p < 0.001$). A history of previous pregnancy loss was strongly associated with increased odds of perinatal mortality. Women with a history of stillbirth had five times the odds of perinatal death compared to those without such a history (AOR 5.05, 95% CI: 2.99–8.51, $p < 0.001$). Table 2.0

presents a summarized comparison of univariable and multivariable predictors following bivariable and then multivariable logistic regression analysis.

Table 2.0: Multivariable logistic regression analysis for factors associated with perinatal mortality in the selected study sites

**Univariable logistic regression**				**Multivariable logistic regression analysis**		
**Predictor variable**	**Crude Odds Ratio (COR)**	**95%CI**	**p-value**	**Adjusted Odds Ratio (AOR)**	**95% CI**	**P-value**
**Educational level**						
Tertiary	Ref					
Primary	2.00	(1.12-3.56)	0.019	1.66	(0.83-3.31)	0.152
Secondary	2.16	(1.26-3.71)	0.005	1.87	(0.98-3.57)	**0.058**
**Mode of Transport**						
Personal	Ref					
Foot	3.48	(1.87-6.49)	<0.001	2.75	(1.23-5.39)	**0.012***
Public	1.96	(1.15-3.34)	0.013	1.70	(0.92-3.16)	0.091
**ANC Booking**						
< 12 weeks	Ref			Ref		
> 12 weeks	3.68	(2.[illegible]6-5.30)	<0.001*	2.91	(1.97-4.3)	**<0.001***
**History of Stillbirth**						
No	Ref			Ref		
Yes	5.27	(3.31-8.40)	<0.001*	5.05	(2.99-8.51)	**<0.001***
**History of abortion**						
No	Ref					
Yes	3.10	(1.16-8.28)	0.024	0.86	(0.24-3.06)	0.814
**Anaemia during pregnancy**						
No	Ref			Ref		
Yes	2.51	(1.34-4.69)	0.004	3.58	(1.72-7.44)	**0.001***

Discussion

The objective of this study was to identify predictors of perinatal mortality in Lusaka, Zambia. The selected sites are the major hospitals of Lusaka and referral hospitals that anchor all maternal and perinatal health services. Among the socio-demographic variables, educational level and mode of

transport were the only variables strongly associated with perinatal mortality. The statistically significant predictors of perinatal mortality identified in this study were mode of transport used by women when accessing healthcare, timing of antenatal care (ANC) bookings, history of abortion, stillbirth, and anaemia during pregnancy.

In clinical settings, pregnancy in women of advanced maternal age (AMA) (i.e. maternal age >35) is frequently labelled as "high-risk even in the absence of obvious risk factors. This classification has been validated by many authors from a meta-analysis on "advanced maternal age: adverse outcomes of pregnancy", where it was reported that AMA women had higher perinatal mortality and stillbirths compared to women of 20 – 34 maternal age group (28). On the contrary, in thisstudy maternal age was not found to have significant association with perinatal mortality. Educational level and mode of transport are the other socio-demographic factors that demonstrated association with our outcome of interest. However, during univariable and multivariable logistic regression analysis only the mode of transport remained statistically significant as a predictor of perinatal mortality. While education level did not show strong association with perinatal mortality in this investigation. A study on effect of maternal education and perinatal outcome done in Punjab, India reported that lower educational status was a significantly important predictor of adverse perinatal outcomes including perinatal mortality (29).

This study revealed that macerated stillbirths and early neonatal deaths (ENND) had a higher occurrence among the perinatal mortality cases, consistent with findings reported in other studies (30, 31). Another study by Shelke et.al (32) similarly reported a higher proportion of macerated stillbirths compared to fresh stillbirths, which may reflect the quality of prenatal and obstetric care, while macerated stillbirths are typically the results of intrauterine foetal death occurring more than 6 to 12 hours prior to delivery (2, 5, 33). Contributing factors to macerated stillbirths include intrauterine growth

restriction, placental lesions, and infections, though a large proportion of these deaths remain unexplained (34). In this study, these cases also remained unexplained, underscoring the need for further investigation into the underlying causes and improved data collection practices. Early neonatal deaths are often caused by complications such as low birth weight, birth asphyxia, and sepsis, which require neonatal intensive care (37,39) (35, 36). However, in this study, the causes of early neonatal mortality could not be determined because of the nature of the study design that could measure association.

Among the distal level variables none of them had statistical significance as a predictor of perinatal mortality. On the other hand, it was mainly the intermediate level variables that had significant association with perinatal mortality: mode of transport, ANC booking, and history of stillbirth. Whereas only anaemia during pregnancy was found to be a significant predictor of perinatal mortality among the proximal level variables. The mode of transport showed statistical significance as a predictor of perinatal mortality. This was because women who walked to the healthcare facility to seek medical care during pregnancy were found to be three times more likely to experience perinatal death compared to those with personal transportation. Those using public transport also had two times higher risk of perinatal mortality experience. This finding aligns with a previous study done in Zambia and Uganda, which identified a significant difference in perinatal outcomes based on motorized vs non-motorized means of transport (37). Factors contributing to this difference may include poor physical access to healthcare, lack of empowerment for decision- making, and limited health education (38). Furthermore, women who relied on non-motorized transport, such as walking to seek medical care - as was observed in this study - were more likely to access Basic Emergency Obstetric and Neonatal Care (BEmONC) facilities. In contrast, those using motorized transport who had access to Comprehensive Emergency Obstetrics and Neonatal Care (CEmONC) facilities, which provide superior care and improved maternal and perinatal outcomes, particularly when staffed with trained

personnel and equipped with essential resources (39, 40).

The timing of ANC bookings also played a significant role in perinatal outcomes. The WHO recommends that the first ANC visit should occur within the first trimester, up to 12 weeks of gestation (41). In this study, over 70% of the women booked late for ANC (> 12 weeks), and late booking was associated with a threefold increased risk of perinatal mortality. This finding corroborated other studies, which reported that delayed ANC increases the risk of complications during pregnancy and delivery, contributing to perinatal mortality (42, 43). A previous history of abortion did not emerge as a significant predictor of perinatal mortality in this study. However, a history of abortion can increase the risk of perinatal mortality compared to those without such a history. This may be attributed to a higher susceptibility to infections during subsequent pregnancies among women with history of abortion (44), which raises the likelihood of perinatal mortality (45). Conversely, previous history of stillbirth, in this study, was significantly associated with a fivefold increase in the risk of perinatal mortality. This finding aligns with results from other studies in Zambia (46) and Zimbabwe (47), which similarly reported that women with a history of stillbirth faced higher risk of perinatal mortality. This also aligns with findings from a study conducted in Ethiopia (44), which demonstrated that women with a history of stillbirth were at a higher risk of infections during subsequent pregnancies. These infections can compromise foetal health, leading to complications that significantly elevate the likelihood of perinatal mortality (45). Despite such findings from other studies linking infections to perinatal mortality, infections were not significantly associated with perinatal mortality in this study. Additionally, many stillbirths remain unexplained, largely due to the low availability of post-mortem investigations (48). This lack of clear causality makes discussing stillbirths challenging, not only for the parents but also for healthcare professionals, who often face difficulties in obtaining consent for post-mortem investigation aimed at determining the cause of death (49).

Among the medical complications, anaemia during pregnancy was the strongest predictor of perinatal mortality, with women suffering from anaemia showing a two-and-a-half-fold increased risk, compared to those without anaemia. Anaemia is closely linked to small-for-gestational-age infants and preterm birth, both of which are significant risk factors for perinatal mortality ([illegible]6). While previous studies in Zambia (21, 50) identified small-for-gestational-age infants, preterm birth, and low birth weight as high-risk factors for perinatal mortality, this study did not find these findings to have significant association with perinatal mortality.

Study Implications

Based on our study findings, socio-demographic factors like lower education level (primary and secondary) and mode of transport showed association with higher likelihood of perinatal mortality, although the latter was not statistically significant in the adjusted model. Participants with personal transport were significantly associated with lower odds of perinatal mortality even in the adjusted multivariable logistic regression model compared to those using public or walked to seek healthcare service. This may be suggestive of other factors like lower socioeconomic status and transport accessibility play a significant role in influencing pregnancy outcomes. Further interventions should focus on tackling such socio-demographic issues like improving transport accessibility and education.

The study also highlighted several other factors, such as late ANC booking, a history of stillbirth, and anaemia during pregnancy, which remained significantly associated with higher odds of perinatal mortality even after multivariable regression analysis. These findings imply that health policies should prioritize improving early ANC attendance and targeting high-risk pregnancies, particularly such as anaemia in pregnancy and history of stillbirth should be the centre theme for developing interventions for positive pregnancy outcomes. In this vein, future research should explore potential interventions to reduce the effects of these predictors and improve perinatal outcomes.

Strengths and Limitations

This study provides a comprehensive assessment of multiple predictors of perinatal mortality simultaneously, offering a nuanced understanding of the factors influencing perinatal outcomes in Lusaka district. The sample is representative of the broader population, as data were collected from all major hospitals in the city, ensuring a diverse range of cases that enhances the generalizability of the findings. This broad representation strengthens the relevance of the study to policymakers and healthcare practitioners, as the insights derived can inform targeted interventions and resource allocation in public health strategies. Furthermore, identifying significant predictors not only contributes to the existing body of literature but also equips clinicians with evidence-based knowledge to improve maternal and neonatal care practices.

One notable limitation is the incompleteness of information during data collection, primarily due to inconsistencies in medical record-keeping, and data entry errors which resulted in gaps in some variables. Therefore, some of the variables such as congenital syphilis and congenital anomalies resulted in not being included in the analysis. Additionally, accurately stating or recording the cause of death in stillbirth cases remains challenging, as investigations are not routinely conducted to determine the cause of death. These limitations highlight the need for improved data collection practices and post-mortem investigations to enhance the accuracy and reliability of future research in this area.

Conclusion

In conclusion, this study highlights several critical predictors of perinatal mortality in Lusaka, Zambia, including mode of transport, timing of antenatal care booking, previous stillbirths, and maternal anaemia. Women who walked or used public transport to access healthcare faced higher risks of perinatal mortality, underscoring the socioeconomic disparities affecting access to quality obstetric

care. The late initiation of antenatal care compromised early detection and management of complications, emphasizing the need for public health initiatives to promote timely antenatal care. Additionally, history of stillbirth significantly increased the risk of subsequent perinatal mortality, highlighting the necessity for psychological and medical support for women with previous adverse pregnancy outcomes. Maternal anaemia was also a substantial risk factor, correlating with adverse birth outcomes such as small for gestational age and preterm births. While the study did not find small for gestational age or preterm birth to be direct predictors, these findings align with existing literature. Overall, targeted interventions addressing these predictors— such as improving transportation options, enhancing antenatal care accessibility, and providing support for women with prior adverse pregnancy experiences—could significantly reduce perinatal mortality rates and improve maternal and neonatal health outcomes in Lusaka. The aim of this study was to identify the predictors of perinatal mortality in Lusaka, Zambia.

Recommendations

According to this study findings, to help to reduce the perinatal mortality in Lusaka, Zambia: there is need to improve transportation to enhance options for pregnant women especially in the underserved areas; need to ensure timely access to Comprehensive Emergency Obstetrics and Neonatal Care (CEmONC) facilities; Early ANC booking needs to be promoted through strengthening education and outreach programs to encourage women to book for ANC within the first trimester of pregnancy. In addition, to address anaemia during pregnancy routine screening and treatment need to be strengthened to reduce the associated risks to perinatal mortality. Women with previous experience of perinatal mortality need targeted support and monitoring to minimise risks in subsequent pregnancies. This support includes but not limited to enhanced quality prenatal and obstetrics care investment focusing on early detection and management of pregnancy complications. Finally, community-based health education and

empowerment programs to equip women with knowledge and resources for better pregnancy outcomes and self-autonomy in decision-making.

Acknowledgements

I would like to thank all my supervisors for the guidance provided to complete this study. I also would like to acknowledge the immense contribution the study participants for accepting to be part of the project.

Conflict of interest

We declare no conflict of interest.

Authors' contributions

MM: conceptualization of the study, study design, data collection, data cleaning, data processing and analysis, and manuscript writing. PK: supervision and methodology. CJ: supervision and contextualization of the project. MM: conceptualization and contextualization of the study. BV: overall content supervision, contextualization of the study, and methodology. All authors have read and approved the final version of the manuscript.

References

1. Organization WH. Health in 2015: from MDGs, millennium development goals to SDGs, sustainable development goals. Health in 2015: from MDGs, Millennium Development Goals to SDGs, Sustainable Development Goals2015.
2. Lawn JE, Blencowe H, Waiswa P, Amouzou A, Mathers C, Hogan D, et al. Stillbirths: rates, risk factors, and acceleration towards 2030. The Lancet. 2016;387(10018):587-603.
3. De Bernis L, Kinney MV, Stones W, ten Hoope-Bender P, Vivio D, Leisher SH, et al. Stillbirths: ending preventable deaths by 2030. The lancet. 2016;387(10019):703-16.
4. Organization WH. Neonatal and perinatal mortality: country, regional and global estimates: World Health Organization; 2006.
5. Gold KJ, Abdul-Mumin AR, Boggs ME, Opare-Addo HS, Lieberman RW. Assessment of "fresh" versus "macerated" as accurate markers of time since intrauterine fetal demise in low-income countries. Int J Gynaecol Obstet. 2014;125(3):223-7.
6. Shanklin DR. FETAL MACERATION. 2. AN ANALYSIS OF 53 HUMAN STILLBORN INFANTS. Am J Obstet Gynecol. 1964;88:224-9.
7. Wang H, Bhutta ZA, Coates MM, Coggeshall M, Dandona L, Diallo K, et al. Global, regional, national, and selected subnational levels of stillbirths, neonatal, infant, and under-5 mortality, 1980–2015: a systematic analysis for the Global Burden of Disease Study 2015. The Lancet. 2016;388(10053):1725-74.
8. Lawn JE, Blencowe H, Pattinson R, Cousens S, Kumar R, Ibiebele I, et al. Stillbirths: Where? When? Why? How to make the data count? The Lancet. 2011;377(9775):1448-63.
9. Organization WH. Trends in maternal mortality 2000 to 2017: estimates by WHO, UNICEF, UNFPA, World Bank Group and the United Nations Population Division: executive summary. 2019.
10. Say L, Chou D, Gemmill A, Tunçalp Ö, Moller A-B, Daniels J, et al. Global causes of maternal death: a WHO systematic analysis. The Lancet global health. 2014;2(6):e323-e33.
11. Berhan Y, Berhan A. Meta-analysis of selected maternal and fetal factors for perinatal mortality. Ethiopian journal of health sciences. 2014;24:55-68.
12. Blencowe H, Cousens S, Jassir FB, Say L, Chou D, Mathers C, et al. National, regional, and worldwide estimates of stillbirth rates in 2015, with trends from 2000: a systematic analysis. The Lancet Global Health. 2016;4(2):e98-e108.
13. Kumsa H, Mislu E, Yimer NB. A systematic review and meta-analysis of the globally reported International Classification of Diseases to Perinatal Mortality (ICD-PM). Frontiers in medicine. 2024;11:1434380.
14. Valenzuela CP, Gregory EC, Martin JA. Perinatal Mortality in the United States, 2020–2021: US Department of Health and Human Services, Centers for Disease Control and …; 2023.
15. Bezie MM, Asebe HA, Asnake AA, Fente BM, Negussie YM, Asmare ZA, et al. Factors associated with perinatal mortality in sub-Saharan Africa: A multilevel analysis. PloS one. 2024;19(11):e0314096.
16. Akombi BJ, Renzaho AM. Perinatal mortality in sub-Saharan Africa: a meta-analysis of demographic and health surveys. Annals of global health. 2019;85(1):106.
17. Tiruneh D, Assefa N, Mengiste B. Perinatal mortality and its determinants in Sub Saharan African countries: systematic review and meta-analysis. Maternal health, neonatology and perinatology. 2021;7:1-17.
18. Zambia Statistics Agency - ZSA, Ministry of Health - MOH, University Teaching Hospital Virology Laboratory - UTH-VL, ICF. Zambia Demographic and Health Survey 2018. Lusaka, Zambia: ZSA, MOH, UTH-VL and ICF; 2020.
19. Republic of Z. Vision 2030: A prosperous middle-income nation by 2030. Ministry of Finance and National Planning Lusaka; 2006.

20. Zambia Statistics Agency - ZSA. 2022 CENSUS OF POPULATION AND HOUSING. 2022.
21. Stringer EM, Vwalika B, Killam WP, Giganti MJ, Mbewe R, Chi BH, et al. Determinants of stillbirth in Zambia. Obstet Gynecol. 2011;117(5):1151-9.
22. Chuwa FS, Mwanamsangu AH, Brown BG, Msuya SE, Senkoro EE, Mnali OP, et al. Maternal and fetal risk factors for stillbirth in Northern Tanzania: A registry-based retrospective cohort study. PloS one. 2017;12(8):e0182250.
23. Zambia Statistics Agency ZSA, Ministry of Health MOH, University Teaching Hospital Virology Laboratory U-V, Icf. Zambia Demographic and Health Survey 2018. Lusaka, Zambia: ZSA, MOH, UTH-VL and ICF; 2020.
24. Charan J, Biswas T. How to calculate sample size for different study designs in medical research? Indian journal of psychological medicine. 2013;35(2):121-6.
25. Aragaw Y. Perinatal mortality and associated factor in Jimma university specialized hospital, South West Ethiopia. Gynecol Obstet (Sunnyvale). 2016;6(409):2161-0932.
26. Kleinbaum DG, Klein M. Survival analysis a self-learning text: Springer; 1996.
27. Von Elm E, Altman DG, Egger M, Pocock SJ, Gøtzsche PC, Vandenbroucke JP. The Strengthening the Reporting of Observational Studies in Epidemiology (STROBE) statement: guidelines for reporting observational studies. The lancet. 2007;370(9596):1453-7.
28. Pinheiro RL, Areia AL, Mota Pinto A, Donato H. Advanced maternal age: adverse outcomes of pregnancy, a meta-analysis. Acta medica portuguesa. 2019;32(3):219-26.
29. Chopra I, Juneja SK, Sharma S. Effect of maternal education on antenatal care utilization, maternal and perinatal outcome in a tertiary care hospital. Int J Reprod Contraception, Obstet Gynecol. 2018;8(1):247.
30. Abdulhameed AL, Aljammas EK, Ramzi A. Perinatal mortality types. Sulaymania, Iraq: Department of Family and Community Medicine, College of Medicine. 2016:366-72.
31. Mustufa MA, Kulsoom S, Sameen I, Moorani KN, Memon AA, Korejo R. Frequency of Stillbirths in a Tertiary Care Hospital, Karachi. Pak J Med Sci. 2016;32(1):91-4.
32. Shelke SP, Shivkumar PV, Chaurasia AK. Incidence of Stillbirth in Relation to Period of Delivery, Socioeconomic Status and Period of Gestation in a Rural Tertiary Care Hospital-MGIMS, Sewagram Within Study Period of Three Years (2013-2016). J Obstet Gynaecol India. 2022;72(Suppl 1):1-5.
33. Langley F. The perinatal postmortem examination. Journal of clinical pathology. 1971;24(2):159-69.
34. Man J, Hutchinson JC, Heazell AE, Ashworth M, Levine S, Sebire NJ. Stillbirth and intrauterine fetal death: factors affecting determination of cause of death at autopsy. Ultrasound in Obstetrics & Gynecology. 2016;48(5):566-73.
35. Gaíva MAM, Bittencourt RM, Fujimori E. Early and late neonatal death: characteristics of mothers and newborn. Revista Gaúcha de Enfermagem. 2013;34:91-7.
36. Bogale TN, Worku AG, Bikis GA, Kebede ZT. Why gone too soon? Examining social determinants of neonatal deaths in northwest Ethiopia using the three delay model approach. BMC Pediatrics. 2017;17(1):216.
37. Sacks E, Vail D, Austin-Evelyn K, Greeson D, Atuyambe LM, Macwan'gi M, et al. Factors influencing modes of transport and travel time for obstetric care: a mixed methods study in Zambia and Uganda. Health Policy and Planning. 2015;31(3):293-301.
38. Ayalew Tiruneh G, Melkamu Asaye M, Solomon AA, Tiruneh Arega D. Delays during emergency obstetric care and their determinants among mothers who gave birth in South Gondar zone hospitals, Ethiopia. A cross-sectional study design. Global Health Action. 2021;14(1):1953242.
39. Yusrawati Y, Respati T, Sekarwana N. Factors to improve the quality of comprehensive emergency obstetric and newborn care. International Journal of Public Health. 2023;12(3):1030-9.

40. Widyantari KY, Sulistyaningsih I, editors. The Effectiveness of Cemonc on Maternal Mortality: Systematic Literature Review. Proceeding International Conference; 2019.
41. Organization WH. WHO antenatal care recommendations for a positive pregnancy experience: nutritional interventions update: multiple micronutrient supplements during pregnancy. 2020.
42. Girum T. Alternative & Integrative Medicine. 2016.
43. Carroli G, Rooney C, Villar J. How effective is antenatal care in preventing maternal mortality and serious morbidity? An overview of the evidence. Paediatric and perinatal Epidemiology. 2001;15:1-42.
44. Feleke BE, Feleke TE. A longitudinal study on the effects of previous stillbirth or abortion on subsequent pregnancies and infants. European journal of public health. 2021;31(6):1237-41.
45. Suleiman MB, Mokuolu OA. Perinatal mortality in a Northwestern Nigerian city: a wake up call. Frontiers in Pediatrics. 2014;2:105.
46. Makasa M, Mutale W, Lubeya MK, Mpetemoya T, Chinayi M, Mangala B, et al. Determinants of stillbirth in the Five General Hospitals of Lusaka, Zambia: A Case-Control study. Medical Journal of Zambia. 2023;50(1):35-46.
47. Dube K, Lavender T, Blaikie K, Sutton CJ, Heazell AE, Smyth RM. Identification of factors associated with stillbirth in Zimbabwe–a cross sectional study. BMC pregnancy and childbirth. 2021;21:1-14.
48. Gardosi J, Kady SM, McGeown P, Francis A, Tonks A. Classification of stillbirth by relevant condition at death (ReCoDe): population based cohort study. Bmj. 2005;331(7525):1113-7.
49. Heazell A, McLaughlin MJ, Schmidt E, Cox P, Flenady V, Khong T, et al. A difficult conversation? The views and experiences of parents and professionals on the consent process for perinatal postmortem after stillbirth. BJOG: An international journal of obstetrics & Gynaecology. 2012;119(8):987-97.
50. Chibwesha CJ, Zanolini A, Smid M, Vwalika B, Kasaro MP, Mwanahamuntu M, et al. Predictors and outcomes of low birth weight in Lusaka, Zambia. International Journal of Gynecology & Obstetrics. 2016;134(3):309-14.

Figure

Click here to access/download;Figure;Figure 1.0 Selection of Study Participants Flow Chart.pdf

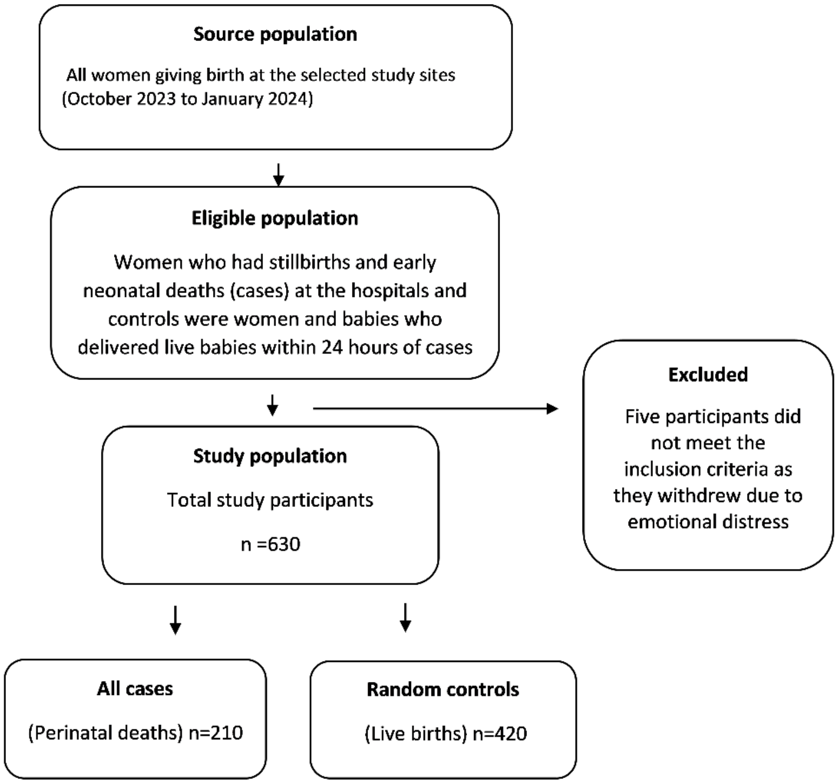

Figure 1.0 Selection of Study Participants' Flow Chart

Figure

Click here to access/download;Figure;Figure 2.0 Distribution of Perinatal Outcomes.pdf

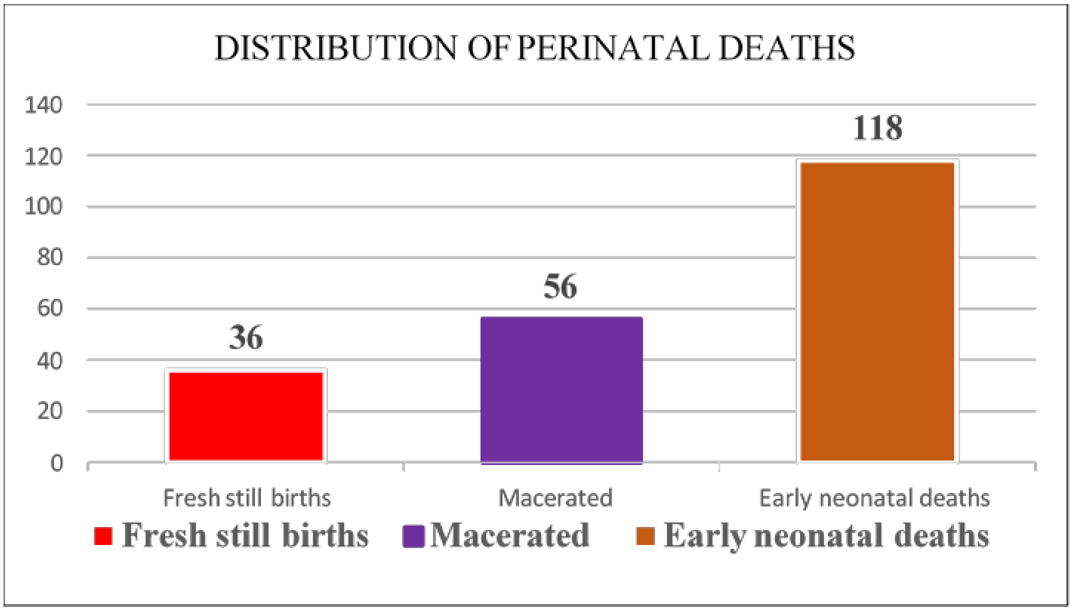

Figure 2.0 *distribution of perinatal mortality according to outcomes*

Click here to access/download
Supporting Information
PARTICIPANT INFORMATION SHEET.docx

Predictors of perinatal mortality in the seven major hospitals of Lusaka Zambia: A Case Control Study

Makasa Musonda*[1, 2, 4], Kaonga Patrick[1], Jacobs Choolwe[1], Makasa Mpundu[3], Vwalika Bellingtone[4]

[1]University of Zambia School of Public Health, Department of Epidemiology and Biostatistics, Lusaka, Zambia

[2]University Teaching Hospital, Women and Newborn Hospital, Lusaka, Zambia

[3]University of Zambia School of Public Health Department of Community and Family Medicine, Lusaka, Zambia

[4]University of Zambia School of Medicine Department of Obstetrics and Gynaecology, Lusaka, Zambia

Authors

Musonda Makasa – *Corresponding author

Email: mcmakasa@gmail.com

Patrick Kaonga

Email: patrickkaonga@gmail.com

Choolwe Jacobs

Email: choolwe2003@yahoo.com

Mpundu Makasa

Email: cmakasa@yahoo.com

Bellington Vwalika

Email: vwalikab@gmail.com

Field Code Changed
Formatted: Font color: Auto
Formatted: Font color: Auto
Field Code Changed
Formatted: Font color: Auto
Formatted: Font color: Auto
Formatted: Font color: Auto
Formatted: Font color: Auto
Field Code Changed
Field Code Changed
Formatted: Font color: Auto
Formatted: Font color: Auto
Field Code Changed
Formatted: Font color: Auto
Formatted: Font color: Auto

Abstract

Background: Over 2.6 million babies globally are lost before birth or in the first week of life due to largely preventable causes~~??~~. Global perinatal mortality reduced from 5.7 million in 2000 to 4.1 million by end of 2015, ~~45%~~ of which 45% were recorded in high-income countries and the rest are in low-income countries. Perinatal mortality rates for sub-Saharan Africa and Zambia are ~~at 37.4~~at 37.4 and 33/1000 live births, respectively. The aim of this study was to determine the predictors of perinatal mortality at the seven major hospitals of Lusaka, Zambia. **Methods:** This was a multi-centre unmatched case control study from September 2023 to January 2024. Cases included perinatal death (>24 weeks gestation or ~~r~~

>500g stillborn~~,~~ and death of neonate within seven days of life) and controls were live births. Stepwise multivariate logistic regression analysis with Stata version 14 determined predictors using adjusted odd ratios and p-values. **Results:** This study included 630 participants, with 210 cases and 420 controls analysed in a 1:2 ratio. Antenatal care booking after 12 weeks gestation had almost three times odds of experiencing perinatal mortality (AOR 2.91, 95% CI: 1.97-4.29), p

<0.001) compared to early booking (<12 weeks). Walking as a means of reaching the healthcare facility had over three times the odds of perinatal mortality (AOR3.48, 95% CI: 1.87-6.49, p<0.012) compared to using personal transport. Anaemia in pregnancy carried a three-and-a-half-fold increased the risk of perinatal death (AOR 3.58, 95% CI: 1.72-7.44, p<0.001) compared to mothers without anaemia.~~the non-anaemic?~~. History of previous pregnancy loss was associated with a five-fold increased risk of perinatal death (AOR 5.05, 95% CI: 2.99-8.51, p<0.001) compared to those without such a history. **Conclusion:** This study revealed that late antenatal care booking, walking~~,~~ as means of transport to access health facility, anaemia in pregnancy, and previous history of loss of baby before birth perinatal death were the main predictors with statistical significance of perinatal death experience.

Formatted: Indent: Left: 0", Space Before: 8.9 pt

Formatted: Indent: Left: 0", Right: 0.75", Space Before: 8.9 pt

Keywords: Perinatal Mortality, Predictors, Antenatal Care, Anaemia, Case-Control Study

Introduction

Perinatal mortality remains a significant global health challenge particularly in low-income settings (1)~~(5)~~. Globally, in 2015, ~~A~~an estimated 2.6 million babies (uncertainty range: 2.4 - 3.0 million) were~~are~~ lost~~ ann~~ during pregnancy,~~ually during pregnancy~~ with an estimated 1.3 million of these deaths occurring intrapartum (death during labour) (2)~~, labour and or in the first seven days of birth~~. Efforts to reduce perinatal deaths in line with the World Health Organization (WHO) Sustainable Development Goals (SDG) target has yielded limited successes, particularly in sub-Saharan Africa (SSA) (3). Perinatal mortality refers to the loss of a foetus after 22~~4~~ weeks of gestation, or with a birthweight

Formatted: Indent: Left: 0", Line spacing: Double

≥500g; as well as the death of a newborn within the first 7 days of life. Perinatal mortality comprises of stillbirths and early neonatal deaths. Early neonatal mortality specifically refers to the death of a newborn baby within the first 7 days of life (4)~~(1)~~. Stillbirth on the other hand, refers to~~ is~~ intrauterine death occurring either ~~that occurs intrauterine~~ before the onset of labour or during labour (5)~~(2)~~. Stillbirths are classified into two types:~~consist of~~ fresh stillbirths and macerated stillbirths. A fresh stillbirth (FSB) shows no~~does not have~~ signs of maceration (skin changes) at~~by~~ the time of delivery and is presumed to have occurred~~died more~~ recently, typically ~~during labour (~~intrapartum~~)~~. In contrast~~Whereas~~, a macerated stillbirth (MSB) exhibits~~shows~~ signs of maceration ~~-skin and soft tissue changes like~~ such as skin discolouration, peeling, and soft tissue breakdown – an indication~~which is suggestive of~~ death occurred~~ing~~ ~~well~~more than 6 - 8~~8~~ hours prior to~~before~~ delivery (2, 5, 6)~~onset of labour (2-4)~~. ~~Perinatal mortality remains a significant global health challenge particularly in low-income settings (5). An estimated 2.6 million babies are lost per year~~annually~~ during pregnancy, labour, and or in the first seven days of birth~~new borns' lives.~~

Formatted: Space Before: 15.9 pt, Line spacing: Double

Efforts to reduce perinatal deaths in line with the World Health Organization (WHO) Sustainable Development Goals (SDG) target has yielded limited successes, particularly in sub-Saharan Africa (SSA).. For instance, until Rrecently, most stillbirths were excluded from global data tracking, and the lack of social recognition, investment, and programmatic action has exacerbated the issue (6). In 2017, the Maternal Mortality Estimation Interagency Group (MMEIG) reported 295,000 maternal deaths worldwide, with approximately 196,000 (66.44%) occurring in SSA(SSA (7). In low-income settings, over a third of these maternal deaths and half of the stillbirths occur before or during childbirth (8, 9). Literature shows (10,-12) a strong link between maternal deaths and perinatal mortality because for every maternal death, there is an estimated 10 perinatal deaths. The authors also reported that nearly two thirds of causes of maternal death also contribute to perinatal deaths (13). The WHO

Formatted: Indent: Left: 0"

Commented [A1]: 66% of 295000 is 194700, you can check it please.

Formatted: Indent: Left: 0", First line: 0", Right: 0.75", Space Before: 0 pt

SDG number 3.2 aims to end preventable newborn deaths by reducing neonatal mortality rate to at least 12 per 1000 live births by 2030 (1)~~(5)~~. The global perinatal mortality reduced from 5.7 million since the launch of the Millennium Development Goals (MDGs) in 2000 to 4.1 million by the time of the launch of the SDGs (7)~~(14)~~. Similarly, the global stillbirth rate also declined from 24.7 in 2000 to 18.4 per 1000 live births in 2015 (3)~~(15)~~. ~~By the year 2000, the~~ WHO N~~n~~eonatal and P~~p~~erinatal M~~m~~ortality R~~r~~eport, 2000 indicated 47 per 1000 live births global rate of perinatal mortality and 62 per 1000 for Africa

(4)~~(1)~~.

Formatted: Indent: Left: 0", Space Before: 0 pt

One contributing factor to the significant disparity~~fference~~ in PMR between High-i~~I~~ncome c~~C~~ountries (HIC) and low- and middle-income countries (LMIC) is that, until recently, most stillbirths were not included in the MDGs and were also excluded from global data tracking by either the United Nations (UN) or the Global Burden of Disease (GBD) (8).~~,~~ This exclusion, combined with limited~~and the lack of~~ social recognition, investment, and programmatic action, further ~~haves~~ exacerbated the issue (3)~~(Organization, 2015)(6)~~. Additionally, in 2017, the Maternal Mortality Estimation Interagency Group (MMEIG) reported 295,000 maternal deaths worldwide, with approximately 196,000 (66.44%) occurring in SSA (9)~~(7)~~. In low-income settings, over a third of these maternal deaths and half of the stillbirths occur before or during childbirth ~~(8, 9)~~(2, 10). It has also been reported that nearly two thirds of the causes of maternal death also contribute to perinatal deaths (11)~~(13).~~ These advancements~~strides however,~~ have primarily~~only~~ been real~~ist~~ize~~de~~ in ~~High Income Countries (~~HIC~~)~~, which ~~that even~~ account for ~~only~~ only approximately 45% of ~~the~~ global ~~records on~~ perinatal mortality records (12)~~(16)~~. For example, in Hong Kong, the rate of perinatal mortality is 3.4 per 1000 live births and 5.54 ~~per 1000~~ in the United States of America (17, 18) (13, 14). In stark contrast, SSA has remained ~~with~~ the highest Perinatal Mortality Rate (PMR) (37.31 per 1000 live births) ~~with~~ the highest reported

Formatted: Indent: Left: 0"

Commented [A2]: 66% of 295000 is 194700, you can check it please.

in Cote D'Ivoire 68.1 per 1000 live births according to a recent system review and meta-analysis ~~(19)~~(15). In southern Africa, the PMR is approximately 30.3 per 1000 live births ~~(20,) (21)~~ (16, 17). Zambia is not immune to th~~is~~e ~~ongoing~~ struggle and continues to face challenges with high perinatal mortality, recording a PMR of 33 per 1000 live births, according to the latest demographic health survey at the time this study was conducted (18) ~~rates. The country's PMR stands at 33 per 1000 live births (22).,~~ This rate ~~which exceeds~~surpasses the regional average and falls significantly short of ~~reported for Southern Africa and remains well~~ short of the target set by the SDG and Zambia's Vision 2030 (19)~~(Republic of, 2006)(Zambia, 2006)(23)~~.

This study aimed to assess the predictors of perinatal death across~~in~~ all the major hospitals of Lusaka U~~u~~rban D~~d~~istrict – ~~, the most populous in~~ Zambia's most populous district and the one with the highest birth rate. In a~~A~~ddition~~ally~~, the research sought to generate~~provide~~ evidence ~~that~~to ~~could~~ inform targeted programmatic interventions and health systems strategies aimed at reducing the PMR withi~~i~~n this high-burden ~~contex~~setting.

Formatted: Indent: Left: 0", Right: 0.75", Space Before: 0 pt

General Objectives

Formatted: Font: Bold

Formatted: Indent: Left: 0"

The main objective of this study was to investigate the predictors of perinatal mortality at the seven main hospitals of Lusaka, Zambia

Specific Objectives

Formatted: Font: Bold

1. To determine the social and demographic predictors of perinatal mortality at the seven main hospitals of Lusaka, Zambia
2. To determine the maternal and foetal predictors of perinatal mortality at the seven main hospitals of Lusaka, Zambia

Formatted: Numbered + Level: 1 + Numbering Style: 1, 2, 3, … + Start at: 1 + Alignment: Left + Aligned at: 0.25" + Indent at: 0.5"

Materials and Methods

This was a multicentre study conducted at the seven major hospitals in Lusaka urban district, Zambia.

Data collection commenced on the 9th of September in 2023 and concluded on the 31st of January

Formatted: Indent: Left: 0"

Formatted: Superscript

~~31st in~~ 2024. Participants were prospectively recruited as cases occurred with controls. Eligible participants who were agreeable to participate after being provided with detailed information via a participant information sheet were then recruited after obtaining informed written consent. The participants included mothers who experienced~~had~~ stillbirths or early neonatal~~perinatal~~ deaths (cases) and those with live births (controls), all of whom were followed up before discharge. Data was collected using an interviewer-structured questionnaire. The hospitals involved all the major hospitals of Lusaka which include: two tertiary facilities - Women and Newborn and Levy Mwanawasa University Teaching Hospitals - and five first level hospitals: Chawama, Chilenje, Chipata, Kanyama, and Matero first level hospitals. According to the ~~2022~~ C~~c~~ensus R~~r~~eport, 2022~~,~~ the Lusaka is the most populous city in Zambia, with a female population of 1,590,922 (20)~~(24)~~.

Study design

This was an unmatched prospective case-control study with a 1:2 ratio, aimed at determining the predictors of perinatal mortality at the selected study site. Cases were defined as stillbirth after 24 weeks of gestation; ≥500g where gestational age was not clear; and early neonatal deaths within seven days of life from the study sites. For each case, two controls were selected through systematic random sampling, involving women who had live births immediately before or after occurrence of a case. Controls were recruited from the same facility where the cases occurred, and for early neonatal deaths, controls were mothers who delivered within 24hrs of case's birth.

A midwife from each labour and postnatal ward at each facility was trained and enrolled as data a collector. Data collectors provided participants with an information sheet and obtained informed consent. In rare cases where translation was necessary, data collectors translated the information into the appropriate language that participants understood and were ~~confortable~~more conversant~~comfortable~~ with. Data collection for postnatal mothers occurred whenever a case was

Commented [A3]: Dates format need to be consistent

identified (within the early neonatal

period) and controls were subsequently recruited. In rare cases, ~~especially~~particularly following an early neonatal death, controls were followed up before discharge or during clinic visits. These controls were mothers with live babies who delivered within 24 hours of a recruited case, ensuring their recruitment occurred around the same time as the case. ~~occurred.~~ Data were collected by reviewing files followed by interviewer administered collection of data using a standardized structured questionnaire on a google sheet that was managed by the Principal Investigator (PI). The PI monitored the Google data collection sheet ~~on a daily basis~~daily.

Study population

Study population comprised women aged 18 years and older who sought and received maternal and/or childbirth services at the study sites and met the inclusion criteria. Refer to the following: figure 1.0 flowchart illustrates the source of the sample, eligibility screening process and how the final sample was arrived at.

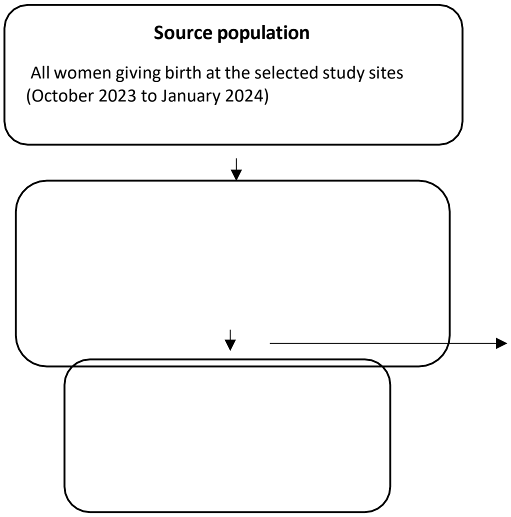

Formatted: Justified, Indent: Left: 0.25", Right: 0.75", Space Before: 15.85 pt, Line spacing: Double

Excluded

Five participants did not meet the inclusion criteria. (Withdrawn due to emotional distress)

Formatted: Left, Indent: Left: 0", First line: 0"

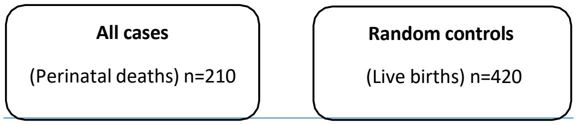

~~**Figure 1.0** *Selection of study participants' flow chart*~~

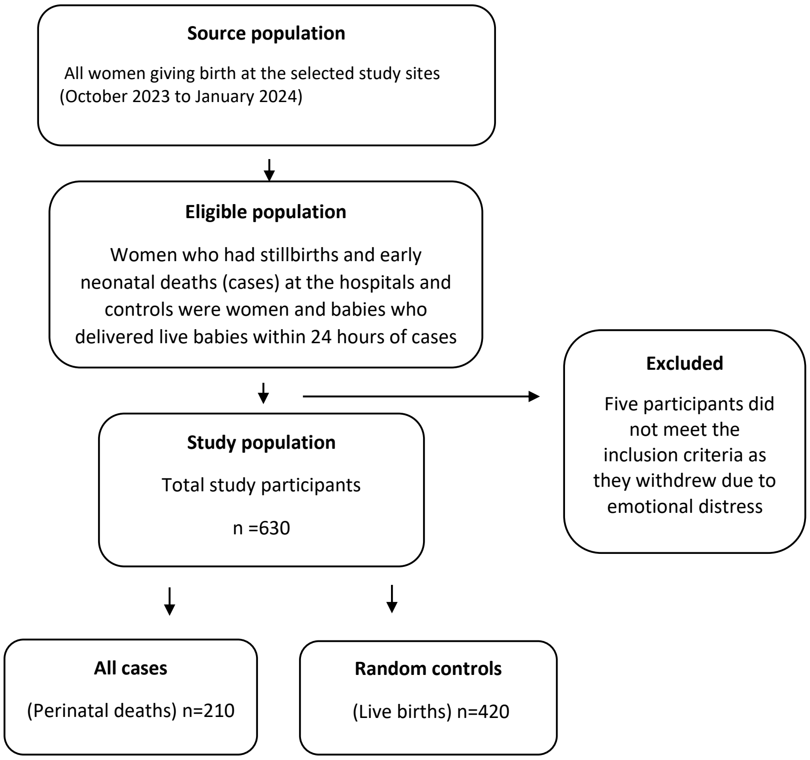

Figure 1.0 *Selection of study participants' flow chart*

Formatted: Normal, Indent: Left: 1.86", Space Before: 0 pt

Formatted: Font: Italic

Inclusion criteria

Cases included stillbirths from mothers aged above 18 years, with a gestational age above 24 weeks.. In the absence of gestational age a birthweight of ≥500g was considered eligible for this study. Early neonatal deaths were defined as deaths occurring within seven days of birth. Controls consisted of women above 18 years with live births, recruited within 24 hours of a corresponding case.

Commented [A4]: This definition did not fit here

Exclusion criteria

Women were excluded if they withdrew from the study, were under the age of 18, had a pregnancy of less than 24 weeks of gestation, or in the absence of gestational age, had a birthweight of less than 500g. WAdditionally, women were excluded if they were unavailable during the study period, were unable to participate due to ill health, or were too emotionally distressed to provide reliable information, even after reviewing the participant information sheet. Additionally, women who did not consent to participate were excluded.

Sample size calculation

The sample size for the study was calculated based on the assumptions that two controls per care ($r = 2$), with 80% power of the study (type II error 20%, i.e. $\beta = 0.2$), confidence interval of 95% with 5% type I error (i.e. $\alpha = 0.05$). With an assumed odds ratio of 2.5 for differences based on findings in previous similar studies. They reported for example associated potential risk factors included extremes of birthweight, post term delivery, infections, haemorrhage and previous history

or perinatal mortality (21, 22)~~(25, 26)~~. And using Zambia's PMR of 33 per 1000 live births (23)~~(Zambia Statistics Agency - ZSA et al., 2020)(22)~~, the following formula by Charan and Biswas (24)~~(27)~~ was used:

$$n = \frac{r+1}{r} \frac{p^{*}(1-p^{*})(Z_{\beta} + Z_{\alpha/2})^{2}}{(P_1 - P_2)^{2}}$$

Where n = is the sample size, p^{*}average proportion exposed which was given by the sum of proportion exposed cases (P_1) and proportion of control P_2 divided by 2 (i.e. = $\frac{P_1+P_2}{2}$), Z_{β} is the ~~proportion exposed cases (P_1) and proportion of control P_2 divided by 2 (i.e. = $\frac{P_1+P_2}{2}$), Z_{β} is the~~ standard normal value for power ~~(for~~(for power of 80%, Z_{β} = 0.84), $Z_{\alpha/2}$ is the standard normal value for the level of significance which is usually given as α=0.05 and ~~it's~~its value is 1.96, and r is the ratio of controls to cases, in our case the ratio is 2 to 1 which means our r = 2; To find P_2, we use the formula:

$P_2 = \frac{OR \times P_1}{P_1(OR-1)+1}$, where OR is the odds ratio and P_1 is the proportion with a specific risk factor in the control participants. Assuming OR = 3.2 based on pooled analysis with strong association of perinatal mortality due to lack of antenatal care (25)~~(13)~~: P_1=0.033 in the formula above we get the value of P_2 as 0.09845

$$P^{*} = \frac{0.033+0.09845}{2} = \frac{0.13145}{2} = 0.0657$$

Replacing the above information in the prescribed formula above the following was the targeted sample size:

$$n = \frac{2+1}{2}\left[\frac{0.0657(1-0.0657)(0.84+1.96)^2}{(0.09845-0.033)^2}\right]$$

$$n = 168.4874$$

$$n = 169$$

$$n = \frac{2+1}{2} \frac{0.0657(1-0.0657)(0.84+1.96)^2}{(}$$

$$= \frac{2+1}{2}\left[\frac{0.0657(1-0.0657)(0.84+1.96)^2}{(0.09845-0.033)^2}\right]$$

$0.09845 - 0.033)^2$

Formatted: Indent: Left: 0"

$$n = 168.4874$$

$$n = 169$$

Therefore, the calculated sample size was 169 cases and 338 using the 1:2 ratio, which gave 507. After factoring in 10% additional in case of participant fall out, the total sample size calculated was 558.

Formatted: Indent: Left: 0"

Sampling technique

To determine the sample size for each facility, using Probability Proportional to Size (PPS) sampling, we accounted for the delivery volume at each facility to ensure that larger facilities, which manage more deliveries, had a proportionally higher chance of selection. Hence, the following was applied in our study:

1. **To establishing facility delivery size**: We first reviewed delivery records from each facility covering January to December 2023 to estimate the delivery size at each one. This information served as the basis for determining the relative size of each facility.

2. **To ensure proportional allocation**: Facilities with higher delivery numbers were assigned a greater probability of selection compared to those with fewer deliveries. This allocation ensured that our sample would reflect the distribution of delivery cases across facilities, avoiding under-representation from larger facilities.

3. **To sample participants within facilities**: Once the facilities were selected with PPS, we then identified participants within each facility for inclusion in the study. This selection process within facilities allowed us to capture representative samples proportionate to the overall patient volume, aligning with the facility's estimated annual deliveries.

By using PPS sampling, we ensured a sample distribution that mirrors the scale of service utilization across facilities, thereby enhancing the representativeness and reliability of our findings.

A total of 635 participants were initially enrolled across all study sites. However, only 630 participants were included in the final analysis, as the remainder did not meet the inclusion criteria because they exceeded the early neonatal period. Data collection was through an interviewer administered questionnaire using a password controlled google sheet. Due to rarity of the outcome, purposeful sampling was used for the cases that met the inclusion criteria. For each case enrolled, two controls were randomly selected within the same 24-hour shift in which the case occurred.

Study variables

The outcome variables include macerated (a fresh stillbirth with features of maceration), fresh stillbirths (without features of maceration), and early neonatal death (occurred within 7 days of life). Distal level variables included age, marital status, parity, gravida, weight, BMI, employment status, educational level, distance of place of residence to health facility, and mode of transport. Intermediate level variables were antenatal booking data, number of antenatal contacts, medical history, history of pregnancy loss beyond viability, history of abortion, malaria during pregnancy, Human Immunodeficiency Virus (HIV) status, t~~T~~uberculosis during pregnancy, syphilis, alcohol intake during pregnancy, illicit drugs, smoking, folic and ferrous sulphate use during pregnancy, malaria treatment during pregnancy, and sickle cell anaemia. Rhesus factor, antepartum haemorrhage, preeclampsia, and eclampsia, preterm delivery, history of low birth weight, and inter-pregnancy interval were the rest. While proximal level variables included gestational age at the time of birth, outcome, Apgar score, time lag to Neonatal Intensive Care Unit (NICU), age of early neonatal at the time of demise, sex, gross congenital anomalies, congenital syphilis, prematurity, low birthweight, and cause of death.

Commented [A5]: I suggest you treat the sociodemographic factors such as age, gender, educational level etc, seperately

Commented [A6R5]: A previous reviewer had suggested to group the variables as distal, intermediate, and proximal

Data collection tool

A standard researcher administered questionnaire was set up using the google sheet platform with protected access to the selected data collectors only. The PI exclusively handled daily monitoring in real-time. After completion of data ~~collection~~collection, the data set was downloaded to an excel format for screening and cleaning and then coded to construct a do file for execution in Stata version 14.

Data analysis

Descriptive statistical analyses were conducted to summarize the proportions and frequencies of cases and controls. A bivariate analysis using Chi-square test was performed to identify the factors associated with perinatal mortality. All factors found to be associated with perinatal mortality in the bivariate analysis (*p*-value <0.05) at 95% confidence interval (95% CI) were then included in multivariable logistic regression model to adjust for potential confounders. Sequential elimination of predictors was employed to assess the strength and significance of the variables associated with perinatal deaths, followed by the discrimination and calibration. Continuous variables such as age, weight, parity, and gravidity were categorized based on clinical reasoning before being introduced into the model.

The ability of the model to correct risk was assessed using the Hosmer and Lemeshow goodness of fit test. A non-significant *p*-value (*p*>0.05) from this test indicated a good fit. Model stability was further evaluated by the measurement of the discrimination between participants with and without perinatal deaths using the area under the receiver operating characteristic (ROC) curve, with an acceptable values et at 0.7 (26)~~(28)~~. The study followed the Strengthening the Reporting of Observational Studies in Epidemiology (STROBE) guidelines (27)~~(29)~~.

Ethical considerations

Ethical approval for this study was obtained from the University of Zambia Biomedical Research Ethics Committee (UNZABREC), reference number 3712-2023. Prior to this, clearance was secured from the National Health Research Authority (NHRA), under reference number NHRA 000012/16/03/2023. All procedures adhered to the ethical guidelines for research involving human participants, including informed consent. Participants were thoroughly briefed about the study's purposed, procedures, potential risks, and benefits, and written informed consent obtained before enrolment.

Confidentiality and privacy were strictly maintained throughout the study. All personal identifying information was anonymized, and the data collected was stored on password-protected devices accessible only to the principal investigator (PI) and designated research team members. To ensure data security, encrypted backups were created, and physical access to data storage location was restricted. Participants were also informed of their right to withdraw from the study at any point without any consequences. The study adhered to national and international research ethics standards.

Results

Description of study participants

This study comprised a total of a total of 630 study respondents. The total perinatal deaths 210 (cases) and 420 with live births (controls) made the ratio of cases to controls 1:2. The following figure 2.0 hereafter shows the perinatal deaths distribution.

Commented [A7]: I suggest you start with respond rate of your study sybject

Formatted: Font: 12 pt

Formatted: Line spacing: Double

Formatted: Font: 12 pt, Not Bold

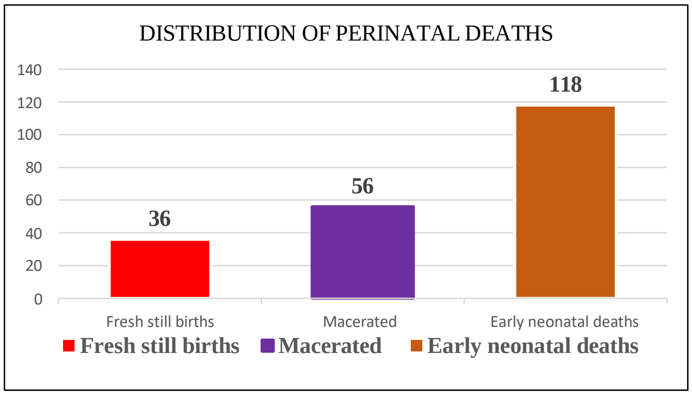

Figure 2.0 *distribution of perinatal mortality according to outcomes*

Figure 2.0 is a summary illustrating the frequency distribution of the Fresh Stillbirths (FSB), Macerated Stillbirth (MSB), and Early Neonatal Death (ENND) for this study. ~~The total perinatal deaths 210 (cases) and 420 with live births (controls) made the ratio of cases to controls 1:2.~~ The proportion of the perinatal outcomes was ~~ere Early Neonatal Death (ENND)~~ 118 (56%) for ENNDs, ~~Macerate Stillbirth (MSB)~~ 56 (27 %) MSBs, and 36 (17%), ~~Fresh Stillbirths (~~FSBs~~)~~ respectively.

Commented [A8]: I suggest you describe the figure 2.0 in the write up.
Eg. Figure 2.0 shows that….

Participants' socio-demographic characteristics

From the social-demographic attributes (distal level variables) of the respondents sampled majority of the respondents were between the age of 20-34 years 401 (63.7%). 19 years old and under were 90 (14.3%) and 97 (15.4%) were 35 years of age and above. Mean age for the study population was 27. Of the respondents 478 (75.9%) were Married and 143 (22.7%) were not married. A total of 487 (75.9%) had a parity between 1 to 4, while 81 (12.9%) were grandmultipara. Additionally, 445 (72.4%) were not employed and 170 (27.6%) employed. For education, the majority of the

respondents had only attained secondary level of education 331 (52.5%), primary 187 (29.7%), and 95 (15.1%) for tertiary. Most of the participants used public transport 415 (65.9%) while only 98 (15.6%) had personal transport and the others 106 (16.8%) on foot.

Formatted: Indent: Left: 0"

Results of predictors of perinatal mortality from bivariate analysis

Bivariate analysis using Chi-Square Test revealed several ~~variable~~variables associated with perinatal mortality. The outcomes outlined in the table below revealed that the level of education of the mother had a significant association with perinatal mortality (p<0.017). The form of transport used when going to the hospital (on foot) also had association with perinatal mortality (p<0.001). Antenatal Care (ANC) booking (first contact with healthcare provider for ANC) beyond 12 weeks of gestation was statistically significant with p<0.001. Women who had prior history of perinatal death also had a statistically significant p-value (p<0.001). Abortion and Anaemia during pregnancy were found with p <0.018 and p<0.003 respectively. The rest of the independent variables had no statistical significance.

Formatted: Indent: Left: 0"

Table 1.0 Summary of the maternal socio-demographic characteristics and pregnancy factors associated perinatal mortality

Predictors	Cases (n) 210 (%)	Controls (n) 420 (%)	Total (n) 630 (%)	χ^2 p-value
**Distal level variables**				
**Age**				
≤ 19	30 (14.3)	60 (14.3)	90 (14.3)	
20-34	138 (65.7)	263 (62.6)	401 (63.7)	0.466
> 35	27 (12.9)	70 (16.7)	97 (15.4)	
Missing	15 (7.1)	27 (6.4)	42 (6.7)	
**Marital status**				
Not married	50 (23.8)	93 (22.1)	143 (22.7)	0.741
Married	160 (76.2)	318 (75.7)	478 (75.9)	
Missing	0 (0.0)	9 (2.1)	9 (1.4)	
**Parity**				
1 – 4	170 (81.0)	309 (73.6)	487 (77.3)	0.702
> 5	36 (17.1)	62 (14.8)	81 (12.9)	
Missing	4 (1.9)	49 (11.7)	62 (9.8)	

**Employment Status**				
Not employment	151 (71.9)	294 (70.0)	445 (72.4)	0.610
Employed	54 (25.7)	116 (27.6)	170 (27.6)	
Missing	5 (2.4)	10 (2.4)	15 (2.4)	
**Educational level**				
Primary	65 (31.0)	122 (29.0)	187 (29.7)	
Secondary	121 (57.6)	210 (50.0)	331 (52.5)	**0.017***
Tertiary	20 (9.6)	75 (17.9)	95 (15.1)	
Missing	4 (1.9)	13 (3.1)	17 (2.7)	
**Intermediate level variables**				
**Mode of transport**				
Foot	50 (23.8)	56 (13.3)	106 (16.8)	**0.001***
Public transport	139 (66.2)	276 (65.7)	415 (65.9)	
Personal	20 (9.5)	78 (18.6)	98 (15.6)	
Missing	1 (0.5)	10 (2.4)	11 (1.7)	
**ANC Booking**				
< 12 weeks	100 (47.6)	83 (19.8)	183 (29.0)	
> 12 weeks	106 (50.5)	323 (76.9)	430 (68.3)	**0.001***
Missing	4 (1.9)	14 (3.3)	17 (2.7)	
**History of Stillbirth**				
Yes	64 (30.5)	32 (7.6)	96 (15.2)	**0.001***
No	143 (68.1)	377 (89.8)	520 (82.5)	
Missing	3 (1.4)	11 (2.6)	14 (2.2)	
**History of Abortion**				
Yes	10 (4.8)	7 (1.7)	17 (2.7)	**0.018***
No	181 (86.2)	393 (93.6)	574 (91.1)	
Missing	19 (9.0)	20 (4.8)	39 (6.2)	
**Proximal level variables**				
**Malaria during pregnancy**				
Yes	21 (10.0)	26 (6.2)	47 (7.5)	0.080
No	181 (86.2)	384 (91.4)	565 (89.7)	
Missing	8 (3.8)	10 (2.4)	18 (2.9)	
**HIV Status**				
Positive	30 (14.3)	59 (14.0)	89 (14.1)	0.898
Negative	173 (82.4)	351 (83.6)	524 (83.2)	
Missing	7 (3.3)	10 (2.4)	17 (2.7)	
**Tuberculosis during pregnancy**				
Yes	2 (1.0)	1 (0.2)	3 (0.5)	0.227
No	201 (95.7)	397 (94.5)	598 (94.9)	
Missing	7 (3.3)	22 (5.2)	29 (4.6)	
**Alcohol**				

Yes	14 (6.7)	42 (10.0)	56 (8.9)	0.155
No	193 (91.9)	368 (87.6)	561 (89.0)	
Missing	3 (1.4)	10 2.4)	13 (2.1)	
**Smoking**				
Yes	4 (1.9)	2 (0.5)	6 (1.0)	0.087
No	204 (97.1)	405 (96.4)	609 (96.7)	
Missing	2 (1.0)	13 (3.1)	15 (2.4)	
**Folate and Ferrous sulphate supplementation**				
Yes	197 (93.8)	396 (94.3)	593 (94.1)	0.307
No	10 (4.8)	13 (3.1)	23 (3.7)	
Missing	3 (1.4)	11 (2.6)	14 (2.2)	
**Anaemia in pregnancy**				
Yes	13 (6.2)	60 (14.3)	73 (11.6)	**0.003***
No	190 (90.5)	349 (83.1)	539 (85.6)	
Missing	7 (3.3)	11 (2.6)	18 (2.9)	
**Sickle cell anaemia**				
Yes	1 (0.5)	8 (1.9)	9 (1.0)	0.151
No	205 (97.6)	400 (95.2)	605 (96.0)	
Missing	4 (1.9)	12 (2.9)	19 (3.0)	
**Antepartum Haemorrhage**				
Yes	11 (5.2)	43 (10.2)	54 (8.6)	**0.058**
No	176 (83.8)	358 (85.2)	534 (84.8)	
Missing	23 (11.0)	19 (4.5)	42 (6.7)	
**Preeclampsia**				
Yes	20 (9.5)	47 (11.2)	67 (10.6)	0.863
No	160 (76.2)	358 (85.2)	518 (82.2)	
Missing	30 (14.3)	15 (3.6)	45 (7.1)	
**Hypertension**				
Yes	14 (6.7)	16 (3.8)	30 (4.8)	0.101
No	187 (89.0)	393 (93.6)	580 (92.1)	
Missing	9 (4.3)	11 (2.6)	20 (3.2)	
**Diabetes Mellitus**				
Yes	1 (0.5)	2 (0.5)	3 (0.5)	0.994
No	204 (97.1)	404 (96.2)	608 (96.5)	
Missing	5 (2.4)	14 (3.3)	19 (3.0)	

Unadjusted logistic regression of variables that are predictors of perinatal mortality

According to the bivariate logistic regression for predictors of perinatal death (table 2), respondents with a primary education level were almost two times likely to experience perinatal mortality compared to those the tertiary level group (COR=2.00, 95% CI: 1.12-3.56). The analysis also showed that the odds of experiencing perinatal death were over three times higher (COR=3.48, 95% CI: 1.87-6.49) for women who walked to access healthcare services and nearly two times as high (COR=1.96, 95% CI: 1.15-3.34) for those using public transport, compared to women who had personal transport.

Additionally, starting ANC after 12 weeks of gestation had over three and half odds (COR=3.68, 95% CI: 2.56-5.30) of perinatal mortality compared to those that started earlier. Women with previous history of stillbirth were almost five times more likely to experience perinatal mortality (COR=5.27, 95 CI: 3.31-8.402) compared to those without such a history. Women with history of abortion were also three times more likely (OR=3.10, 95% CI: 1.16-8.28) to experience perinatal mortality compared to those who had no history of abortion before. This study also found that women who experienced anaemia during pregnancy had more than two and half times higher odds of perinatal mortality (COR=2.51, 95 CI: 1.34-4.69) compared to those without anaemia.

~~In order to~~To understand the combined impact of predictors of perinatal mortality and to control for confounding, it required multivariate logistic regression analysis. Table 2.0 is the illustration of the final multivariate model that best describes predictors of perinatal mortality. Following a thorough analysis and correction for the impact of extraneous variables, four variables stood out as predictors of perinatal mortality.

While educational level after multivariate logistic regression analysis did not show significance as a predictor of perinatal mortality. Mode of transport to the healthcare facility, on the other hand, showed significance as a predictor of perinatal mortality. Women who walked to the hospital had three times the odds of perinatal mortality compared to those who used personal transport (AOR 3.48, 95% CI: 1.87–6.49, $p < 0.001$). Antenatal care (ANC) booking after 12 weeks of gestation was significantly associated with an increased risk of perinatal mortality. Women who booked ANC late had nearly three times higher odds of experiencing perinatal mortality compared to those who booked early (AOR 2.91, 95% CI: 1.97–4.29, $p < 0.001$). Maternal anemia during pregnancy was associated with a three-and-a-half-fold increased risk of perinatal mortality. Women with anemia had significantly higher odds of perinatal death compared to non-anemic women (AOR 3.58, 95% CI: 1.72–7.44, $p < 0.001$). A history of previous pregnancy loss was strongly associated with increased odds of perinatal mortality. Women with a history of stillbirth had five times the odds of perinatal death compared to those without such a history (AOR 5.05, 95% CI: 2.99–8.51, $p < 0.001$). Table 2.0 presents a summarized comparison of univariable and multivariable predictors following bivariable and then multivariable logistic regression analysis.

Table 2.0: Multivariable logistic regression analysis for factors associated with perinatal mortality in the selected study sites

**Univariable logistic regression**				**Multivariable logistic regression analysis**		
**Predictor variable**	**Crude Odds Ratio (COR)**	**95%CI**	**p-value**	**Adjusted Odds Ratio (AOR)**	**95% CI**	**P-value**
**Educational level**						
Tertiary	Ref					
Primary	2.00	(1.12-3.56)	0.019	1.66	(0.83-3.31)	0.152
Secondary	2.16	(1.26-3.71)	0.005	1.87	(0.98-3.57)	**0.058**
**Mode of Transport**						

Personal	Ref					
Foot	3.48	(1.87-6.49)	<0.001	2.75	(1.23-5.39)	**0.012***
Public	1.96	(1.15-3.34)	0.013	1.70	(0.92-3.16)	0.091
**ANC Booking**						
< 12 weeks	Ref			Ref		
> 12 weeks	3.68	(2.56-5.30)	<0.001*	2.91	(1.97-4.3)	**<0.001***
**History of Stillbirth**						
No	Ref			Ref		
Yes	5.27	(3.31-8.40)	<0.001*	5.05	(2.99-8.51)	**<0.001***
**History of abortion**						
No	Ref					
Yes	3.10	(1.16-8.28)	0.024	0.86	(0.24-3.06)	0.814
**Anaemia during pregnancy**						
No	Ref			Ref		
Yes	2.51	(1.34-4.69)	0.004	3.58	(1.72-7.44)	**0.001***

Discussion

The objective of this study was to identify predictors of perinatal mortality in Lusaka, Zambia. The selected sites are the major hospitals of Lusaka and referral hospitals that anchor all maternal and perinatal health services. Among the socio-demographic variables, educational level and mode of transport were the only variables strongly associated with perinatal mortality. The statistically significant predictors of perinatal mortality identified in this study were mode of transport used by women when accessing healthcare, timing of antenatal care (ANC) bookings, history of abortion, stillbirth, and anaemia during pregnancy.

In clinical settings, pregnancy in women of advanced maternal age (AMA) (i.e. maternal age >35) is frequently labelled as “high-risk even in the absence of obvious risk factors. This classification has been validated by many authors from a meta-analysis on “advanced maternal age: adverse outcomes of pregnancy”, where it was reported that AMA women had higher perinatal mortality and stillbirths compared to women of 20 – 34 maternal age group (28)(30). On the contrary, in

this

study maternal age was not found to have significant association with perinatal mortality. Educational level and mode of transport are the other socio-demographic factors that demonstrated association with our outcome of interest. However, during univariable and multivariable logistic regression analysis only the mode of transport remained statistically significant as a predictor of perinatal mortality. While education level did not show strong association with perinatal mortality in this investigation. A study on effect of maternal education and perinatal outcome done in Punjab, India reported that lower educational status was a significantly important predictor of adverse perinatal outcomes including perinatal mortality (29)~~(31)~~.

Formatted: Indent: Left: 0"

This study revealed that macerated stillbirths and early neonatal deaths (ENND) had a higher occurrence among the perinatal mortality cases, consistent with findings reported in other studies~~ (32, 33) ~~(30, 31). Another study by Shelke et.al (32)~~ (34)~~ similarly reported a higher proportion of macerated stillbirths compared to fresh stillbirths, which may reflect the quality of prenatal and obstetric care, while macerated stillbirths are typically the results of intrauterine foetal death occurring more than 6 to 12~~8~~ hours prior to delivery~~labour~~ (2, 5, 33)~~(35)~~. Contributing factors to macerated stillbirths include intrauterine growth restriction, placental lesions, and infections, though a large proportion of these deaths remain unexplained ~~(36)~~(34). In this study, these cases also remained unexplained, underscoring the need for further investigation into the underlying causes and improved data collection practices. Early neonatal deaths are often caused by complications such as low birth weight, birth asphyxia, and sepsis, which require neonatal intensive care (37,~~-~~39) (35, 36). However, in this study, the causes of early neonatal mortality could not be determined because of the nature of the study design that could measure association.

Among the distal level variables none of them had statistical significance as a ~~predictors of perinatal mortality~~predictor of perinatal mortality. On the other ~~hand~~hand, it was mainly the intermediate

level variables that had

significant association with perinatal mortality: mode of transport, ANC booking, and history of stillbirth. Whereas only anaemia during pregnancy was found to be a significant predictor of perinatal mortality among the proximal level variables. The mode of transport showed statistical significance as a predictor of perinatal mortality. This was because women who walked to the healthcare facility to seek medical care during pregnancy were found to be three times more likely to experience perinatal death compared to those with personal transportation. Those using public transport also had two times higher risk of perinatal mortality experience. This finding aligns with a previous study done in Zambia and Uganda, which identified a significant difference in perinatal outcomes based on motorized vs non-motorized means of transport (37)(40). Factors contributing to this difference may include poor physical access to healthcare, lack of empowerment for decision- making, and limited health education (38)(41). Furthermore, women who relied on non-motorized transport, such as walking to seek medical care - as was observed in this study - were more likely to access Basic Emergency Obstetric and Neonatal Care (BEmONC) facilities. In contrast, those using motorized transport who had access to Comprehensive Emergency Obstetrics and Neonatal Care (CEmONC) facilities, which provide superior care and improved maternal and perinatal outcomes, particularly when staffed with trained personnel and equipped with essential resources (42, 43) (39, 40).

Formatted: Indent: Left: 0"

The timing of ANC bookings also played a significant role in perinatal outcomes. The WHO recommends that the first ANC visit should occur within the first trimester, up to 12 weeks of gestation (41)(44). In this study, over 70% of the women booked late for ANC (> 12 weeks), and late booking was associated with a threefold increased risk of perinatal mortality. This finding corroborated other studies, which reported that delayed ANC increases the risk of complications during pregnancy and delivery, contributing to perinatal mortality (45, 46) (42, 43).

A previous history of abortion did not emerge as a significant predictor of perinatal mortality in this study. However, a history of abortion can increase the risk of perinatal mortality compared to those without such a history. This may be attributed to a higher susceptibility to infections during subsequent pregnancies among women with ~~a~~ history of abortion (44)~~(47)~~, which raises the likelihood of perinatal mortality (45)~~(48)~~. Conversely, ~~a~~ previous history of stillbirth, in this study, was significantly associated with a fivefold increase in the risk of perinatal mortality. This finding aligns with results from other studies in Zambia (46)~~(49)~~ and Zimbabwe (47)~~(50)~~, which similarly reported that women with a history of stillbirth faced ~~a~~ higher risk of perinatal mortality. This also aligns with findings from a study conducted in Ethiopia (44)~~(47)~~, which demonstrated that women with a history of stillbirth were at a higher risk of infections during subsequent pregnancies. These infections can compromise foetal health, leading to complications that significantly elevate the likelihood of perinatal mortality (45)~~(48)~~. Despite such findings from other studies linking infections to perinatal mortality, infections were not significantly associated with perinatal mortality in this study. Additionally, many stillbirths remain unexplained, largely due to the low availability of post-mortem investigations (48)~~(51)~~. This lack of clear causality makes discussing stillbirths challenging, not only for the parents but also for healthcare professionals, who often face difficulties in obtaining consent for post-mortem investigation aimed at determining the cause of death (49)~~(52)~~.

Among the medical complications, anaemia during pregnancy was the strongest predictor of perinatal mortality, with women suffering from anaemia showing a two-and-a-half-fold increased risk, compared to those without anaemia. Anaemia is closely linked to small-for-gestational-age infants and preterm birth, both of which are significant risk factors for perinatal mortality

(46)~~(49)~~.

While previous studies in Zambia ~~(25, 53)~~ (21, 50) identified small-for-gestational-age infants, preterm birth, and low birth weight as high-risk factors for perinatal mortality, this study did not find these findings to have significant association with perinatal mortality.

Study Implications

Based on our study findings, socio-demographic factors like lower education level (primary and secondary) and mode of transport showed association with higher likelihood of perinatal mortality, although the latter was not statistically significant in the adjusted model. Participants with personal transport were significantly associated with lower odds of perinatal mortality even in the adjusted multivariable logistic regression model compared to those using public or walked to seek healthcare service. This may be suggestive of other factors like lower socioeconomic status and transport accessibility play a significant role in influencing pregnancy outcomes. Further interventions should focus on tackling such socio-demographic issues like improving transport accessibility and education.

The study also highlighted several other factors, such as late ANC booking, a history of stillbirth, and anaemia during pregnancy, which remained significantly associated with higher odds of perinatal mortality even after multivariable regression analysis. These findings imply that health policies should prioritize improving early ANC attendance and targeting high-risk pregnancies, particularly such as anaemia in pregnancy and history of stillbirth should be the centre theme for developing interventions for positive pregnancy outcomes. In this vein, future research should explore potential interventions to reduce the effects of these predictors and improve perinatal outcomes.

Strengths and Limitations

This study provides a comprehensive assessment of multiple predictors of perinatal mortality simultaneously, offering a nuanced understanding of the factors influencing perinatal outcomes in Lusaka district. The sample is representative of the broader population, as data were collected from all major hospitals in the city, ensuring a diverse range of cases that enhances the generalizability of the findings. This broad representation strengthens the relevance of the study to policymakers and healthcare practitioners, as the insights derived can inform targeted interventions and resource allocation in public health strategies. Furthermore, identifying significant predictors not only contributes to the existing body of literature but also equips clinicians with evidence-based knowledge to improve maternal and neonatal care practices.

One notable limitation is the incompleteness of information during data collection, primarily due to inconsistencies in medical record-keeping, and data entry errors which resulted in gaps in some variables. Therefore, some of the variables such as congenital syphilis and congenital anomalies resulted in not being included in the analysis. Additionally, accurately stating or recording the cause of death in stillbirth cases remains challenging, as investigations are not routinely conducted to determine the cause of death. These limitations highlight the need for improved data collection practices and post-mortem investigations to enhance the accuracy and reliability of future research in this area.

Conclusion

In conclusion, this study highlights several critical predictors of perinatal mortality in Lusaka, Zambia, including mode of transport, timing of antenatal care booking, previous stillbirths, and maternal anaemia. Women who walked or used public transport to access healthcare faced higher risks of perinatal mortality, underscoring the socioeconomic disparities affecting access to quality

obstetric care. The late initiation of antenatal care compromised early detection and management

of complications, emphasizing the need for public health initiatives to promote timely antenatal care. Additionally, history of stillbirth significantly increased the risk of subsequent perinatal mortality, highlighting the necessity for psychological and medical support for women with previous adverse pregnancy outcomes. Maternal anaemia was also a substantial risk factor, correlating with adverse birth outcomes such as small for gestational age and preterm births. While the study did not find small for gestational age or preterm birth to be direct predictors, these findings align with existing literature. Overall, targeted interventions addressing these predictors—such as improving transportation options, enhancing antenatal care accessibility, and providing support for women with prior adverse pregnancy experiences—could significantly reduce perinatal mortality rates and improve maternal and neonatal health outcomes in Lusaka. The aim of this study was to identify the predictors of perinatal mortality in Lusaka, Zambia.

Recommendations

According to this study findings, ~~in order to~~to help to reduce the perinatal mortality in Lusaka, Zambia: there is need to improve transportation to enhance options for pregnant women especially in the underserved areas; need to ensure timely access to Comprehensive Emergency Obstetrics and Neonatal Care (CEmONC) facilities; Early ANC booking needs to be promoted through strengthening education and outreach programs to encourage women to book for ANC within the first trimester of pregnancy. In addition, to address anaemia during pregnancy routine screening and treatment need to be strengthened to reduce the associated risks to perinatal mortality. Women with previous experience of perinatal mortality need targeted support and monitoring to minimise risks in subsequent pregnancies. This support includes but not limited to enhanced quality prenatal and obstetrics care investment focusing on early detection and management of pregnancy complications. Finally, community-based health education and

empowerment programs to equip women with knowledge and resources for better pregnancy outcomes and self-autonomy in decision-making.

Acknowledgements

I would like to thank all my supervisors for the guidance provided to complete this study. I also would like to acknowledge the immense contribution the study participants for accepting to be part of the project.

Conflict of interest

We declare no conflict of interest.

Authors' contributions

MM: conceptualization of the study, study design, data collection, data cleaning, data processing and analysis, and manuscript writing. PK: supervision and methodology. CJ: supervision and contextualization of the project. MM: conceptualization and contextualization of the study. BV: overall content supervision, contextualization of the study, and methodology. All authors have read and approved the final version of the manuscript.

References

1. Organization WH. Neonatal and perinatal mortality: country, regional and global estimates: World Health Organization; 2006.
2. Gold KJ, Abdul-Mumin AR, Boggs ME, Opare-Addo HS, Lieberman RW. Assessment of "fresh" versus "macerated" as accurate markers of time since intrauterine fetal demise in low-income countries. Int J Gynaecol Obstet. 2014;125(3):223-7.
3. Lawn JE, Gravett MG, Nunes TM, Rubens CE, Stanton C, Group GR. Global report on preterm birth and stillbirth (1 of 7): definitions, description of the burden and opportunities to improve data. BMC pregnancy and childbirth. 2010;10:1-22.
4. Shanklin DR. FETAL MACERATION. 2. AN ANALYSIS OF 53 HUMAN STILLBORN INFANTS. Am J Obstet Gynecol. 1964;88:224-9.
5. Organization WH. Health in 2015 from MDGs, Millenium Development Goals to SDGs, Sustaibale Development Goals. Health from MDGs to SDGs. 2015;1(2015):216-.
6. De Bernis L, Kinney MV, Stones W, ten Hoope-Bender P, Vivio D, Leisher SH, et al. Stillbirths: ending preventable deaths by 2030. The lancet. 2016;387(10019):703-16.
7. Organization WH. Trends in maternal mortality 2000 to 2017: estimates by WHO, UNICEF, UNFPA, World Bank Group and the United Nations Population Division. 2019.
8. Say L, Chou D, Gemmill A, Tunçalp Ö, Moller AB, Daniels J, et al. Global causes of maternal death: A WHO systematic analysis. The Lancet Global Health. 2014;2(6):323-33.
9. Lawn JE, Blencowe H, Waiswa P, Amouzou A, Mathers C, Hogan D, et al. Stillbirths: rates, risk factors and potential for progress towards 2030. Lancet. 2016;387(10018):587-603.
10. Gaym A. Perinatal mortality audit at Jimma hospital, South-Western Ethiopia, 1990-1999. The Ethiopian Journal of Health Development. 2000;14(3).
11. Briand V, Dumont A, Abrahamowicz M, Sow A, Traore M, Rozenberg P, et al. Maternal and perinatal outcomes by mode of delivery in Senegal and Mali: a cross-sectional epidemiological survey. 2012.
12. Aragaw Y. Perinatal mortality and associated factor in Jimma university specialized hospital, South West Ethiopia. Gynecol Obstet (Sunnyvale). 2016;6(409):2161-0932.
13. Berhan Y, Berhan A. Meta-analysis of selected maternal and fetal factors for perinatal mortality. Ethiopian journal of health sciences. 2014;24:55-68.
14. Wang H, Bhutta ZA, Coates MM, Coggeshall M, Dandona L, Diallo K, et al. Global, regional, national, and selected subnational levels of stillbirths, neonatal, infant, and under-5 mortality, 1980–2015: a systematic analysis for the Global Burden of Disease Study 2015. The Lancet. 2016;388(10053):1725-74.
15. De Bernis L, Kinney MV, Stones W, Ten Hoope-Bender P, Vivio D, Leisher SH, et al. Stillbirths: Ending preventable deaths by 2030. The Lancet: Lancet Publishing Group; 2016. p. 703-16.
16. Blencowe H, Cousens S, Jassir FB, Say L, Chou D, Mathers C, et al. National, regional, and worldwide estimates of stillbirth rates in 2015, with trends from 2000: a systematic analysis. The Lancet Global Health. 2016;4(2):e98-e108.
17. Kumsa H, Mislu E, Yimer NB. A systematic review and meta-analysis of the globally reported International Classification of Diseases to Perinatal Mortality (ICD-PM). Frontiers in medicine. 2024;11:1434380.
18. Valenzuela CP, Gregory EC, Martin JA. Perinatal Mortality in the United States, 2020–2021: US Department of Health and Human Services, Centers for Disease Control and ...; 2023.
19. Bezie MM, Asebe HA, Asnake AA, Fente BM, Negussie YM, Asmare ZA, et al. Factors associated with perinatal mortality in sub-Saharan Africa: A multilevel analysis. PloS one. 2024;19(11):e0314096.

20. Akombi BJ, Renzaho AM. Perinatal mortality in sub-saharan africa: A meta-analysis of demographic and health surveys. Annals of Global Health. 2019;85(1):1-8.
21. Tiruneh D, Assefa N, Mengiste B. Perinatal mortality and its determinants in Sub Saharan African countries: systematic review and meta-analysis. Maternal Health, Neonatology and Perinatology. 2021;7(1):1-17.
22. Central Statistics Office MoH. Zambia Demographic and Health Survey. 2018. Report No.: 9788578110796.
23. MoF GRZ. Vision 2030. Lusaka: Republic of Zambia; 2006.
24. Worldbank. Fertility rate, total (births per woman) - Zambia. THE World Bank - IBRD - IDA2020.
25. Stringer EM, Vwalika B, Killam WP, Giganti MJ, Mbewe R, Benjamin BH, et al. Determinants of stillbirth in Zambia. Obstetrics and Gynecology. 2011;117(5):1151-9.
26. Chuwa FS, Mwanamsangu AH, Brown BG, Msuya SE, Senkoro EE, Mnali OP, et al. Maternal and fetal risk factors for stillbirth in Northern Tanzania: A registry-based retrospective cohort study. PloS one. 2017;12(8):e0182250-e.
27. Charan J, Biswas T. How to calculate sample size for different study designs in medical research? Indian journal of psychological medicine. 2013;35(2):121-6.
28. Kleinbaum DG, Klein M. Survival analysis: a self-learning text: Springer; 2012.
29. Von Elm E, Altman DG, Egger M, Pocock SJ, Gøtzsche PC, Vandenbroucke JP, et al. The Strengthening the Reporting of Observational Studies in Epidemiology (STROBE) statement: guidelines for reporting observational studies. Annals of internal medicine. 2007;147(8):573-7.
30. Pinheiro RL, Areia AL, Mota Pinto A, Donato H. Advanced maternal age: adverse outcomes of pregnancy, a meta-analysis. Acta medica portuguesa. 2019;32(3):219-26.
31. Chopra I, Juneja SK, Sharma S. Effect of maternal education on antenatal care utilization, maternal and perinatal outcome in a tertiary care hospital. Int J Reprod Contraception, Obstet Gynecol. 2018;8(1):247.
32. Abdulhameed AL, Aljammas EK, Ramzi A. Perinatal mortality types. Sulaymania, Iraq: Department of Family and Community Medicine, College of Medicine. 2016:366-72.
33. Mustufa MA, Kulsoom S, Sameen I, Moorani KN, Memon AA, Korejo R. Frequency of Stillbirths in a Tertiary Care Hospital, Karachi. Pak J Med Sci. 2016;32(1):91-4.
34. Shelke SP, Shivkumar PV, Chaurasia AK. Incidence of stillbirth in relation to period of delivery, socioeconomic status and period of gestation in a rural tertiary care hospital-mgims, sewagram within study period of three years (2013–2016). The Journal of Obstetrics and Gynecology of India. 2021:1-5.
35. Langley F. The perinatal postmortem examination. Journal of clinical pathology. 1971;24(2):159-69.
36. Man J, Hutchinson JC, Heazell AE, Ashworth M, Levine S, Sebire NJ. Stillbirth and intrauterine fetal death: factors affecting determination of cause of death at autopsy. Ultrasound in Obstetrics & Gynecology. 2016;48(5):566-73.
37. Gaíva MAM, Bittencourt RM, Fujimori E. Early and late neonatal death: characteristics of mothers and newborn. Revista Gaúcha de Enfermagem. 2013;34:91-7.
38. Ahmed Tahir Ahmed Abdifatah Elmi F. Determinants of early neonatal mortality (hospital based retrospective cohort study in Somali region of Ethiopia). Determinants of early neonatal mortality (hospital based retrospective cohort study in Somali region of Ethiopia). 2023;13(1114):8.
39. Bogale TN, Worku AG, Bikis GA, Kebede ZT. Why gone too soon? Examining social determinants of neonatal deaths in northwest Ethiopia using the three delay model approach. BMC Pediatrics. 2017;17(1):216.
40. Sacks E, Vail D, Austin-Evelyn K, Greeson D, Atuyambe LM, Macwan'gi M, et al. Factors influencing modes of transport and travel time for obstetric care: a mixed methods study in Zambia and Uganda. Health Policy and Planning. 2015;31(3):293-301.

~~41. Ayalew Tiruneh G, Melkamu Asaye M, Solomon AA, Tiruneh Arega D. Delays during emergency obstetric care and their determinants among mothers who gave birth in South Gondar zone hospitals, Ethiopia. A cross-sectional study design. Global Health Action. 2021;14(1):1953242.~~
~~42. Widyantari KY, Sulistyaningsih I, editors. The Effectiveness of Cemonc on Maternal Mortality: Systematic Literature Review. Proceeding International Conference; 2019.~~
~~43. Yusrawati Y, Respati T, Sekarwana N. Factors to improve the quality of comprehensive emergency obstetric and newborn care. International Journal of Public Health. 2023;12(3):1030-9.~~
~~44. Organization WH. WHO antenatal care recommendations for a positive pregnancy experience: nutritional interventions update: multiple micronutrient supplements during pregnancy. 2020.~~
~~45. Girum T. Alternative & Integrative Medicine. 2016.~~
~~46. Carroli G, Rooney C, Villar J. How effective is antenatal care in preventing maternal mortality and serious morbidity? An overview of the evidence. Paediatric and perinatal Epidemiology. 2001;15:1-42.~~
~~47. Feleke BE, Feleke TE. A longitudinal study on the effects of previous stillbirth or abortion on subsequent pregnancies and infants. European journal of public health. 2021;31(6):1237-41.~~
~~48. Suleiman MB, Mokuolu OA. Perinatal mortality in a Northwestern Nigerian city: a wake up call. Frontiers in Pediatrics. 2014;2:105.~~
~~49. Makasa M, Mutale W, Lubeya MK, Mpetemoya T, Chinayi M, Mangala B, et al. Determinants of stillbirth in the Five General Hospitals of Lusaka, Zambia: A Case-Control study. Medical Journal of Zambia. 2023;50(1):35-46.~~
~~50. Dube K, Lavender T, Blaikie K, Sutton CJ, Heazell AE, Smyth RM. Identification of factors associated with stillbirth in Zimbabwe–a cross sectional study. BMC pregnancy and childbirth. 2021;21:1-14.~~
~~51. Gardosi J, Kady SM, McGeown P, Francis A, Tonks A. Classification of stillbirth by relevant condition at death (ReCoDe): population based cohort study. Bmj. 2005;331(7525):1113-7.~~
~~52. Heazell A, McLaughlin MJ, Schmidt E, Cox P, Flenady V, Khong T, et al. A difficult conversation? The views and experiences of parents and professionals on the consent process for perinatal postmortem after stillbirth. BJOG: An international journal of obstetrics & Gynaecology. 2012;119(8):987-97.~~
~~53. Chibwesha CJ, Zanolini A, Smid M, Vwalika B, Kasaro MP, Mwanahamuntu M, et al. Predictors and outcomes of low birth weight in Lusaka, Zambia. International Journal of Gynecology & Obstetrics. 2016;134(3):309-14.~~

~~54.~~
1. Organization WH. Health in 2015: from MDGs, millennium development goals to SDGs, sustainable development goals. Health in 2015: from MDGs, Millennium Development Goals to SDGs, Sustainable Development Goals2015.
2. Lawn JE, Blencowe H, Waiswa P, Amouzou A, Mathers C, Hogan D, et al. Stillbirths: rates, risk factors, and acceleration towards 2030. The Lancet. 2016;387(10018):587-603.
3. De Bernis L, Kinney MV, Stones W, ten Hoope-Bender P, Vivio D, Leisher SH, et al. Stillbirths: ending preventable deaths by 2030. The lancet. 2016;387(10019):703-16.
4. Organization WH. Neonatal and perinatal mortality: country, regional and global estimates: World Health Organization; 2006.
5. Gold KJ, Abdul-Mumin AR, Boggs ME, Opare-Addo HS, Lieberman RW. Assessment of "fresh" versus "macerated" as accurate markers of time since intrauterine fetal demise in low-income countries. Int J Gynaecol Obstet. 2014;125(3):223-7.
6. Shanklin DR. FETAL MACERATION. 2. AN ANALYSIS OF 53 HUMAN STILLBORN INFANTS. Am J Obstet Gynecol. 1964;88:224-9.

Formatted: Heading 1, Right: 0", Tab stops: Not at 0.75"

Formatted: Condensed by 0.1 pt

Formatted: Normal, No bullets or numbering

7. Wang H, Bhutta ZA, Coates MM, Coggeshall M, Dandona L, Diallo K, et al. Global, regional, national, and selected subnational levels of stillbirths, neonatal, infant, and under-5 mortality, 1980–2015: a systematic analysis for the Global Burden of Disease Study 2015. The Lancet. 2016;388(10053):1725-74.
8. Lawn JE, Blencowe H, Pattinson R, Cousens S, Kumar R, Ibiebele I, et al. Stillbirths: Where? When? Why? How to make the data count? The Lancet. 2011;377(9775):1448-63.
9. Organization WH. Trends in maternal mortality 2000 to 2017: estimates by WHO, UNICEF, UNFPA, World Bank Group and the United Nations Population Division: executive summary. 2019.
10. Say L, Chou D, Gemmill A, Tunçalp Ö, Moller A-B, Daniels J, et al. Global causes of maternal death: a WHO systematic analysis. The Lancet global health. 2014;2(6):e323-e33.
11. Berhan Y, Berhan A. Meta-analysis of selected maternal and fetal factors for perinatal mortality. Ethiopian journal of health sciences. 2014;24:55-68.
12. Blencowe H, Cousens S, Jassir FB, Say L, Chou D, Mathers C, et al. National, regional, and worldwide estimates of stillbirth rates in 2015, with trends from 2000: a systematic analysis. The Lancet Global Health. 2016;4(2):e98-e108.
13. Kumsa H, Mislu E, Yimer NB. A systematic review and meta-analysis of the globally reported International Classification of Diseases to Perinatal Mortality (ICD-PM). Frontiers in medicine. 2024;11:1434380.
14. Valenzuela CP, Gregory EC, Martin JA. Perinatal Mortality in the United States, 2020–2021: US Department of Health and Human Services, Centers for Disease Control and …; 2023.
15. Bezie MM, Asebe HA, Asnake AA, Fente BM, Negussie YM, Asmare ZA, et al. Factors associated with perinatal mortality in sub-Saharan Africa: A multilevel analysis. PloS one. 2024;19(11):e0314096.
16. Akombi BJ, Renzaho AM. Perinatal mortality in sub-Saharan Africa: a meta-analysis of demographic and health surveys. Annals of global health. 2019;85(1):106.
17. Tiruneh D, Assefa N, Mengiste B. Perinatal mortality and its determinants in Sub Saharan African countries: systematic review and meta-analysis. Maternal health, neonatology and perinatology. 2021;7:1-17.
18. Zambia Statistics Agency - ZSA, Ministry of Health - MOH, University Teaching Hospital Virology Laboratory - UTH-VL, ICF. Zambia Demographic and Health Survey 2018. Lusaka, Zambia: ZSA, MOH, UTH-VL and ICF; 2020.
19. Republic of Z. Vision 2030: A prosperous middle-income nation by 2030. Ministry of Finance and National Planning Lusaka; 2006.
20. Zambia Statistics Agency - ZSA. 2022 CENSUS OF POPULATION AND HOUSING. 2022.
21. Stringer EM, Vwalika B, Killam WP, Giganti MJ, Mbewe R, Chi BH, et al. Determinants of stillbirth in Zambia. Obstet Gynecol. 2011;117(5):1151-9.
22. Chuwa FS, Mwanamsangu AH, Brown BG, Msuya SE, Senkoro EE, Mnali OP, et al. Maternal and fetal risk factors for stillbirth in Northern Tanzania: A registry-based retrospective cohort study. PloS one. 2017;12(8):e0182250.
23. Zambia Statistics Agency ZSA, Ministry of Health MOH, University Teaching Hospital Virology Laboratory U-V, Icf. Zambia Demographic and Health Survey 2018. Lusaka, Zambia: ZSA, MOH, UTH-VL and ICF; 2020.
24. Charan J, Biswas T. How to calculate sample size for different study designs in medical research? Indian journal of psychological medicine. 2013;35(2):121-6.
25. Aragaw Y. Perinatal mortality and associated factor in Jimma university specialized hospital, South West Ethiopia. Gynecol Obstet (Sunnyvale). 2016;6(409):2161-0932.
26. Kleinbaum DG, Klein M. Survival analysis a self-learning text: Springer; 1996.
27. Von Elm E, Altman DG, Egger M, Pocock SJ, Gøtzsche PC, Vandenbroucke JP. The Strengthening the Reporting of Observational Studies in Epidemiology (STROBE) statement: guidelines for reporting observational studies. The lancet. 2007;370(9596):1453-7.
28. Pinheiro RL, Areia AL, Mota Pinto A, Donato H. Advanced maternal age: adverse outcomes of pregnancy, a meta-analysis. Acta medica portuguesa. 2019;32(3):219-26.
29. Chopra I, Juneja SK, Sharma S. Effect of maternal education on antenatal care utilization, maternal and perinatal outcome in a tertiary care hospital. Int J Reprod Contraception, Obstet Gynecol. 2018;8(1):247.
30. Abdulhameed AL, Aljammas EK, Ramzi A. Perinatal mortality types. Sulaymania, Iraq: Department of Family and Community Medicine, College of Medicine. 2016:366-72.

31. Mustufa MA, Kulsoom S, Sameen I, Moorani KN, Memon AA, Korejo R. Frequency of Stillbirths in a Tertiary Care Hospital, Karachi. Pak J Med Sci. 2016;32(1):91-4.
32. Shelke SP, Shivkumar PV, Chaurasia AK. Incidence of Stillbirth in Relation to Period of Delivery, Socioeconomic Status and Period of Gestation in a Rural Tertiary Care Hospital-MGIMS, Sewagram Within Study Period of Three Years (2013-2016). J Obstet Gynaecol India. 2022;72(Suppl 1):1-5.
33. Langley F. The perinatal postmortem examination. Journal of clinical pathology. 1971;24(2):159-69.
34. Man J, Hutchinson JC, Heazell AE, Ashworth M, Levine S, Sebire NJ. Stillbirth and intrauterine fetal death: factors affecting determination of cause of death at autopsy. Ultrasound in Obstetrics & Gynecology. 2016;48(5):566-73.
35. Gaíva MAM, Bittencourt RM, Fujimori E. Early and late neonatal death: characteristics of mothers and newborn. Revista Gaúcha de Enfermagem. 2013;34:91-7.
36. Bogale TN, Worku AG, Bikis GA, Kebede ZT. Why gone too soon? Examining social determinants of neonatal deaths in northwest Ethiopia using the three delay model approach. BMC Pediatrics. 2017;17(1):216.
37. Sacks E, Vail D, Austin-Evelyn K, Greeson D, Atuyambe LM, Macwan'gi M, et al. Factors influencing modes of transport and travel time for obstetric care: a mixed methods study in Zambia and Uganda. Health Policy and Planning. 2015;31(3):293-301.
38. Ayalew Tiruneh G, Melkamu Asaye M, Solomon AA, Tiruneh Arega D. Delays during emergency obstetric care and their determinants among mothers who gave birth in South Gondar zone hospitals, Ethiopia. A cross-sectional study design. Global Health Action. 2021;14(1):1953242.
39. Yusrawati Y, Respati T, Sekarwana N. Factors to improve the quality of comprehensive emergency obstetric and newborn care. International Journal of Public Health. 2023;12(3):1030-9.
40. Widyantari KY, Sulistyaningsih I, editors. The Effectiveness of Cemonc on Maternal Mortality: Systematic Literature Review. Proceeding International Conference; 2019.
41. Organization WH. WHO antenatal care recommendations for a positive pregnancy experience: nutritional interventions update: multiple micronutrient supplements during pregnancy. 2020.
42. Girum T. Alternative & Integrative Medicine. 2016.
43. Carroli G, Rooney C, Villar J. How effective is antenatal care in preventing maternal mortality and serious morbidity? An overview of the evidence. Paediatric and perinatal Epidemiology. 2001;15:1-42.
44. Feleke BE, Feleke TE. A longitudinal study on the effects of previous stillbirth or abortion on subsequent pregnancies and infants. European journal of public health. 2021;31(6):1237-41.
45. Suleiman MB, Mokuolu OA. Perinatal mortality in a Northwestern Nigerian city: a wake up call. Frontiers in Pediatrics. 2014;2:105.
46. Makasa M, Mutale W, Lubeya MK, Mpetemoya T, Chinayi M, Mangala B, et al. Determinants of stillbirth in the Five General Hospitals of Lusaka, Zambia: A Case-Control study. Medical Journal of Zambia. 2023;50(1):35-46.
47. Dube K, Lavender T, Blaikie K, Sutton CJ, Heazell AE, Smyth RM. Identification of factors associated with stillbirth in Zimbabwe–a cross sectional study. BMC pregnancy and childbirth. 2021;21:1-14.
48. Gardosi J, Kady SM, McGeown P, Francis A, Tonks A. Classification of stillbirth by relevant condition at death (ReCoDe): population based cohort study. Bmj. 2005;331(7525):1113-7.
49. Heazell A, McLaughlin MJ, Schmidt E, Cox P, Flenady V, Khong T, et al. A difficult conversation? The views and experiences of parents and professionals on the consent process for perinatal postmortem after stillbirth. BJOG: An international journal of obstetrics & Gynaecology. 2012;119(8):987-97.
50. Chibwesha CJ, Zanolini A, Smid M, Vwalika B, Kasaro MP, Mwanahamuntu M, et al. Predictors and outcomes of low birth weight in Lusaka, Zambia. International Journal of Gynecology & Obstetrics. 2016;134(3):309-14.

~~55.~~

Formatted: Indent: Left: 0.25", No bullets or numbering

Dr Musonda Makasa

P.O Box 310129

Lusaka 10101

February 22, 2025

PLOS Global Public Health Staff
globalpubhealth@plos.org

Dear Editor,

Rebuttal Letter: Response to Reviewer #6 Comments for PGPH-D-24-01110R4
Predictors of perinatal mortality in the seven major hospitals of Lusaka Zambia: A Case Control Study
PLOS Global Public Health

We are grateful for the valuable comments provided by Reviewer #6, which have contributed significantly to improving the clarity and overall quality of our manuscript titled "Predictors of Perinatal Mortality in the Seven Major Hospitals of Lusaka Zambia: A Case-Control Study." We have carefully considered each remark and have made the necessary revisions. Below illustrates a point-by-point response to all the comments, highlighting the changes made in the manuscript:

Abstract:

- Standardize the decimals.
- **Response:** The typos have been addressed and corrected. Thank you for that eagle-eyed observation.

Findings:

1. **Table 1.0:** Please include totals; this will clarify your results and help with a better digestion and understanding of your results.
2. **Check your totals to ensure they reflect your sample size (n).**
3. **Standardize your decimals in the table and all results in the abstract and tables.**

Response to Reviewer's Concern on Table 1.0 Totals: Thank you for your insightful comment regarding the inclusion of totals in Table 1.0. We agree that adding totals will improve the clarity of the results and aid in their interpretation. We have now updated the table to include the totals for each category, as well as the correct calculations for the overall sample size.

Additionally, we have reviewed and ensured that the totals in the table align accurately with the sample size (n). We have made the necessary corrections, and the revised table should now provide a more transparent and digestible presentation of the data.

We also acknowledge that missing data may have been a potential limitation; however, we would like to emphasize that the proportion of missing data in this study did not exceed 6.7%. This percentage is within an acceptable range and unlikely to bias the results or significantly impact the overall analysis. Furthermore,

the study design included a 10% contingency in the sample size to account for potential dropouts or loss to follow-up, including participant withdrawal. This contingency ensured that the final analysis remained robust despite any missing data, and we believe the study's conclusions remain valid.

Lastly, we have corrected and standardized the use of decimals in both the abstract and results sections to ensure consistency and accuracy throughout the manuscript.

We hope these revisions address your concerns and enhance the clarity and robustness of the results.

Discussion:

1. **Discuss your study's socio-demographic objectives.**
2. **Try to compare and contrast your findings in a more understandable manner:** For example, the paragraph: "Furthermore, women using non-motorized transport were likely to access only Basic Emergency Obstetric and Neonatal Care (BEmONC) facilities, compared to those using motorized transport who had access to Comprehensive Emergency Obstetrics and Neonatal Care (CEmONC) facilities (40). As the latter offer superior care for pregnancy complications, especially if they have trained staff and are better equipped (41)," is not well explained in terms of how it relates to your findings. At which point did you refer? Is it higher or lower compared to your study?
3. **Discuss the study implications.**
4. **Ensure punctuation is well done in your discussion.**

Response to Reviewer's Comments: The socio-demographic objectives have been discussed and included in the discussion section.

The statement: "Furthermore, women using non-motorized transport were likely to access only Basic Emergency Obstetric and Neonatal Care (BEmONC) facilities, compared to those using motorized transport who had access to Comprehensive Emergency Obstetrics and Neonatal Care (CEmONC) facilities (40). As the latter offer superior care for pregnancy complications, especially if they have trained staff and are better equipped (41)," has been corrected and contextualized into a more meaningful manner. Kindly find the tracked changes manuscript version to ease reference.

A section discussing study implications was also worked on and incorporated after the discussion section.

Punctuation has been thoroughly reviewed and corrected.

Sincerely,

Musonda Makasa

***Corresponding author**

Dr Musonda Makasa

P.O Box 310129

Lusaka

April 20, 2025

PLOS Global Public Health Staff
globalpubhealth@plos.org

Dear Editor,

We would like to express our sincere appreciation to you and the reviewers for the thoughtful and constructive feedback on our manuscript titled: "**Predictors of Perinatal Mortality in Lusaka, Zambia: A Multi-Center Case-Control Study**" Manuscript ID: PGPH-D-24-01110R5

We are grateful for the opportunity to revise and improve our manuscript. We have carefully addressed all comments provided by the reviewers, and we believe the revised version of the manuscript has been significantly strengthened as a result.

Below, we provide a point-by-point response to each reviewer's comments, indicating how we have incorporated their suggestions into the revised manuscript. All changes have been tracked in the revised document.

Reviewer #6, #9

Comment: All comments were addressed.

Response: We thank Reviewer #6 for confirming that all comments have been satisfactorily addressed. No further action required.

Reviewer #7

General Comment:
We are grateful for the supportive and encouraging remarks. The reviewer recognized the study as well-designed, with compelling and impactful results. Below are responses to the specific recommendations:

Methods

1. **Birth estimates in Lusaka/hospitals:**
   Response: This study ethical clearance and approval boundaries were strictly on the seven major hospitals of Lusaka. These facilities are anchor the rest of the lower level facilities. Obtaining information on the total estimates would require another ethical clearance and approval tier. Obtaining this information from the Ministry of Health was therefore futile

as they cited that the information requested was beyond the scope and mandate of this investigation.

2. **Clarify reason for 5 excluded participants:**
   Response: We have updated the flow diagram and Methods section to include that 5 participants were withdrawn due to emotional distress.
3. **Clarify 7-day follow-up of controls:**
   Response: We have clarified in the Methods section that controls were followed up before discharge, and in rare cases during postnatal clinic visits within 7 days.

Stylistic Suggestions for Introduction and Methods

- **Reorganization and flow of introduction:**
  Response: The introduction has been restructured per the reviewer's suggestions. The background now opens with key statistics on perinatal mortality, followed by definitions, disparities across regions, and Zambia-specific context. The literature gap and study aim have also been made more prominent.
- **Sentence clarity and paragraph organization:**
  Response: We have revised, condensed, and reorganized several sentences and paragraphs for improved clarity and logical flow, as advised.
- **Revised MSB definition and deletion from discussion:**
  Response: We have clarified the definition of macerated stillbirth using "more than 8 hours prior to the onset of labor" and deleted the redundant sentence in the discussion.
- **Improved wording in Methods:**
  Response: The sentence in the Methods section has been revised to: *"Participants were prospectively recruited as cases occurred, with matched controls, and informed written consent was obtained from all participants."*

Copy-editing and Minor Corrections

All grammatical and stylistic corrections suggested—such as punctuation in the abstract, paragraph breaks in the introduction, and sentence refinement throughout—have been addressed. We have also standardized the use of "PMR" after its first full mention.

Reviewer #10

1. **Citations for statistics in the Introduction:**
   Response: We have added references to support all cited statistics, including the 2.6 million perinatal deaths figure.
2. **Updated references for maternal mortality and stillbirths:**
   Response: We have replaced older references with more recent data from WHO (2023) and the UN IGME 2023 Child Mortality Report. We will consider updating to the 2025 estimates if publication timing permits.
3. **Clarify maternal/perinatal death linkage:**
   Response: We have deleted the sentence citing references 10–12 and strengthened the paragraph using reference 13.

4. **Exclusion criteria – non-consent:**
   Response: We have explicitly stated in the Methods section that participants who did not provide consent were excluded.
5. **Clarify inclusion of known risk factors not discussed:**
   Response: We clarified that some known predictors (e.g., congenital anomalies, syphilis) were not analyzed due to data limitations. This has also been noted in the limitations section.

Reviewer #11

1. **Missing data and bias mitigation:**
   Response: We have elaborated in the Methods section on how missing data were handled and what strategies were used to minimize information bias (e.g., use of trained data collectors, real-time data checks).
2. **Variable selection in regression model:**
   Response: We have expanded the Data Analysis section to explain the rationale for selecting specific predictor variables based on prior literature and clinical relevance.
3. **Policy implications:**
   Response: A summary of policy implications and suggested interventions has been added to the Recommendations section to guide future programming.

Reviewer #12

Thank you for your supportive and constructive feedback. The following minor revisions have been made as suggested:

- **Abstract:** Revised the sentence for clarity: *"...compared to early booking."*
- **Introduction:** Revised to "...have exacerbated the issue."
- **Methods:** Revised for clarity: *"...controls were followed up before discharge."*
- **Results:** Revised sentence: *"...two times more likely..."*
- **Recommendations:** Spelled out "CEmONC" to ensure clarity for all readers.

We trust that the revised manuscript now fully addresses the concerns and suggestions raised by the reviewers. We appreciate your time and consideration and hope the revised version meets the standards for publication.

Sincerely,

Musonda Makasa

***Corresponding author**

On behalf of all co-authors
