# Supplementary material for: Predictors of perinatal mortality in the seven major hospitals of Lusaka Zambia: A case control study
Source: PLOS Glob Public Health. 2025 Aug 20;5(8):e0003326. doi: 10.1371/journal.pgph.0003326 (PMC12367193; doi:10.1371/journal.pgph.0003326)
Supplement: Attachment — Submitted filename: Rebuttal letter.docx [file pgph.0003326.s002.docx]

Dr Musonda Makasa

P.O Box 310129

Lusaka

25^th^ July 2024

PLOS Global Public Health Staff
[globalpubhealth@plos.org](mailto:globalpubhealth@plos.org)

Dear Editors/Reviewers

REF: PGPH-D-24-01110 Predictors of perinatal mortality in the seven major hospitals of Lusaka Zambia: A Case Control Study

Pursuant to the above.

I wish to express my gratitude to you, editors and reviewers, for taking your time on this article and the constructive feedback. This rebuttal letter serve to respond to the comments raised during the review process the revisions made as well as clarifications. The comments have been responded to in detail section by section as is demonstrated in the table below.

Yours sincerely,

Dr Musonda Makasa

| **Recommended revisions** | **Responses** |
| --- | --- |
| **Abstract**   1. In the opening statement, please clearly state that the 2.6million babies lost are at global level. State the timeline for this statistic - was this by end of 2015? By end of 2021? For which age is this? Within 28 days of life? Within 7 days of life? | Addressed in the abstract section and track changes maintained |
| **Abstract**  Rephrase the statement, "...... 5.7million since 2000", you probably meant, "5.7 million in 2000".  The statement, "... the rest are in low and middle income countries, 77% in sub-Saharan Africa" is unclear. The rest of what? 77% of what?  State the statistical package and statistical tests used.  There is need for consistency in reporting confidence intervals: one decimal vs two decimal places.  The last sentence of the Conclusion section is unclear: there is need to link the findings on the predictors with the outcome. Also, include a statement on the implication of the study findings. | These sections the abstract have been addressed appropriately with track changes maintained |
| **Introduction**  Please clearly define perinatal deaths at the outset. The authors are encouraged to be consistent in the use of the terms "early neonatal death" and "neonatal death" as these have different definitions.  The statement, "Attempts to avoid these perinatal deaths have not yielded much" is ambiguous. The authors are encouraged to use statistics to illustrate the progress Zambia has made in reducing perinatal mortality rate.  I suggest that the authors rewrite the second half of the first paragraph of the introduction - to maintain the focus on perinatal mortality - first present maternal death as a predictor of early neonatal mortality, before delving into the statistics on maternal mortality. Otherwise, in its current state, the logical flow of ideas on perinatal mortality is distorted.  Revise the last sentence of the introduction section: Additionally, the study also aimed to provide evidence to that guide program interventions to reduce the PMR in this setting." the portion, "... to that guide..." needs re-writing. | Addressed and definition provided appropriately in the Introduction section  Addressed as seen in second  Paragraph  Neonatal mortality in this section is mentioned just as a SDG target as the SDGs do not categorically state perinatal mortality. In this case the intention is just how the trend of reduction has been and since this in part captures early neonatal mortality.  Addressed |
| **Methodology section**  What do the authors mean by the statement, "The study did not involve minors"?  Please clarify who the study participants were. Provide a consistent definition of the cases and controls. Were the study subjects, the mothers of the infants or the infants themselves?  Is the Fertility rate of Lusaka similar to the national average of 4.4? You seem to suggest so.  Why was there no attempt to match the controls to the cases, especially by age? Were controls recruited from the same health facility as the cases?  Who provided informed consent? At what point during the postnatal period was data collection done? Was there need to translate the data collection tools?  Under study variables, clearly define the study outcome - perinatal death and the respective attributes. What was the operational definition of a fresh stillbirth? A macerated stillbirth? An early neonatal death?  For which of the exposure variables did you obtain data from chart review and from questionnaire interview? How was HIV/syphilis/malaria/tuberculosis/Rhesus/anemia status ascertained? Did you carry out any laboratory tests as part of the study? Findings on some of the variables are not included in the results. For example, what was the relationship between Rhesus status and the study outcome?    Page 10 - first line, what is "congenital louis"? Did you mean "congenital syphilis"? How was this diagnosed? How were congenital abnormalities diagnosed?  Please state the statistical tests used for bivariate analysis.  The statement, "Control was exclusive to the PI for daily monitoring and management in real time." is unclear. | Addressed participants were over 18 as assent was missed during ethical approval  Addressed as participants were mothers  Addressed. The statement was misapplied  Addressed as there was no guarantee to match by age within the set time of recruitment of a control following recruitment of a case.  Addressed as this was conducted by data collector (Midwives). Translation was provided in the very few instances required  Addressed and corrected  No laboratory tests were conducted. They were ascertained by reviewing records. Non inclusion of certain variables like Rhesus status was due to incompleteness in data capturing on some variables such that the analysis could not deduce  statistical generalization on the few that were captured. Hence the exclusion. This has been cited as a shortcoming in the limitations section  Addressed and corrected.  This variable was based on participants who had positive or negative syphilis tests from their records.  Addressed  Addressed and corrected |
| **Results section**  In the results section, it is important that you present the frequencies of the different sub-categories of the outcome: FSB, MSB, ENND? This is included in the second paragraph of the discussion section, which is improper. | Addressed |
| **Conclusions and recommendations sections** | Provided after the strengths and limitations sections |
