# Supplementary material for: Predictors of perinatal mortality in the seven major hospitals of Lusaka Zambia: A case control study
Source: PLOS Glob Public Health. 2025 Aug 20;5(8):e0003326. doi: 10.1371/journal.pgph.0003326 (PMC12367193; doi:10.1371/journal.pgph.0003326)
Supplement: Attachment — Submitted filename: Review commentb.docx [file pgph.0003326.s006.docx]

**Reviewer comment: Predictors of perinatal mortality in the seven major hospitals of Lusaka Zambia: A Case Control Study**

**Methodology**

All factors found to be associated with perinatal mortality in the bivariate analysis (p-value <0.05): add at 95% confidence interval.

**Findings:**

Review the title of **Table 1.0:** Summary of the maternal socio-demographic characteristics frequency distribution. it is not only socio-demographic characteristics. There are actually other factors such as comorbidities, lifestyle such as smoking and alcohol use. Review the title or split it into different tables.

**In table 1.0: missing percentage**

**ANC Booking:** one line misses percentage 106 (%)

**Preeclampsia**: misses percentage 160(%)

It would have been better to add total on each variable to track the missing values on each variable and checking if the total results correlated with included final sample size.

**Discussion:**

Discuss this study implication??
