# Supplementary material for: Predictors of perinatal mortality in the seven major hospitals of Lusaka Zambia: A case control study
Source: PLOS Glob Public Health. 2025 Aug 20;5(8):e0003326. doi: 10.1371/journal.pgph.0003326 (PMC12367193; doi:10.1371/journal.pgph.0003326)
Supplement: Attachment — Submitted filename: Response to Reviewers.docx [file pgph.0003326.s007.docx]

Dr Musonda Makasa

P.O Box 310129

Lusaka

January 19, 2025

PLOS Global Public Health Staff
[globalpubhealth@plos.org](mailto:globalpubhealth@plos.org)

Dear Editor,

**Rebuttal Letter: Response to Reviewer #6 Comments** for PGPH-D-24-01110R3
Predictors of perinatal mortality in the seven major hospitals of Lusaka Zambia: A Case Control Study
PLOS Global Public Health

We are grateful for the valuable comments provided by Reviewers #6 and #7, which have contributed significantly to improving the clarity and overall quality of our manuscript titled “Predictors of Perinatal Mortality in the Seven Major Hospitals of Lusaka Zambia: A Case-Control Study.” We have carefully considered each remark and have made the necessary revisions. Below illustrates a point-by-point response to all the comments, highlighting the changes made in the manuscript:

Recommendations:

Reviewer #6

Intro:

1. Consider streamlining the statistics presented in the introduction to improve readability. For example, while the statistics on maternal deaths and the absolute numbers of global perinatal mortality are noteworthy, they may be less directly relevant to the focus of this paper. Instead, incorporating a statistic on the current global neonatal mortality rate and its trends over time could provide context for the reader especially for comparison with the WHO sustainable development goals.

Response: this recommendation has been taken care of and the suggested statistics on the current global neonatal mortality trend has been provided in the introduction section

2. Consider providing a definition and brief overview of maceration in the introduction, and cite relevant references for the readers’ understanding.

Response: the definitions have been provided in the first paragraph of the introduction

Methods:

3. Consider specifying the total number of hospitals in Lusaka and describe the criteria used to select the facilities included in the study. Include an estimation of the number of births per year in Lusaka.

Response: Only the regulatory authorities have the information regarding the total number of hospitals. However, this study focused on the biggest hospitals of Lusaka district i.e. five first level hospitals and 2 tertiary hospitals. These hospitals are recipients of all perinatal cases with the potential to have complications. And therefore, while they are all referral hospitals, on the other hand, the five first level hospitals serve as satellite sites for the two tertiary hospitals as they are the only ones with neonatal intensive care services.

4. Please provide details on how study variable data was collected: through chart review? Through maternal interview?) If different variables were collected using different methods, please specify which were collected using what method.

Response: The process involved administering the participants’ information sheet first for those that met the inclusion criteria later on obtaining informed consent if agreeable. This questionnaire filling was preceded by collecting distal and intermediate characteristics from the files. The rest of the information unobtainable from the files was collated using interviewer-administered questionnaire by interviewing the mother.

5. Please provide details on the five participants who were excluded. Response: the five had change of countenance and mind due to emotional distress and decided not to proceed. However, all participants were only recruited after bereavement counselling.

6. Please clarify whether any infants greater than 24 weeks of gestation weighed less than 500 grams. Response: none of note

7. Include information about Group B Strep status and the duration of rupture of membranes (ROM) if available. If not, consider discussing the absence of this data in the discussion section.

Response: There is no information on Group B Strep status because this study did not carry out any experiments and neither was Group B Strep an independent variable.

8. Please provide details on data collection for the 7-day follow-up of the control group. Specifically, clarify how it was determined that the controls were healthy during this period.

Response: Unhealthy ones are triaged and immediately referred to the paediatrics hospital and so only healthy babies are reviewed during routine early-neonatal period.

9. Please include the researcher questionnaire as a supplemental file for transparency and reproducibility.

Response: The questionnaire can be shared on request as the information and details are still under the custody of the University of Zambia, School of Public Health

Results:

10. For Table 1, the percentages should be presented as intragroup values (e.g., percentages within cases for age groups ≤19, 20–34, >35, and similarly for controls) rather than intergroup comparisons (cases vs. controls) for clarity. Additionally, please include the sample size (N) at the top for both cases and controls. “Folate and Ferrous sulphate” heading should clarify that it is supplementation: “Folate and Ferrous sulphate supplementation”

Response: the corrections have been made accordingly and can be traced in the appropriate section

11. Please clarify whether "malaria" refers to a history of malaria (and if so, whether this was during pregnancy) or active malaria at the time of delivery. If data is available on the timing of malaria infection (ie, which trimester of pregnancy it occurred), please include this information.

Response: the correction has been made. The variable should read Malaria during pregnancy

12. Please include proximal level variables and their comparison in Table 1, consider including text in the results section as well.

Response: The table has been divided into distal, intermediate, and proximal variables

13. The description of the bivariate analysis using the chi-square test should be moved to the Methods section for consistency.

Response: This section has been moved to the methods section

14. Analyzing macerated vs. non-macerated cases could be interesting and provide additional insights into variables affecting stillbirth. Consider conducting a comparative subgroup analysis to identify the variables associated with each category.

Response: This was not part of the plan and neither was it an objective for this study. However, the comparison will be done in a different paper that will follow this one particular one under review. The paper following this one yet to be finalized with placental pathology embedded as analysis is still underway.

15. Consider further stratifying the analysis by hospital type or care level (tertiary vs first level hospitals) to explore potential variations across different healthcare settings.

Response: This can be considered for future research. However, anecdotally the intra-cluster correlation coefficient is quite high because of high similarities of socio-demographic and health characteristics

16. Please include data on the average number of days for early neonatal mortality in cases to provide additional context.

Response: This variable was not a continuous count but was categorized instead during data collection and so the distribution of deaths in terms of days was not done. However, this will be covered by yet another study underway, as it is being reviewed by the authorship team that also considered time to death.

Other comments:

A thorough copy-editing of the manuscript is recommended to address minor spelling and grammatical errors. Some specific (but not comprehensive) examples include:

• In the Abstract Methods section of the abstract, capitalize the first word (“This”).

Response: Corrected

• In the Abstract Results, revise the sentence to read: “...12 weeks gestation had almost three times the odds of experiencing perinatal mortality.” Additionally, place the p-value within the parentheses.

Response: Corrected

• In the same section, revise the sentence to read: “Walking as a means of reaching the healthcare facility had over three times the odds of perinatal mortality.”

Response: Corrected

• In the Introduction, the abbreviation “PMR” is introduced twice; only the first instance needs to be kept. Corrected

Response: corrected

• In the Study Design section, correct the repetition in the third line: “from from.”

Response: corrected and done

• In the Methods, revise “controls were ‘follow up’ before discharge” to “controls were followed up before discharge.”

Response: corrected

• In the Inclusion Criteria section, correct the formatting to avoid having two periods after the first sentence.

Response: corrected

• In the Patients’ Socio-Demographic Characteristics section, capitalize the first letter of religions.

Response: corrected

• In the same section, when discussing socio-demographic characteristics, avoid simply listing the numbers; either include them in parenthesis or include a description.

Response: corrected

We are grateful to the reviewers for their constructive feedback, which has greatly strengthened the rigor and clarity of our study. We believe the revised manuscript now provides a clearer, more accurate presentation of our methods and findings.

Thank you for considering our responses and revisions. We look forward to your further feedback.

Sincerely,

**Musonda Makasa**

***Corresponding author**
