# Supplementary material for: Predictors of perinatal mortality in the seven major hospitals of Lusaka Zambia: A case control study
Source: PLOS Glob Public Health. 2025 Aug 20;5(8):e0003326. doi: 10.1371/journal.pgph.0003326 (PMC12367193; doi:10.1371/journal.pgph.0003326)
Supplement: Attachment — Submitted filename: Review report 28 Jan 2025.docx [file pgph.0003326.s008.docx]

**Review report:**

**Abstract:**

Standardize the decimals.

**Findings:**

i. Table1.0: Please include totals this will clarify your results and help a good digestion and understanding of your results.
ii. Check your totals are not reflecting your (n)
iii. Standardize your decimals in the table and in all your results in abstract and tables.

**Discussion:**

i. Discuss your study socio-demographic objectives.
ii. Try to compare and contrast your findings in understandable manner: for example, this paragraph:(Furthermore, women using non-motorized transport were likely to access only Basic Emergency Obstetric and Neonatal Care (BEmONC) facilities, compared to those using motorized transport who had access to Comprehensive Emergency Obstetrics and Neonatal Care (CEmONC) facilities (40). As the latter offer superior care for pregnancy complications especially if they have trained staff and are better equipped (41)) is not well explained how it is related to your findings. At which point you referred? is it high or low compared to your study?
iii. Discuss the study implications
iv. Ensure punctuation is well done in your discussion.
