# Supplementary material for: Predictors of perinatal mortality in the seven major hospitals of Lusaka Zambia: A case control study
Source: PLOS Glob Public Health. 2025 Aug 20;5(8):e0003326. doi: 10.1371/journal.pgph.0003326 (PMC12367193; doi:10.1371/journal.pgph.0003326)
Supplement: Attachment — Submitted filename: Response_to_Reviewers_auresp_5.docx [file pgph.0003326.s009.docx]

Dr Musonda Makasa

P.O Box 310129

Lusaka 10101

February 22, 2025

PLOS Global Public Health Staff
[globalpubhealth@plos.org](mailto:globalpubhealth@plos.org)

Dear Editor,

**Rebuttal Letter: Response to Reviewer #6 Comments** for PGPH-D-24-01110R4
Predictors of perinatal mortality in the seven major hospitals of Lusaka Zambia: A Case Control Study
PLOS Global Public Health

We are grateful for the valuable comments provided by Reviewer #6, which have contributed significantly to improving the clarity and overall quality of our manuscript titled “Predictors of Perinatal Mortality in the Seven Major Hospitals of Lusaka Zambia: A Case-Control Study.” We have carefully considered each remark and have made the necessary revisions. Below illustrates a point-by-point response to all the comments, highlighting the changes made in the manuscript:

**Abstract:**

- Standardize the decimals.
- **Response:** The typos have been addressed and corrected. Thank you for that eagle-eyed observation.

**Findings:**

1. **Table 1.0:** Please include totals; this will clarify your results and help with a better digestion and understanding of your results.
2. **Check your totals to ensure they reflect your sample size (n).**
3. **Standardize your decimals in the table and all results in the abstract and tables.**

**Response to Reviewer’s Concern on Table 1.0 Totals:** Thank you for your insightful comment regarding the inclusion of totals in Table 1.0. We agree that adding totals will improve the clarity of the results and aid in their interpretation. We have now updated the table to include the totals for each category, as well as the correct calculations for the overall sample size.

Additionally, we have reviewed and ensured that the totals in the table align accurately with the sample size (n). We have made the necessary corrections, and the revised table should now provide a more transparent and digestible presentation of the data.

We also acknowledge that missing data may have been a potential limitation; however, we would like to emphasize that the proportion of missing data in this study did not exceed 6.7%. This percentage is within an acceptable range and unlikely to bias the results or significantly impact the overall analysis. Furthermore, the study design included a 10% contingency in the sample size to account for potential dropouts or loss to follow-up, including participant withdrawal. This contingency ensured that the final analysis remained robust despite any missing data, and we believe the study’s conclusions remain valid.

Lastly, we have corrected and standardized the use of decimals in both the abstract and results sections to ensure consistency and accuracy throughout the manuscript.

We hope these revisions address your concerns and enhance the clarity and robustness of the results.

**Discussion:**

1. **Discuss your study's socio-demographic objectives.**
2. **Try to compare and contrast your findings in a more understandable manner:** For example, the paragraph: “Furthermore, women using non-motorized transport were likely to access only Basic Emergency Obstetric and Neonatal Care (BEmONC) facilities, compared to those using motorized transport who had access to Comprehensive Emergency Obstetrics and Neonatal Care (CEmONC) facilities (40). As the latter offer superior care for pregnancy complications, especially if they have trained staff and are better equipped (41),” is not well explained in terms of how it relates to your findings. At which point did you refer? Is it higher or lower compared to your study?
3. **Discuss the study implications.**
4. **Ensure punctuation is well done in your discussion.**

**Response to Reviewer’s Comments:** The socio-demographic objectives have been discussed and included in the discussion section.

The statement: "Furthermore, women using non-motorized transport were likely to access only Basic Emergency Obstetric and Neonatal Care (BEmONC) facilities, compared to those using motorized transport who had access to Comprehensive Emergency Obstetrics and Neonatal Care (CEmONC) facilities (40). As the latter offer superior care for pregnancy complications, especially if they have trained staff and are better equipped (41)," has been corrected and contextualized into a more meaningful manner. Kindly find the tracked changes manuscript version to ease reference.

A section discussing study implications was also worked on and incorporated after the discussion section.

Punctuation has been thoroughly reviewed and corrected.

Sincerely,

**Musonda Makasa**

***Corresponding author**
