# Supplementary material for: Predictors of perinatal mortality in the seven major hospitals of Lusaka Zambia: A case control study
Source: PLOS Glob Public Health. 2025 Aug 20;5(8):e0003326. doi: 10.1371/journal.pgph.0003326 (PMC12367193; doi:10.1371/journal.pgph.0003326)
Supplement: Attachment — Submitted filename: Reviewed Manuscript.docx [file pgph.0003326.s010.docx]

# Predictors of perinatal mortality in the seven major hospitals of Lusaka Zambia: A Case Control Study

Makasa Musonda*^1,^ ^2,^ ^4^, Kaonga Patrick^1^, Jacobs Choolwe^1^, Makasa Mpundu^3^, Vwalika Bellingtone^4^

^1^University of Zambia School of Public Health, Department of Epidemiology and Biostatistics, Lusaka, Zambia

^2^University Teaching Hospital, Women and Newborn Hospital, Lusaka, Zambia

^3^University of Zambia School of Public Health Department of Community and Family Medicine, Lusaka, Zambia

^4^University of Zambia School of Medicine Department of Obstetrics and Gynaecology, Lusaka, Zambia

**Authors**

**Musonda Makasa – *Corresponding author**

Email: [mcmakasa@gmail.com](mailto:mcmakasa@gmail.com)

Patrick Kaonga

Email: [patrickkaonga@gmail.com](mailto:patrickkaonga@gmail.com)

Choolwe Jacobs

Email: [choolwe2003@yahoo.com](mailto:choolwe2003@yahoo.com)

Mpundu Makasa

Email: [cmakasa@yahoo.com](mailto:cmakasa@yahoo.com)

Bellington Vwalika

Email: [vwalikab@gmail.com](mailto:vwalikab@gmail.com)

**Abstract**

**Background:** Over 2.6 million babies globally are lost before birth or in the first week of life due to ??. Global perinatal mortality reduced from 5.7 million in 2000 to 4.1 million by end of 2015, of which 45% were recorded in high-income countries and the rest are in low-income countries. Perinatal mortality rates for sub-Saharan Africa and Zambia are at 37.4 and 33/1000 live births, respectively. The aim of this study was to determine the predictors of perinatal mortality at the seven major hospitals of Lusaka, Zambia. **Methods:** This was a multi-centre unmatched case control study from September 2023 to January 2024. Cases included perinatal death (>24 weeks gestation or >500g stillborn and death of neonate within seven days of life) and controls were live births. Stepwise multivariate logistic regression analysis with Stata version 14 determined predictors using adjusted odd ratios and p-values. **Results:** This study included 630 participants, with 210 cases and 420 controls analysed in a 1:2 ratio. Antenatal care booking after 12 weeks gestation had almost three times odds of experiencing perinatal mortality (AOR 2.91, 95% CI: 1.97-4.29), p

<0.001) compared to early booking. Walking as a means of reaching the healthcare facility had over three times the odds of perinatal mortality (AOR3.48, 95% CI: 1.87-6.49, p<0.012) compared to using personal transport. Anaemia in pregnancy carried a three-and-a-half-fold increased the risk of perinatal death (AOR 3.58, 95% CI: 1.72-7.44, p<0.001) compared to the non-anaemic?. History of previous pregnancy loss was associated with a five-fold increased risk of perinatal death (AOR 5.05, 95% CI: 2.99-8.51, p<0.001) compared to those without such a history. **Conclusion:** This study revealed that late antenatal care booking, walking, as means of transport to access health facility, anaemia in pregnancy, and previous history of loss of baby before birth perinatal death were the main predictors with statistical significance of perinatal death experience.

**Keywords: Perinatal Mortality, Predictors, Antenatal Care, Anaemia, Case-Control Study**

# Introduction

Perinatal mortality refers to the loss of a foetus after 24 weeks of gestation, or with a birthweight

≥500g; as well as the death of a newborn within the first 7 days of life. Perinatal mortality comprises of stillbirths and early neonatal deaths. Early neonatal mortality specifically refers to the death of a newborn baby within the first 7 days of life (1). Stillbirth on the other hand is intrauterine death before onset of labour or during labour (2). Stillbirths consist of fresh and macerated stillbirths. A fresh stillbirth (FSB) does not have skin changes by the time of delivery and is presumed to have died more recently during labour (intrapartum). Whereas, a macerated stillbirth (MSB) shows skin and soft tissue changes such as skin discolouration, peeling, and breakdown which is suggestive of death occurring well before onset of labour (2-4). Perinatal mortality remains a significant global health challenge particularly in low-income settings (5). An estimated 2.6 million babies are lost annually during pregnancy, labour and or in the first seven days of birth. Efforts to reduce perinatal deaths in line with the World Health Organization (WHO) Sustainable Development Goals (SDG) target has yielded limited successes, particularly in sub-Saharan Africa (SSA). Recently, most stillbirths were excluded from global data tracking, and the lack of social recognition, investment and programmatic action has exacerbated the issue (6). In 2017, the Maternal Mortality Estimation Interagency Group (MMEIG) reported 295,000 maternal deaths worldwide, with 196,000 (66%) occurring in SSA(7). In low-income settings, over a third of these maternal deaths and half of the stillbirths occur before or during childbirth (8, 9).

Literature shows (10,12) a strong link between maternal deaths and perinatal mortality because for every maternal death, there is an estimated 10 perinatal deaths. The authors also reported that nearly two thirds of causes of maternal death also contribute to perinatal deaths (13). SDG number 3.2 aims to end preventable newborn deaths by reducing neonatal mortality rate to at least 12 per 1000 live births by 2030 (5). The global perinatal mortality reduced from 5.7 million since the launch of the Millennium Development Goals(MDGs) in 2000 to 4.1 million by the time of the launch of the SDGs (14). Similarly, the global stillbirth rate also declined from 24.7 in 2000 to 18.4 per 1000 live births in 2015 (15). WHO Neonatal and Perinatal Mortality Report, 2000 indicated 47 per 1000 live births global rate of perinatal mortality and 62 per 1000 for Africa

(1). These strides have only been realistic in High-Income Countries (HIC) that even account for only approximately 45% of the global records on perinatal mortality (16). For example, in Hong Kong, the rate of perinatal mortality is 3.4 per 1000 live births and 5.54 per 1000 in the United States of America (17, 18). In stark contrast, SSA has remained the highest Perinatal Mortality Rate (PMR) (37.31 per 1000 live births) the highest reported in Cote D’Ivoire 68.1 per 1000 live births according to a recent system review and meta-analysis (19). In southern Africa PMR is approximately 30.3 per 1000 live births (20,21).

Zambia is not immune to the ongoing struggle with high perinatal mortality rates. The country’s PMR stands at 33 per 1000 live births (22), which exceeds the average reported for Southern Africa and remains well short of the target set by the SDG and Zambia’s Vision 2030 (23). This study aimed to assess the predictors of perinatal death in all the major hospitals of Lusaka urban district, the most populous in Zambia with the highest birth rate. Additionally, the research sought to provide evidence that could inform program interventions aimed at reducing the PMR in this context.

Objectives

……………………………………………………………………………………………………………………………………………………………………………………………………………………………………………………………………………………………………………………………………………………………………………………………………………………………………………………………….

# Materials and Methods

This was a multicentre study conducted at the seven major hospitals in Lusaka urban district, Zambia. Data collection commenced on the 9^th^ of September in 2023 and concluded on January

31^st^ in 2024. Participants were prospectively recruited as cases occurred with controls. Eligible participants who were agreeable to participate after being provided with detailed information via a participant information sheet were then recruited after obtaining informed written consent. The participants included mothers who had perinatal deaths (cases) and those with live births (controls) were followed up before discharge. Data was collected using an interviewer-structured questionnaire. The hospitals involved all the major hospitals of Lusaka which include: two tertiary facilities - Women and Newborn and Levy Mwanawasa University Teaching Hospitals - and five first level hospitals: Chawama, Chilenje, Chipata, Kanyama, and Matero first level hospitals. According to the Census Report, 2022 the Lusaka is the most populous city in Zambia, with a female population of 1,590,922 (24).

# Study design

This was an unmatched prospective case-control study with a 1:2 ratio, aimed at determining the predictors of perinatal mortality at the selected study site. Cases were defined as stillbirth after 24 weeks of gestation; ≥500g where gestational age was not clear; and early neonatal deaths within seven days of life from the study sites. For each case, two controls were selected through systematic random sampling, involving women who had live births immediately before or after occurrence of a case. Controls were recruited from the same facility where the cases occurred, and for early neonatal deaths, controls were mothers who delivered within 24hrs of case’s birth.

A midwife from each labour and postnatal ward at each facility was trained and enrolled as data a collector. Data collectors provided participants with an information sheet and obtained informed consent. In rare cases where translation was necessary, data collectors translated the information into the appropriate language that participants understood and were comfortable with. Data collection for postnatal mothers occurred whenever a case was identified (within the early neonatal

period) and controls were subsequently recruited. In rare cases especially following an early neonatal death, controls were followed up before discharge or during clinic visits, ensuring their recruitment around the time the case occurred. Data were collected by reviewing files followed by interviewer administered collection of data using a standardized structured questionnaire on a google sheet that was managed by the Principal Investigator (PI). The PI monitored the Google data collection sheet on a daily basis.

**Study population**

Total study participants n =630

**Excluded**

Five participants did not meet the inclusion criteria

# Study population

Study population comprised women aged 18 years and older who sought and received maternal and/or childbirth services at the study sites and met the inclusion criteria. Refer to the following: figure 1.0 flowchart illustrates the source of the sample, eligibility screening process and how the final sample was arrived at.

**Eligible population**

Women who had stillbirths and early neonatal deaths (cases) at the hospitals and controls were women and babies who delivered live babies within 24 hours of cases

**Source population**

All women giving birth at the selected study sites (October 2023 to January 2024)

**All cases**

(Perinatal deaths) n=210

**Random controls**

(Live births) n=420

**Figure 1.0** *Selection of study participants’ flow chart*

# Inclusion criteria

Cases included stillbirths from mothers aged above 18 years, with a gestational age above 24 weeks. In the absence of gestational age a birthweight of ≥500g was considered eligible for this study. Early neonatal deaths were defined as deaths occurring within seven days of birth. Controls consisted of women above 18 years with live births, recruited within 24 hours of a corresponding case.

# Exclusion criteria

Women were excluded if they withdrew from the study, were under the age of 18, had a pregnancy of less than 24 weeks of gestation, or in the absence of gestational age, had a birthweight of less than 500g. Additionally, women were excluded if they were unavailable during the study period, were unable to participate due to ill health, or were too emotionally distressed to provide reliable information, even after reviewing the participant information sheet.

# Sample size calculation

The sample size for the study was calculated based on the assumptions that two controls per care (r = 2), with 80% power of the study (type II error 20%, i.e. β = 0.2), confidence interval of 95% with 5% type I error (i.e. α = 0.05). With an assumed odds ratio of 2.5 for differences based on findings in previous similar studies. They reported for example associated potential risk factors included extremes of birthweight, post term delivery, infections, haemorrhage and previous history

or perinatal mortality (25, 26). And using Zambia’s PMR of 33 per 1000 live births (22), the following formula by Charan and Biswas (27) was used:

𝑛 =

𝑟 + 1

𝑟

𝑝^∗^(1 − 𝑝^∗^)(𝑍_𝛽_ + 𝑍𝛼/ )^2^

�	2	�

(𝑃_1_ − 𝑃_2_)^2^

Where 𝑛 = is the sample size, 𝑝^∗^average proportion exposed which was given by the sum of

proportion exposed cases (𝑃 ) and proportion of control 𝑃 divided by 2 (i.e. = ^𝑃1+𝑃2^), 𝑍 is the

1	2	_2_	𝛽

standard normal value for power ( for power of 80%, 𝑍_𝛽_ = 0.84), 𝑍𝛼/2 is the standard normal value for the level of significance which is usually given as 𝛼=0.05 and it’s value is 1.96, and r is the ratio of controls to cases, in our case the ratio is 2 to 1 which means our r = 2; To find P2, we use the formula:

P2 =	𝑂𝑂𝑅×𝑃1	, where OR is the odds ratio and P1 is the proportion with a specific risk factor

𝑃1(𝑂𝑂𝑅−1)+1

in the control participants. Assuming OR = 3.2 based on pooled analysis with strong association of perinatal mortality due to lack of antenatal care (13): P1=0.033 in the formula above we get the value of P2 as 0.09845

P*= 0.033+0.09845= 0.13145 = 0.0657

2	2

Replacing the above information in the prescribed formula above the following was the targeted sample size

𝑛 =

2 + 1

�

2

0.0657(1 − 0.0657)(0.84 + 1.96)^2^

(0.09845 − 0.033)^2^	�

𝑛 = 168.4874

𝑛 = 169

Therefore, the calculated sample size was 169 cases and 338 using the 1:2 ratio, which gave 507. After factoring in 10% additional in case of participant fall out, the total sample size calculated was 558.

# Sampling technique

To determine the sample size for each facility, using Probability Proportional to Size (PPS) sampling, we accounted for the delivery volume at each facility to ensure that larger facilities, which manage more deliveries, had a proportionally higher chance of selection. Hence, the following was applied in our study:

1. **To establishing facility delivery size**: We first reviewed delivery records from each facility covering January to December 2023 to estimate the delivery size at each one. This information served as the basis for determining the relative size of each facility.
2. **To ensure proportional allocation**: Facilities with higher delivery numbers were assigned a greater probability of selection compared to those with fewer deliveries. This allocation ensured that our sample would reflect the distribution of delivery cases across facilities, avoiding under-representation from larger facilities.
3. **To sample participants within facilities**: Once the facilities were selected with PPS, we then identified participants within each facility for inclusion in the study. This selection process within facilities allowed us to capture representative samples proportionate to the overall patient volume, aligning with the facility's estimated annual deliveries.

By using PPS sampling, we ensured a sample distribution that mirrors the scale of service utilization across facilities, thereby enhancing the representativeness and reliability of our findings.

A total of 635 participants were initially enrolled across all study sites. However, only 630 participants were included in the final analysis, as the remainder did not meet the inclusion criteria because they exceeded the early neonatal period. Data collection was through an interviewer administered questionnaire using a password controlled google sheet. Due to rarity of the outcome, purposeful sampling was used for the cases that met the inclusion criteria. For each case enrolled, two controls were randomly selected within the same 24-hour shift in which the case occurred.

# Study variables

The outcome variables include macerated (a fresh stillbirth with features of maceration), fresh stillbirths (without features of maceration), and early neonatal death (occurred within 7 days of life). Distal level variables included age, marital status, parity, gravida, weight, BMI, employment status, educational level, distance of place of residence to health facility, and mode of transport. Intermediate level variables were antenatal booking data, number of antenatal contacts, medical history, history of pregnancy loss beyond viability, history of abortion, malaria during pregnancy, Human Immunodeficiency Virus (HIV) status, tuberculosis during pregnancy, syphilis, alcohol intake during pregnancy, illicit drugs, smoking, folic and ferrous sulphate use during pregnancy, malaria treatment during pregnancy, and sickle cell anaemia. Rhesus factor, antepartum haemorrhage, preeclampsia, and eclampsia, preterm delivery, history of low birth weight, and inter-pregnancy interval were the rest. While proximal level variables included gestational age at the time of birth, outcome, Apgar score, time lag to Neonatal Intensive Care Unit (NICU), age of early neonatal at the time of demise, sex, gross congenital anomalies, congenital syphilis, prematurity, low birthweight, and cause of death.

# Data collection tool

A standard researcher administered questionnaire was set up using the google sheet platform with protected access to the selected data collectors only. The PI exclusively handled daily monitoring in real-time. After completion of data collection the data set was downloaded to an excel format for screening and cleaning and then coded to construct a do file for execution in Stata version 14.

# Data analysis

Descriptive statistical analyses were conducted to summarize the proportions and frequencies of cases and controls. A bivariate analysis using Chi-square test was performed to identify the factors associated with perinatal mortality. All factors found to be associated with perinatal mortality in the bivariate analysis (*p-*value <0.05) at 95% confidence interval (95% CI) were then included in multivariable logistic regression model to adjust for potential confounders. Sequential elimination of predictors was employed to assess the strength and significance of the variables associated with perinatal deaths, followed by the discrimination and calibration. Continuous variables such as age, weight, parity, and gravidity were categorized based on clinical reasoning before being introduced into the model.

The ability of the model to correct risk was assessed using the Hosmer and Lemeshow goodness of fit test. A non-significant *p-*value (*p*>0.05) from this test indicated a good fit. Model stability was further evaluated by the measurement of the discrimination between participants with and without perinatal deaths using the area under the receiver operating characteristic (ROC) curve, with an acceptable values et at 0.7 (28). The study followed the Strengthening the Reporting of Observational Studies in Epidemiology (STROBE) guidelines (29).

# Ethical considerations

Ethical approval for this study was obtained from the University of Zambia Biomedical Research Ethics Committee (UNZABREC), reference number 3712-2023. Prior to this, clearance was secured from the National Health Research Authority (NHRA), under reference number NHRA 000012/16/03/2023. All procedures adhered to the ethical guidelines for research involving human participants, including informed consent. Participants were thoroughly briefed about the study’s purposed, procedures, potential risks, and benefits, and written informed consent obtained before enrolment.

Confidentiality and privacy were strictly maintained throughout the study. All personal identifying information was anonymized, and the data collected was stored on password-protected devices accessible only to the principal investigator (PI) and designated research team members. To ensure data security, encrypted backups were created, and physical access to data storage location was restricted. Participants were also informed of their right to withdraw from the study at any point without any consequences. The study adhered to national and international research ethics standards.

# Results

**Description of study participants**

Macerated	Early neonatal deaths

**Macerated	Early neonatal deaths**

Fresh still births

**Fresh still births**

0

**36**

40

20

**56**

120

100

80

60

**118**

140

DISTRIBUTION OF PERINATAL DEATHS

***Figure 2.0*** *distribution of perinatal mortality according to outcomes*

The total perinatal deaths 210 (cases) and 420 with live births (controls) made the ratio of cases to controls 1:2. The proportion of the perinatal outcomes were Early Neonatal Death (ENND) 118 (56%), Macerate Stillbirth (MSB) 56 (27 %), and 36 (17%) Fresh Stillbirths (FSB) respectively.

# Participants’ socio-demographic characteristics

From the social-demographic attributes (distal level variables) of the respondents sampled majority of the respondents were between the age of 20-34 years 401 (63.7%). 19 years old and under were 90 (14.3%) and 97 (15.4%) were 35 years of age and above. Mean age for the study population was 27. Of the respondents 478 (75.9%) were Married and 143 (22.7%) were not married. A total of 487 (75.9%) had a parity between 1 to 4, while 81 (12.9%) were grandmultipara. Additionally, 445 (72.4%) were not employed and 170 (27.6%) employed. For education, the majority of the

respondents had only attained secondary level of education 331 (52.5%), primary 187 (29.7%), and 95 (15.1%) for tertiary. Most of the participants used public transport 415 (65.9%) while only 98 (15.6%) had personal transport and the others 106 (16.8%) on foot.

**Results of predictors of perinatal mortality from bivariate analysis**

Bivariate analysis using Chi-Square Test revealed several variable associated with perinatal mortality. The outcomes outlined in the table below revealed that the level of education of the mother had a significant association with perinatal mortality (p<0.017). The form of transport used when going to the hospital (on foot) also had association with perinatal mortality (p<0.001). Antenatal Care (ANC) booking (first contact with healthcare provider for ANC) beyond 12 weeks of gestation was statistically significant with p<0.001. Women who had prior history of perinatal death also had a statistically significant p-value (p<0.001). Abortion and Anaemia during pregnancy were found with p <0.018 and p<0.003 respectively. The rest of the independent variables had no statistical significance.

**Table 1.0 Summary of the maternal socio-demographic characteristics and pregnancy factors associated perinatal mortality**

| **Predictors** | **Cases (n) 210 (%)** | **Controls (n) 420 (%)** | **Total (n) 630 (%)** | **χ² p-value** |
| --- | --- | --- | --- | --- |
| **Distal level variables** | | | | |
| **Age** |  |  |  |  |
| ≤ 19 | 30 (14.3) | 60 (14.3) | 90 (14.3) |  |
| 20-34 | 138 (65.7) | 263 (62.6) | 401 (63.7) | 0.466 |
| > 35  Missing | 27 (12.9)  15 (7.1) | 70 (16.7)  27 (6.4) | 97 (15.4)  42 (6.7) |  |
| **Marital status** |  |  |  |  |
| Not married | 50 (23.8) | 93 (22.1) | 143 (22.7) | 0.741 |
| Married Missing | 160 (76.2)  0 (0.0) | 318 (75.7)  9 (2.1) | 478 (75.9)  9 (1.4) |  |
| **Parity** |  |  |  |  |
| 1 – 4 | 170 (81.0) | 309 (73.6) | 487 (77.3) | 0.702 |
| > 5  Missing | 36 (17.1)  4 (1.9) | 62 (14.8)  49 (11.7) | 81 (12.9)  62 (9.8) |  |

| **Employment**  **Status** |  |  |  |  |
| --- | --- | --- | --- | --- |
| Not employment | 151 (71.9) | 294 (70.0) | 445 (72.4) | 0.610 |
| Employed Missing | 54 (25.7)  5 (2.4) | 116 (27.6)  10 (2.4) | 170 (27.6)  15 (2.4) |  |
| **Educational level** |  |  |  |  |
| Primary | 65 (31.0) | 122 (29.0) | 187 (29.7) |  |
| Secondary | 121 (57.6) | 210 (50.0) | 331 (52.5) | **0.017*** |
| Tertiary Missing | 20 (9.6)  4 (1.9) | 75 (17.9)  13 (3.1) | 95 (15.1)  17 (2.7) |  |
| **Intermediate level variables Mode of transport** | | | | |
| Foot | 50 (23.8) | 56 (13.3) | 106 (16.8) | **0.001*** |
| Public transport | 139 (66.2) | 276 (65.7) | 415 (65.9) |  |
| Personal Missing | 20 (9.5)  1 (0.5) | 78 (18.6)  10 (2.4) | 98 (15.6)  11 (1.7) |  |
| **ANC Booking** |  |  |  |  |
| ˂ 12 weeks | 100 (47.6) | 83 (19.8) | 183 (29.0) |  |
| > 12 weeks Missing | 106 (50.5)  4 (1.9) | 323 (76.9)  14 (3.3) | 430 (68.3)  17 (2.7) | **0.001*** |
| **History of Stillbirth** |  |  |  |  |
| Yes | 64 (30.5) | 32 (7.6) | 96 (15.2) | **0.001*** |
| No Missing | 143 (68.1)  3 (1.4) | 377 (89.8)  11 (2.6) | 520 (82.5)  14 (2.2) |  |
| **History of Abortion** |  |  |  |  |
| Yes | 10 (4.8) | 7 (1.7) | 17 (2.7) | **0.018*** |
| No Missing | 181 (86.2)  19 (9.0) | 393 (93.6)  20 (4.8) | 574 (91.1)  39 (6.2) |  |
| **Proximal level variables Malaria**  **during pregnancy** | | | | |
| Yes | 21 (10.0) | 26 (6.2) | 47 (7.5) | 0.080 |
| No Missing | 181 (86.2)  8 (3.8) | 384 (91.4)  10 (2.4) | 565 (89.7)  18 (2.9) |  |
| **HIV Status** |  |  |  |  |
| Positive | 30 (14.3) | 59 (14.0) | 89 (14.1) | 0.898 |
| Negative Missing | 173 (82.4)  7 (3.3) | 351 (83.6)  10 (2.4) | 524 (83.2)  17 (2.7) |  |
| **Tuberculosis during pregnancy** |  |  |  |  |
| Yes | 2 (1.0) | 1 (0.2) | 3 (0.5) | 0.227 |
| No Missing | 201 (95.7)  7 (3.3) | 397 (94.5)  22 (5.2) | 598 (94.9)  29 (4.6) |  |
| **Alcohol** |  |  |  |  |

| Yes | 14 (6.7) | 42 (10.0) | 56 (8.9) | 0.155 |
| --- | --- | --- | --- | --- |
| No Missing | 193 (91.9)  3 (1.4) | 368 (87.6)  10 2.4) | 561 (89.0)  13 (2.1) |  |
| **Smoking** |  |  |  |  |
| Yes | 4 (1.9) | 2 (0.5) | 6 (1.0) | 0.087 |
| No Missing | 204 (97.1)  2 (1.0) | 405 (96.4)  13 (3.1) | 609 (96.7)  15 (2.4) |  |
| **Folate and Ferrous sulphate supplementation** | | | | |
| Yes | 197 (93.8) | 396 (94.3) | 593 (94.1) | 0.307 |
| No Missing | 10 (4.8)  3 (1.4) | 13 (3.1)  11 (2.6) | 23 (3.7)  14 (2.2) |  |
| **Anaemia in pregnancy** |  |  |  |  |
| Yes | 13 (6.2) | 60 (14.3) | 73 (11.6) | **0.003*** |
| No Missing | 190 (90.5)  7 (3.3) | 349 (83.1)  11 (2.6) | 539 (85.6)  18 (2.9) |  |
| **Sickle cell anaemia** | | | | |
| Yes | 1 (0.5) | 8 (1.9) | 9 (1.0) | 0.151 |
| No Missing | 205 (97.6)  4 (1.9) | 400 (95.2)  12 (2.9) | 605 (96.0)  19 (3.0) |  |
| **Antepartum Haemorrhage** |  |  |  |  |
| Yes | 11 (5.2) | 43 (10.2) | 54 (8.6) | **0.058** |
| No Missing | 176 (83.8)  23 (11.0) | 358 (85.2)  19 (4.5) | 534 (84.8)  42 (6.7) |  |
| **Preeclampsia** |  |  |  |  |
| Yes | 20 (9.5) | 47 (11.2) | 67 (10.6) | 0.863 |
| No Missing | 160 (76.2)  30 (14.3) | 358 (85.2)  15 (3.6) | 518 (82.2)  45 (7.1) |  |
| **Hypertension** |  |  |  |  |
| Yes | 14 (6.7) | 16 (3.8) | 30 (4.8) | 0.101 |
| No Missing | 187 (89.0)  9 (4.3) | 393 (93.6)  11 (2.6) | 580 (92.1)  20 (3.2) |  |
| **Diabetes Mellitus** | | | | |
| Yes | 1 (0.5) | 2 (0.5) | 3 (0.5) | 0.994 |
| No  Missing | 204 (97.1)  5 (2.4) | 404 (96.2)  14 (3.3) | 608 (96.5)  19 (3.0) |  |

**Unadjusted logistic regression of variables that are predictors of perinatal mortality**

According to the bivariate logistic regression for predictors of perinatal death (table 2), respondents with a primary education level were almost two times likely to experience perinatal mortality compared to those the tertiary level group (COR=2.00, 95% CI: 1.12-3.56). The analysis also showed that the odds of experiencing perinatal death were over three times higher (COR=3.48, 95% CI: 1.87-6.49) for women who walked to access healthcare services and nearly two times as high (COR=1.96, 95% CI: 1.15-3.34) for those using public transport, compared to women who had personal transport.

Additionally, starting ANC after 12 weeks of gestation had over three and half odds (COR=3.68, 95% CI: 2.56-5.30) of perinatal mortality compared to those that started earlier. Women with previous history of stillbirth were almost five times more likely to experience perinatal mortality (COR=5.27, 95 CI: 3.31-8.402) compared to those without such a history. Women with history of abortion were also three times more likely (OR=3.10, 95% CI: 1.16-8.28) to experience perinatal mortality compared to those who had no history of abortion before. This study also found that women who experienced anaemia during pregnancy had more than two and half times higher odds of perinatal mortality (COR=2.51, 95 CI: 1.34-4.69) compared to those without anaemia.

In order to understand the combined impact of predictors of perinatal mortality and to control for confounding, it required multivariate logistic regression analysis. Table 2.0 is the illustration of the final multivariate model that best describes predictors of perinatal mortality. Following a thorough analysis and correction for the impact of extraneous variables, four variables stood out as predictors of perinatal mortality.

While educational level after multivariate logistic regression analysis did not show significance as a predictor of perinatal mortality. Mode of transport to the healthcare facility, on the other hand, showed significance as a predictor of perinatal mortality. Women who walked to the hospital had three times the odds of perinatal mortality compared to those who used personal transport (AOR 3.48, 95% CI: 1.87–6.49, p < 0.001). Antenatal care (ANC) booking after 12 weeks of gestation was significantly associated with an increased risk of perinatal mortality. Women who booked ANC late had nearly three times higher odds of experiencing perinatal mortality compared to those who booked early (AOR 2.91, 95% CI: 1.97–4.29, p < 0.001). Maternal anemia during pregnancy was associated with a three-and-a-half-fold increased risk of perinatal mortality. Women with anemia had significantly higher odds of perinatal death compared to non-anemic women (AOR 3.58, 95% CI: 1.72–7.44, p < 0.001). A history of previous pregnancy loss was strongly associated with increased odds of perinatal mortality. Women with a history of stillbirth had five times the odds of perinatal death compared to those without such a history (AOR 5.05, 95% CI: 2.99–8.51, p < 0.001). Table 2.0 presents a summarized comparison of univariable and multivariable predictors following bivariable and then multivariable logistic regression analysis.

**Table 2.0: Multivariable logistic regression analysis for factors associated with perinatal mortality in the selected study sites**

| **Univariable logistic regression** | |  |  | **Multivariable logistic regression analysis** | | |
| --- | --- | --- | --- | --- | --- | --- |
| **Predictor variable** | **Crude Odds Ratio (COR)** | **95%CI** | **p- value** | **Adjusted Odds Ratio (AOR)** | **95% CI** | **P-value** |
| **Educational level** |  |  |  |  |  |  |
| Tertiary Primary  Secondary | Ref 2.00  2.16 | (1.12-3.56)  (1.26-3.71) | 0.019  0.005 | 1.66  1.87 | (0.83-3.31)  (0.98-3.57) | 0.152  **0.058** |
| **Mode of Transport** | |  |  |  |  |  |

| Personal Foot Public | Ref 3.48  1.96 | (1.87-6.49)  (1.15-3.34) | <0.001  0.013 | 2.75  1.70 | (1.23-5.39)  (0.92-3.16) | **0.012***  0.091 |
| --- | --- | --- | --- | --- | --- | --- |
| **ANC Booking** | | | | | | |
| < 12 weeks  > 12 weeks | Ref  3.68 | (2.56-5.30) | <0.001^*^ | Ref  2.91 | (1.97-4.3) | **<0.001^*^** |
| **History of Stillbirth** |  |  |  |  |  |  |
| No  Yes | Ref  5.27 | (3.31-8.40) | <0.001^*^ | Ref  5.05 | (2.99-8.51) | **<0.001^*^** |
| **History of**  **abortion** | | | | | | |
| No  Yes | Ref  3.10 | (1.16-8.28) | 0.024 | 0.86 | (0.24-3.06) | 0.814 |
| **Anaemia during pregnancy** |  |  |  |  |  |  |
| No  Yes | Ref  2.51 | (1.34-4.69) | 0.004 | Ref  3.58 | (1.72-7.44) | **0.001^*^** |

# Discussion

The objective of this study was to identify predictors of perinatal mortality in Lusaka, Zambia. The selected sites are the major hospitals of Lusaka and referral hospitals that anchor all maternal and perinatal health services. Among the socio-demographic variables, educational level and mode of transport were the only variables strongly associated with perinatal mortality. The statistically significant predictors of perinatal mortality identified in this study were mode of transport used by women when accessing healthcare, timing of antenatal care (ANC) bookings, history of abortion, stillbirth, and anaemia during pregnancy.

In clinical settings, pregnancy in women of advanced maternal age (AMA) (i.e. maternal age >35) is frequently labelled as “high-risk even in the absence of obvious risk factors. This classification has been validated by many authors from a meta-analysis on “advanced maternal age: adverse outcomes of pregnancy”, where it was reported that AMA women had higher perinatal mortality and stillbirths compared to women of 20 – 34 maternal age group (30). On the contrary, in this

study maternal age was not found to have significant association with perinatal mortality. Educational level and mode of transport are the other socio-demographic factors that demonstrated association with our outcome of interest. However, during univariable and multivariable logistic regression analysis only the mode of transport remained statistically significant as a predictor of perinatal mortality. While education level did not show strong association with perinatal mortality in this investigation. A study on effect of maternal education and perinatal outcome done in Punjab, India reported that lower educational status was a significantly important predictor of adverse perinatal outcomes including perinatal mortality (31).

This study revealed that macerated stillbirths and early neonatal deaths (ENND) had a higher occurrence among the perinatal mortality cases, consistent with findings reported in other studies (32, 33). Another study (34) similarly reported a higher proportion of macerated stillbirths compared to fresh stillbirths, which may reflect the quality of prenatal and obstetric care, while macerated stillbirths are typically the results of intrauterine foetal death occurring more than 8 hours prior to labour (35). Contributing factors to macerated stillbirths include intrauterine growth restriction, placental lesions, and infections, though a large proportion of these deaths remain unexplained (36). In this study, these cases also remained unexplained, underscoring the need for further investigation into the underlying causes and improved data collection practices. Early neonatal deaths are often caused by complications such as low birth weight, birth asphyxia, and sepsis, which require neonatal intensive care (37,39). However, in this study, the causes of early neonatal mortality could not be determined because of the nature of the study design that could measure association.

Among the distal level variables none of them had statistical significance as a predictors of perinatal mortality. On the other hand it was mainly the intermediate level variables that had

significant association with perinatal mortality: mode of transport, ANC booking, and history of stillbirth. Whereas only anaemia during pregnancy was found to be a significant predictor of perinatal mortality among the proximal level variables. The mode of transport showed statistical significance as a predictor of perinatal mortality. This was because women who walked to the healthcare facility to seek medical care during pregnancy were found to be three times more likely to experience perinatal death compared to those with personal transportation. Those using public transport also had two times higher risk of perinatal mortality experience. This finding aligns with a previous study done in Zambia and Uganda, which identified a significant difference in perinatal outcomes based on motorized vs non-motorized means of transport (40). Factors contributing to this difference may include poor physical access to healthcare, lack of empowerment for decision- making, and limited health education (41). Furthermore, women who relied on non-motorized transport, such as walking to seek medical care - as was observed in this study - were more likely to access Basic Emergency Obstetric and Neonatal Care (BEmONC) facilities. In contrast, those using motorized transport who had access to Comprehensive Emergency Obstetrics and Neonatal Care (CEmONC) facilities, which provide superior care and improved maternal and perinatal outcomes, particularly when staffed with trained personnel and equipped with essential resources (42, 43).

The timing of ANC bookings also played a significant role in perinatal outcomes. The WHO recommends that the first ANC visit should occur within the first trimester, up to 12 weeks of gestation (44). In this study, over 70% of the women booked late for ANC (> 12 weeks), and late booking was associated with a threefold increased risk of perinatal mortality. This finding corroborated other studies, which reported that delayed ANC increases the risk of complications during pregnancy and delivery, contributing to perinatal mortality (45, 46).

A previous history of abortion did not emerge as a significant predictor of perinatal mortality in this study. However, a history of abortion can increase the risk of perinatal mortality compared to those without such a history. This may be attributed to a higher susceptibility to infections during subsequent pregnancies among women with history of abortion (47), which raises the likelihood of perinatal mortality (48). Conversely, previous history of stillbirth, in this study, was significantly associated with a fivefold increase in the risk of perinatal mortality. This finding aligns with results from other studies in Zambia (49) and Zimbabwe (50), which similarly reported that women with a history of stillbirth faced higher risk of perinatal mortality. This also aligns with findings from a study conducted in Ethiopia (47), which demonstrated that women with a history of stillbirth were at a higher risk of infections during subsequent pregnancies. These infections can compromise foetal health, leading to complications that significantly elevate the likelihood of perinatal mortality (48). Despite such findings from other studies linking infections to perinatal mortality, infections were not significantly associated with perinatal mortality in this study. Additionally, many stillbirths remain unexplained, largely due to the low availability of post-mortem investigations (51). This lack of clear causality makes discussing stillbirths challenging, not only for the parents but also for healthcare professionals, who often face difficulties in obtaining consent for post-mortem investigation aimed at determining the cause of death (52).

Among the medical complications, anaemia during pregnancy was the strongest predictor of perinatal mortality, with women suffering from anaemia showing a two-and-a-half-fold increased risk, compared to those without anaemia. Anaemia is closely linked to small-for-gestational-age infants and preterm birth, both of which are significant risk factors for perinatal mortality (49).

While previous studies in Zambia (25, 53) identified small-for-gestational-age infants, preterm birth, and low birth weight as high-risk factors for perinatal mortality, this study did not find these findings to have significant association with perinatal mortality.

# Study Implications

Based on our study findings, socio-demographic factors like lower education level (primary and secondary) and mode of transport showed association with higher likelihood of perinatal mortality, although the latter was not statistically significant in the adjusted model. Participants with personal transport were significantly associated with lower odds of perinatal mortality even in the adjusted multivariable logistic regression model compared to those using public or walked to seek healthcare service. This may be suggestive of other factors like lower socioeconomic status and transport accessibility play a significant role in influencing pregnancy outcomes. Further interventions should focus on tackling such socio-demographic issues like improving transport accessibility and education.

The study also highlighted several other factors, such as late ANC booking, a history of stillbirth, and anaemia during pregnancy, which remained significantly associated with higher odds of perinatal mortality even after multivariable regression analysis. These findings imply that health policies should prioritize improving early ANC attendance and targeting high-risk pregnancies, particularly such as anaemia in pregnancy and history of stillbirth should be the centre theme for developing interventions for positive pregnancy outcomes. In this vein, future research should explore potential interventions to reduce the effects of these predictors and improve perinatal outcomes.

# Strengths and Limitations

This study provides a comprehensive assessment of multiple predictors of perinatal mortality simultaneously, offering a nuanced understanding of the factors influencing perinatal outcomes in Lusaka district. The sample is representative of the broader population, as data were collected from all major hospitals in the city, ensuring a diverse range of cases that enhances the generalizability of the findings. This broad representation strengthens the relevance of the study to policymakers and healthcare practitioners, as the insights derived can inform targeted interventions and resource allocation in public health strategies. Furthermore, identifying significant predictors not only contributes to the existing body of literature but also equips clinicians with evidence-based knowledge to improve maternal and neonatal care practices.

One notable limitation is the incompleteness of information during data collection, primarily due to inconsistencies in medical record-keeping, and data entry errors which resulted in gaps in some variables. Additionally, accurately stating or recording the cause of death in stillbirth cases remains challenging, as investigations are not routinely conducted to determine the cause of death. These limitations highlight the need for improved data collection practices and post-mortem investigations to enhance the accuracy and reliability of future research in this area.

# Conclusion

In conclusion, this study highlights several critical predictors of perinatal mortality in Lusaka, Zambia, including mode of transport, timing of antenatal care booking, previous stillbirths, and maternal anaemia. Women who walked or used public transport to access healthcare faced higher risks of perinatal mortality, underscoring the socioeconomic disparities affecting access to quality obstetric care. The late initiation of antenatal care compromised early detection and management

of complications, emphasizing the need for public health initiatives to promote timely antenatal care. Additionally, history of stillbirth significantly increased the risk of subsequent perinatal mortality, highlighting the necessity for psychological and medical support for women with previous adverse pregnancy outcomes. Maternal anaemia was also a substantial risk factor, correlating with adverse birth outcomes such as small for gestational age and preterm births. While the study did not find small for gestational age or preterm birth to be direct predictors, these findings align with existing literature. Overall, targeted interventions addressing these predictors— such as improving transportation options, enhancing antenatal care accessibility, and providing support for women with prior adverse pregnancy experiences—could significantly reduce perinatal mortality rates and improve maternal and neonatal health outcomes in Lusaka. The aim of this study was to identify the predictors of perinatal mortality in Lusaka, Zambia.

# Recommendations

According to this study findings, in order to help to reduce the perinatal mortality in Lusaka, Zambia: there is need to improve transportation to enhance options for pregnant women especially in the underserved areas; need to ensure timely access to CEmONC facilities; Early ANC booking needs to be promoted through strengthening education and outreach programs to encourage women to book for ANC within the first trimester of pregnancy. In addition, to address anaemia during pregnancy routine screening and treatment need to be strengthened to reduce the associated risks to perinatal mortality. Women with previous experience of perinatal mortality need targeted support and monitoring to minimise risks in subsequent pregnancies. This support includes but not limited to enhanced quality prenatal and obstetrics care investment focusing on early detection and management of pregnancy complications. Finally, community-based health education and

empowerment programs to equip women with knowledge and resources for better pregnancy outcomes and self-autonomy in decision-making.

# Acknowledgements

I would like to thank all my supervisors for the guidance provided to complete this study. I also would like to acknowledge the immense contribution the study participants for accepting to be part of the project.

# Conflict of interest

We declare no conflict of interest.

# Authors’ contributions

MM: conceptualization of the study, study design, data collection, data cleaning, data processing and analysis, and manuscript writing. PK: supervision and methodology. CJ: supervision and contextualization of the project. MM: conceptualization and contextualization of the study. BV: overall content supervision, contextualization of the study, and methodology. All authors have read and approved the final version of the manuscript.

# References

1. Organization WH. Neonatal and perinatal mortality: country, regional and global estimates: World Health Organization; 2006.
2. Gold KJ, Abdul-Mumin AR, Boggs ME, Opare-Addo HS, Lieberman RW. Assessment of "fresh" versus "macerated" as accurate markers of time since intrauterine fetal demise in low-income countries. Int J Gynaecol Obstet. 2014;125(3):223-7.
3. Lawn JE, Gravett MG, Nunes TM, Rubens CE, Stanton C, Group GR. Global report on preterm birth and stillbirth (1 of 7): definitions, description of the burden and opportunities to improve data. BMC pregnancy and childbirth. 2010;10:1-22.
4. Shanklin DR. FETAL MACERATION. 2. AN ANALYSIS OF 53 HUMAN STILLBORN INFANTS. Am J

Obstet Gynecol. 1964;88:224-9.

5. Organization WH. Health in 2015 from MDGs, Millenium Development Goals to SDGs, Sustaibale Development Goals. Health from MDGs to SDGs. 2015;1(2015):216-.
6. De Bernis L, Kinney MV, Stones W, ten Hoope-Bender P, Vivio D, Leisher SH, et al. Stillbirths: ending preventable deaths by 2030. The lancet. 2016;387(10019):703-16.
7. Organization WH. Trends in maternal mortality 2000 to 2017: estimates by WHO, UNICEF, UNFPA, World Bank Group and the United Nations Population Division. 2019.
8. Say L, Chou D, Gemmill A, Tunçalp Ö, Moller AB, Daniels J, et al. Global causes of maternal death: A WHO systematic analysis. The Lancet Global Health. 2014;2(6):323-33.
9. Lawn JE, Blencowe H, Waiswa P, Amouzou A, Mathers C, Hogan D, et al. Stillbirths: rates, risk factors and potential for progress towards 2030. Lancet. 2016;387(10018):587-603.
10. Gaym A. Perinatal mortality audit at Jimma hospital, South-Western Ethiopia, 1990-1999. The Ethiopian Journal of Health Development. 2000;14(3).
11. Briand V, Dumont A, Abrahamowicz M, Sow A, Traore M, Rozenberg P, et al. Maternal and perinatal outcomes by mode of delivery in Senegal and Mali: a cross-sectional epidemiological survey. 2012.
12. Aragaw Y. Perinatal mortality and associated factor in Jimma university specialized hospital, South West Ethiopia. Gynecol Obstet (Sunnyvale). 2016;6(409):2161-0932.
13. Berhan Y, Berhan A. Meta-analysis of selected maternal and fetal factors for perinatal mortality. Ethiopian journal of health sciences. 2014;24:55-68.
14. Wang H, Bhutta ZA, Coates MM, Coggeshall M, Dandona L, Diallo K, et al. Global, regional, national, and selected subnational levels of stillbirths, neonatal, infant, and under-5 mortality, 1980– 2015: a systematic analysis for the Global Burden of Disease Study 2015. The Lancet. 2016;388(10053):1725-74.
15. De Bernis L, Kinney MV, Stones W, Ten Hoope-Bender P, Vivio D, Leisher SH, et al. Stillbirths: Ending preventable deaths by 2030. The Lancet: Lancet Publishing Group; 2016. p. 703-16.
16. Blencowe H, Cousens S, Jassir FB, Say L, Chou D, Mathers C, et al. National, regional, and worldwide estimates of stillbirth rates in 2015, with trends from 2000: a systematic analysis. The Lancet Global Health. 2016;4(2):e98-e108.
17. Kumsa H, Mislu E, Yimer NB. A systematic review and meta-analysis of the globally reported International Classification of Diseases to Perinatal Mortality (ICD-PM). Frontiers in medicine. 2024;11:1434380.
18. Valenzuela CP, Gregory EC, Martin JA. Perinatal Mortality in the United States, 2020–2021: US Department of Health and Human Services, Centers for Disease Control and …; 2023.
19. Bezie MM, Asebe HA, Asnake AA, Fente BM, Negussie YM, Asmare ZA, et al. Factors associated with perinatal mortality in sub-Saharan Africa: A multilevel analysis. PloS one. 2024;19(11):e0314096.
20. Akombi BJ, Renzaho AM. Perinatal mortality in sub-saharan africa: A meta-analysis of demographic and health surveys. Annals of Global Health. 2019;85(1):1-8.
21. Tiruneh D, Assefa N, Mengiste B. Perinatal mortality and its determinants in Sub Saharan African countries: systematic review and meta-analysis. Maternal Health, Neonatology and Perinatology. 2021;7(1):1-17.
22. Central Statistics Office MoH. Zambia Demographic and Health Survey. 2018. Report No.: 9788578110796.
23. MoF GRZ. Vision 2030. Lusaka: Republic of Zambia; 2006.
24. Worldbank. Fertility rate, total (births per woman) - Zambia. THE World Bank - IBRD - IDA2020.
25. Stringer EM, Vwalika B, Killam WP, Giganti MJ, Mbewe R, Benjamin BH, et al. Determinants of stillbirth in Zambia. Obstetrics and Gynecology. 2011;117(5):1151-9.
26. Chuwa FS, Mwanamsangu AH, Brown BG, Msuya SE, Senkoro EE, Mnali OP, et al. Maternal and fetal risk factors for stillbirth in Northern Tanzania: A registry-based retrospective cohort study. PloS one. 2017;12(8):e0182250-e.
27. Charan J, Biswas T. How to calculate sample size for different study designs in medical research? Indian journal of psychological medicine. 2013;35(2):121-6.
28. Kleinbaum DG, Klein M. Survival analysis: a self-learning text: Springer; 2012.
29. Von Elm E, Altman DG, Egger M, Pocock SJ, Gøtzsche PC, Vandenbroucke JP, et al. The Strengthening the Reporting of Observational Studies in Epidemiology (STROBE) statement: guidelines for reporting observational studies. Annals of internal medicine. 2007;147(8):573-7.
30. Pinheiro RL, Areia AL, Mota Pinto A, Donato H. Advanced maternal age: adverse outcomes of pregnancy, a meta-analysis. Acta medica portuguesa. 2019;32(3):219-26.
31. Chopra I, Juneja SK, Sharma S. Effect of maternal education on antenatal care utilization, maternal and perinatal outcome in a tertiary care hospital. Int J Reprod Contraception, Obstet Gynecol. 2018;8(1):247.
32. Abdulhameed AL, Aljammas EK, Ramzi A. Perinatal mortality types. Sulaymania, Iraq: Department of Family and Community Medicine, College of Medicine. 2016:366-72.
33. Mustufa MA, Kulsoom S, Sameen I, Moorani KN, Memon AA, Korejo R. Frequency of Stillbirths in a Tertiary Care Hospital, Karachi. Pak J Med Sci. 2016;32(1):91-4.
34. Shelke SP, Shivkumar PV, Chaurasia AK. Incidence of stillbirth in relation to period of delivery, socioeconomic status and period of gestation in a rural tertiary care hospital-mgims, sewagram within study period of three years (2013–2016). The Journal of Obstetrics and Gynecology of India. 2021:1-5.
35. Langley F. The perinatal postmortem examination. Journal of clinical pathology. 1971;24(2):159- 69.
36. Man J, Hutchinson JC, Heazell AE, Ashworth M, Levine S, Sebire NJ. Stillbirth and intrauterine fetal death: factors affecting determination of cause of death at autopsy. Ultrasound in Obstetrics & Gynecology. 2016;48(5):566-73.
37. Gaíva MAM, Bittencourt RM, Fujimori E. Early and late neonatal death: characteristics of mothers and newborn. Revista Gaúcha de Enfermagem. 2013;34:91-7.
38. Ahmed Tahir Ahmed Abdifatah Elmi F. Determinants of early neonatal mortality (hospital based retrospective cohort study in Somali region of Ethiopia). Determinants of early neonatal mortality (hospital based retrospective cohort study in Somali region of Ethiopia). 2023;13(1114):8.
39. Bogale TN, Worku AG, Bikis GA, Kebede ZT. Why gone too soon? Examining social determinants of neonatal deaths in northwest Ethiopia using the three delay model approach. BMC Pediatrics. 2017;17(1):216.
40. Sacks E, Vail D, Austin-Evelyn K, Greeson D, Atuyambe LM, Macwan’gi M, et al. Factors influencing modes of transport and travel time for obstetric care: a mixed methods study in Zambia and Uganda. Health Policy and Planning. 2015;31(3):293-301.
41. Ayalew Tiruneh G, Melkamu Asaye M, Solomon AA, Tiruneh Arega D. Delays during emergency obstetric care and their determinants among mothers who gave birth in South Gondar zone hospitals, Ethiopia. A cross-sectional study design. Global Health Action. 2021;14(1):1953242.
42. Widyantari KY, Sulistyaningsih I, editors. The Effectiveness of Cemonc on Maternal Mortality: Systematic Literature Review. Proceeding International Conference; 2019.
43. Yusrawati Y, Respati T, Sekarwana N. Factors to improve the quality of comprehensive emergency obstetric and newborn care. International Journal of Public Health. 2023;12(3):1030-9.
44. Organization WH. WHO antenatal care recommendations for a positive pregnancy experience: nutritional interventions update: multiple micronutrient supplements during pregnancy. 2020.
45. Girum T. Alternative & Integrative Medicine. 2016.
46. Carroli G, Rooney C, Villar J. How effective is antenatal care in preventing maternal mortality and serious morbidity? An overview of the evidence. Paediatric and perinatal Epidemiology. 2001;15:1-42.
47. Feleke BE, Feleke TE. A longitudinal study on the effects of previous stillbirth or abortion on subsequent pregnancies and infants. European journal of public health. 2021;31(6):1237-41.
48. Suleiman MB, Mokuolu OA. Perinatal mortality in a Northwestern Nigerian city: a wake up call. Frontiers in Pediatrics. 2014;2:105.
49. Makasa M, Mutale W, Lubeya MK, Mpetemoya T, Chinayi M, Mangala B, et al. Determinants of stillbirth in the Five General Hospitals of Lusaka, Zambia: A Case-Control study. Medical Journal of Zambia. 2023;50(1):35-46.
50. Dube K, Lavender T, Blaikie K, Sutton CJ, Heazell AE, Smyth RM. Identification of factors associated with stillbirth in Zimbabwe–a cross sectional study. BMC pregnancy and childbirth. 2021;21:1-14.
51. Gardosi J, Kady SM, McGeown P, Francis A, Tonks A. Classification of stillbirth by relevant condition at death (ReCoDe): population based cohort study. Bmj. 2005;331(7525):1113-7.
52. Heazell A, McLaughlin MJ, Schmidt E, Cox P, Flenady V, Khong T, et al. A difficult conversation? The views and experiences of parents and professionals on the consent process for perinatal postmortem after stillbirth. BJOG: An international journal of obstetrics & Gynaecology. 2012;119(8):987-97.
53. Chibwesha CJ, Zanolini A, Smid M, Vwalika B, Kasaro MP, Mwanahamuntu M, et al. Predictors and outcomes of low birth weight in Lusaka, Zambia. International Journal of Gynecology & Obstetrics. 2016;134(3):309-14.
