# Supplementary material for: Predictors of perinatal mortality in the seven major hospitals of Lusaka Zambia: A case control study
Source: PLOS Glob Public Health. 2025 Aug 20;5(8):e0003326. doi: 10.1371/journal.pgph.0003326 (PMC12367193; doi:10.1371/journal.pgph.0003326)
Supplement: Attachment — Submitted filename: Response_to_Reviewers_auresp_7.docx [file pgph.0003326.s014.docx]

Dr Musonda Makasa

P.O Box 310129

Lusaka

June 16, 2025

PLOS Global Public Health Staff
[globalpubhealth@plos.org](mailto:globalpubhealth@plos.org)

Dear Editor,

We would like to express our sincere appreciation to you and the reviewers for the thoughtful and constructive feedback on our manuscript titled**: Predictors of perinatal mortality in the seven major hospitals of Lusaka Zambia: A Case Control Study - PGPH-D-24-01110R6**

We are grateful for the opportunity to revise and improve our manuscript. We have carefully addressed all comments provided by the reviewers, and we believe the revised version of the manuscript has been significantly strengthened as a result.

Below, we provide a point-by-point response to each reviewer’s comments, indicating how we have incorporated their suggestions into the revised manuscript. All changes have been tracked in the revised document.

**Response to Reviewer #2:**

Thank you for your detailed review and constructive comments. We have carefully addressed all the comments you provided in the annotated PDF. The corresponding revisions have been incorporated into the manuscript, and all changes can be followed through the track changes feature in the revised document. We appreciate your valuable feedback, which has significantly improved the quality and clarity of the manuscript.

**Regarding the comment on SDG:** - Thank you for this important observation. Indeed, while there has been notable progress in reducing neonatal mortality since the launch of the Sustainable Development Goals (SDGs), stillbirth prevention has not advanced at a comparable pace. Recent literature acknowledges this gap, with increasing advocacy to close the coverage and data gaps for stillbirths. The manuscript has been updated to reflect recent global efforts, including the integration of stillbirth reduction targets into the Every Newborn Action Plan and the renewed commitments through the ENAP 2025 update. These emphasize the need for accelerated action to achieve the stillbirth target of ≤12 per 1,000 births by 2030.

**Regarding the summary of objectives**: Thank you for this insightful observation. The original decomposition of the objectives section was based on recommendations from a previous reviewer, intended to enhance clarity by breaking down the study aims. However, in response to your suggestion, the section has been revised to present the objectives in a more concise and integrated manner within the final paragraph of the introduction.

**Response on the Study Implications:** Thank you for the comment. The implications were not integrated within the discussion paragraphs as per your suggestion because a previous reviewer had specifically recommended presenting them as a standalone section to enhance clarity and emphasis. We have therefore retained this structure in line with that guidance.

**Response to Strengths and Limitations and Recommendations**
Thank you for your feedback. The comments provided on the **Strengths and Limitations** and **Recommendations** sections have been carefully reviewed, and the respective sections have been revised appropriately in accordance with your observations.

**Response to Reviewer #10:**

We thank Reviewer #10 for the valuable feedback and detailed observations, which have helped to strengthen the accuracy and relevance of the manuscript. We have carefully revised the opening section in line with the comments, and the changes can be followed in the updated manuscript with track changes. Specific responses are as follows:

1. **Outdated data in the opening section:**
   We acknowledge the use of older data in the initial version. The background section has now been updated to include the most recent global estimates on stillbirths and newborn mortality as of 2025, to ensure alignment with current needs and priorities. We have also cited authoritative sources accordingly.
2. **Incorrect attribution of the Sustainable Development Goals (SDGs):**
   Thank you for highlighting this important correction. We have revised the manuscript to correctly attribute the SDGs to the **United Nations**, rather than WHO, in all relevant sections. The referencing has also been updated to reflect accurate source citations.
3. **Use of WHO classification for stillbirth:**
   We have now applied the **ICD-11 definition of stillbirth**, which aligns with the WHO classification: “a baby who dies after 28 completed weeks of gestation but before or during birth.” The incorrect definition has been removed, and the reference to the WHO stillbirth classification has been properly cited using the official WHO health topics resource.
4. **Inclusion of stillbirths as a target in Every Newborn Action Plan (ENAP):**
   We have now clearly noted in the introduction that stillbirths were officially included as a target in Every **Newborn Action Plan**, endorsed through the **World Health Assembly Resolution WHA67.10 (2014)**. The appropriate reference to the WHA Resolution has been added to support this statement.

**Response to Reviewer #13:**

We sincerely thank Reviewer #13 for the positive feedback and insightful observation regarding the potential influence of marital status on care-seeking behaviour and perinatal outcomes.

While we agree that marital status may play a role in shaping maternal health experiences, in our study it did not show a statistically significant association with perinatal mortality (p = 0.741), and therefore was not included as a major discussion point.

Nonetheless, we acknowledge the importance of exploring social determinants such as marital status further. We suggest that future qualitative studies could offer deeper insights into how relationship status may influence access to care, decision-making, and maternal outcomes in the Zambian context.

**Response to Reviewer #14:**

We thank Reviewer #14 for the feedback.

- We appreciate the reviewer’s observation. The suggestion to treat **socio-demographic variables** separately has been addressed by categorizing study variables into distal, intermediate, and proximal levels in the methods section, with socio-demographic variables clearly defined as distal-level factors
- The explanation on **handling missing data** has been expanded for clarity in the analysis section.
- The **sample size calculation** sentence has been corrected to read *"two controls per case."*
- The **bold formatting for the education variable** in the multivariate model has been removed, as the p-value was not significant.

These changes can be tracked in the revised manuscript.

**Concluding Remarks:**

We sincerely thank the editor and all reviewers for their time, thoughtful comments, and constructive guidance throughout the review process. The feedback has significantly strengthened the clarity, accuracy, and overall quality of the manuscript. We have carefully considered and incorporated the latest suggestions to the extent possible while balancing prior recommendations, some of which presented differing viewpoints. We remain committed to upholding the scientific rigor and relevance of our work and trust that the revised manuscript now meets the journal’s standards. We are grateful for the opportunity to contribute to this important discourse on perinatal health and look forward to your final evaluation.

Yours sincerely,

Dr. Musonda Makasa

Corresponding Author
