# Supplementary material for: Predictors of perinatal mortality in the seven major hospitals of Lusaka Zambia: A case control study
Source: PLOS Glob Public Health. 2025 Aug 20;5(8):e0003326. doi: 10.1371/journal.pgph.0003326 (PMC12367193; doi:10.1371/journal.pgph.0003326)
Supplement: Attachment — Submitted filename: Reviewer comment.docx [file pgph.0003326.s004.docx]

**Review comment:** **Predictors of perinatal mortality in the seven major hospitals of Lusaka Zambia: A Case Control Study**

**Methodology:**

**Study design:**

- Precise if the study design was prospective case control.
- Take this sentence in sampling technique:” due to rarity of the outcome purposeful sampling was used for the case that met the inclusive criteria.”

**Sampling Technique:**

- Put more information on how you used PPS: Probability Proportional to size sampling: was this technique used to select major hospital in the city or participants: if it was used to select major hospitals just show how it was applied. Then for the issue of representativity why not using proportionate stratified random sampling for selecting people from 7 hospitals:
- The final selected participants must correlate with the final sample size and the findings total.
- Your final selected participants were 630 Study participants, your final sample size is 558 of study participants and the total of findings are also showing different results. Just try to match these different parts for example apply the sample correction factor or explain why you have taken more study participants and if there were missing participants in findings.

**Data analysis:**

- Put the chi-square test used in data analysis, write also that all factors associated with perinatal death in bivariate analysis (Chi-square) were transferred to Logistic regression for confounder adjustment.
- Avoid the confusion of the word univariate analysis and bivariate analysis. Because univariate is for descriptive analysis.

**Findings:**

- The logistic regression can use only crude odd ratio: Bivariate and Adjusted Odd ratio (for multivariate). Just remove the confusion in your commentary.
